# Genetic architecture of cytokine autoantibodies and associated risk of common diseases

**DOI:** 10.1038/s41467-026-76394-7

**Published:** 2026-08-22

**Authors:** Jakob Hjorth von Stemann, Joseph Dowsett, Rebecca Svanberg Teglgaard, Maria Didriksen, Frederikke Byron Pedersen, Mette Skou Bentsen, Andrea Barghetti, Maria Vispe Laguarda, Sara Louise Borregaard Larsen, Bente Glintborg, Ulrik Lassen, Ruth Frikke-Schmidt, Celeste Porsbjerg, Jens-Ulrik Stæhr Jensen, Thomas Werge, Andrew J. Schork, Johan Skov Bundgaard, Henning Bundgaard, Torben Hansen, DBDS Genomic Consortium, Joseph Dowsett, Maria Didriksen, Frederikke Byron Pedersen, Mette Skou Bentsen, Andrea Barghetti, Thomas Werge, Andrew J. Schork, Johan Skov Bundgaard, Jakob Bay, Jens Kjærgaard Boldsen, Søren Brunak, Nanna Brøns, Alfonso Buil Demur, Lea Arregui Nordahl Christoffersen, Khoa Manh Dinh, Josephine Gladov, Daniel F. Gudbjartsson, Thomas Folkmann Hansen, Dorte Helenius Mikkelsen, Lotte Hindhede, Henrik Hjalgrim, Jakob Hjorth von Stemann, Kathrine Agergård Kaspersen, Bertram Dalskov Kjerulff, Lisette Kogelman, Mette Kongstad, Susan Mikkelsen, Line Hjorth Stjernholm Nielsen, Janna Nissen, Mette Nyegaard, Liam James Elgaard Quinn, Þórunn Rafnar, Palle Duun Rohde, Klaus Rostgaard, Michael Schwinn, Kari Stefansson, Hreinn Stefansson, Jacob Træholt, Unnur Þorsteinsdóttir, Henrik Ullum, Mie Topholm Bruun, Bitten Aagaard Jensen, Christina Mikkelsen, Christian Erikstrup, Ole Birger Pedersen, Erik Sørensen, Sisse Rye Ostrowski, Mie Topholm Bruun, Bitten Aagaard Jensen, Christina Mikkelsen, Hans-Jakob Hartling, Christian Erikstrup, Ole Birger Pedersen, Erik Sørensen, Morten Bagge Hansen, Sisse Rye Ostrowski

**Affiliations:** 1 Department of Clinical Immunology, Copenhagen University Hospital—Rigshospitalet, Copenhagen, Denmark; 2 The DANBIO Registry and the Danish Rheumatologic Biobank, Copenhagen Center for Arthritis Research (COPECARE), Centre for Rheumatology and Spine Diseases, Centre of Head and Orthopaedics, Copenhagen University Hospital—Rigshospitalet, Glostrup, Denmark; 3 Department of Clinical Medicine, Faculty of Health and Medical Sciences, University of Copenhagen, Copenhagen, Denmark; 4 Department of Oncology, Copenhagen University Hospital—Rigshospitalet, Copenhagen, Denmark; 5 Department of Clinical Biochemistry, Copenhagen University Hospital—Rigshospitalet, Copenhagen, Denmark; 6 Department of Respiratory Medicine, Copenhagen University Hospital—Bispebjerg, Copenhagen, Denmark; 7 Respiratory Medicine Section, Department of Medicine, Copenhagen University Hospital—Herlev and Gentofte, Hellerup, Denmark; 8 Section for Geogenetics, GLOBE Institute, Faculty of Health and Medical Sciences, Copenhagen University, Copenhagen, Denmark; 9 Institute of Biological Psychiatry, Mental Health Centre, Sct. Hans, Copenhagen University Hospital, Roskilde, Denmark; 10 Department of Cardiology, Copenhagen University Hospital—Rigshospitalet, Copenhagen, Denmark; 11 Novo Nordisk Foundation Center for Basic Metabolic Research, University of Copenhagen, Copenhagen, Denmark; 12 Clinical Immunology Research Unit, Department of Clinical Immunology, Odense University Hospital, Odense, Denmark; 13 Department of Clinical Immunology, Aalborg University Hospital, Aalborg, Denmark; 14 Department of Clinical Immunology, Aarhus University Hospital, Aarhus, Denmark; 15 Department of Clinical Medicine, Aarhus University, Aarhus, Denmark; 16 Department of Clinical Immunology, Zealand University Hospital, Køge, Denmark; 17 Novo Nordisk Foundation Center for Protein Research, Faculty of Health and Medical Sciences, University of Copenhagen, Copenhagen, Denmark; 18 deCODE genetics/Amgen Inc., Reykjavik, Iceland; 19 Danish Headache Center, Department of Neurology, Copenhagen University Hospital, Rigshospitalet—Glostrup, Copenhagen, Denmark; 20 Danish Cancer Society Research Center, Copenhagen, Denmark; 21 Department of Epidemiology Research, Statens Serum Institut, Copenhagen, Denmark; 22 Department of Health Science and Technology, Faculty of Medicine, Aalborg University, Aalborg, Denmark; 23 Faculty of Medicine, School of Health Sciences, University of Iceland, Reykjavik, Iceland; 24 Statens Serum Institut, Copenhagen, Denmark

**Keywords:** Cytokines, Autoimmunity, Genome-wide association studies, Type 1 diabetes, Cancer

## Abstract

Anti-cytokine autoantibodies are increasingly recognized as modulators of immune function and determinants of disease risk, yet their genetic basis and population-level impact remain unclear. Here we perform genome-wide association studies of autoantibodies against IL-1α, IL-6, IL-10, IFN-α, IFN-β, IFN-γ and GM-CSF in 15,000 individuals from the Danish Blood Donor Study. We identify 52 genome-wide significant loci, implicating genes enriched in antigen-presentation pathways. Using these data, we derive cytokine-specific polygenic risk scores and evaluate their associations across 300,000 individuals in the Copenhagen Hospital Biobank. Genetic predisposition to IL-1α autoantibodies is associated with reduced risk of rheumatoid arthritis and certain cancers, whereas genetic predisposition to IL-6 autoantibodies is associated with increased risk of diabetes, mirroring prior clinical observations. These findings define the genetic architecture of anti-cytokine autoantibodies and indicate their divergent effects on human disease risk at population scale, providing a framework for mechanistic investigation and potential clinical stratification.

## Abstract (web summary)

Despite limited understanding of cytokine autoantibodies, this study reveals a heterogeneous genetic architecture, with distinct profiles conferring reduced risk of certain cancers and rheumatoid arthritis, but increased susceptibility to diabetes.

## Introduction

Growing evidence underscores the clinical relevance of anti-cytokine autoantibodies (c-aAb), which represent a distinct and complex subset of autoimmune phenomena^1^. Cytokines are essential signaling molecules in the immune system, and functionally inhibitory c-aAb may exert broad immunomodulatory effects. These autoantibodies have been established as predictors for susceptibility to an expanding range of infectious pathogens. Notably, type I interferon (IFN) c-aAb have been strongly associated with COVID-19 disease severity^2,3^, and multiple publications have found an association between IL-6 c-aAb and opportunistic infections^4–6^. In 2017, the International Union of Immunological Societies classified multiple c-aAb as “phenocopies of primary immunodeficiencies,” underscoring their clinical relevance^7^.

In contrast, in the context of non-infectious autoimmune diseases, type I IFN c-aAb have been linked to a reduced incidence of type 1 diabetes^8^. Similarly, c-aAb targeting Interleukin (IL)-1α and IL-17 have been associated with improved outcomes in arthritis patients^9–11^, and recent findings show that neutralizing c-aAb against IL-33 correlates with enhanced lung function and decreased pro-inflammatory biomarkers in asthma patients^12^. These findings suggest that c-aAb-mediated immunomodulation is not inherently detrimental and, depending on the context, may be beneficial.

Importantly, high-titer c-aAb are also found in healthy individuals and can persist at stable levels for decades^9,13–17^. This persistence indicates that c-aAb are not merely transient responses to pathological insults but may be a common feature of immune variation.

Despite these insights, the full physiological impact and underlying mechanisms of c-aAb remain incompletely understood. Several studies have identified genetic factors influencing c-aAb development, implicating both central and peripheral tolerance mechanisms. For instance, mutations in the recombination-activating genes, which are essential for B- and T-cell receptor development, have been linked to cytokine-neutralizing c-aAb primarily targeting type I IFNs^18^. Likewise, the mentioned diabetes-protective IFN c-aAb have been associated with defects in the autoimmune regulator (AIRE) gene^15,19,20^. At the peripheral tolerance level, specific human leukocyte antigen (HLA) variants have been correlated with IFNγ c-aAb in patients with mycobacterial infections^21,22^.

To further investigate the genetic basis of c-aAb, we utilized c-aAb screening data from a well-characterized genotyped cohort of Danish Blood Donors^23^. Our aim was to identify genetic predictors of c-aAb and assess the association of said predictors with common diseases in genotyped patient cohorts.

**Fig. 1:**
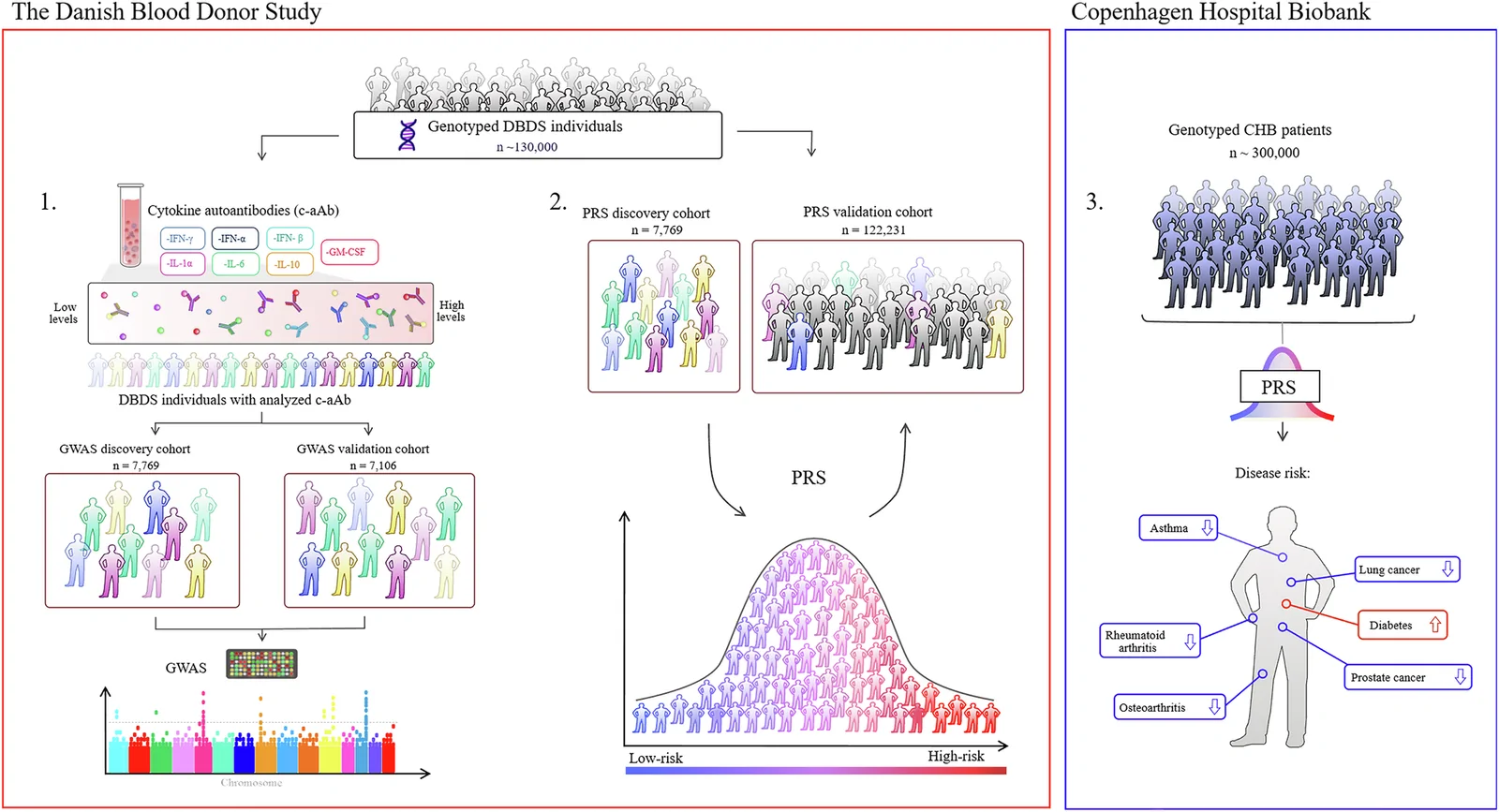
Study overview. A simplified summary of the main analyses performed. Step 1: genome-wide association study (GWAS) analyses for IL-1α, IL-6, IL-10, IFNα, IFNβ, IFNγ, and GM-CSF anti-cytokine autoantibodies (c-aAb) in the Danish Blood Donor Study (DBDS) cohort. These form the basis of functional annotation studies and Step 2: PRS calculation for c-aAb, based on the DBDS cohort. Statistically significant c-aAb polygenic risk scores (PRSs) were utilized in Step 3: testing of c-aAb PRSs as predictors of risk of common diseases in the Copenhagen Hospital Biobank (CHB) cohort.

We conducted a genome-wide association study (GWAS) c-aAb targeting seven cytokines in a cohort of *N* = 7769 deeply characterized healthy participants from the Danish Blood Donor Study (DBDS, *N* > 170,000, of which 130,000 have been genotyped)^23^ and validated the findings in a previously c-aAb-screened DBDS cohort (*N* = 7106) (Fig. 1). Independent single genetic variants were then used to construct polygenic risk scores (PRS).

In addition to DBDS, we also direct Copenhagen Hospital Biobank (CHB)^24^, a large-scale patient cohort (*N* ~ 500,000, of which *N* ~ 300,000 individuals have been genotyped), allowing for research in a wide range of diseases. We leveraged these resources to investigate the clinical impact of our identified genetic predictors of c-aAb by testing for the association of the c-aAb-PRSs derived from DBDS with 14 immunologically relevant and common diseases within the CHB cohort.

Our analyses revealed the genetic architecture of the investigated c-aAb, identifying several genetic variants associated with c-aAb, particularly within the major histocompatibility complex (MHC) region. Furthermore, we found significant associations between c-aAb-derived PRSs and several common diseases. These findings highlight the potential of c-aAb as immunomodulators and open further avenues for research into their physiological and clinical significance.

## Results

### High-titer C-aAb screening in healthy Danish Blood Donors

**Fig. 2:**
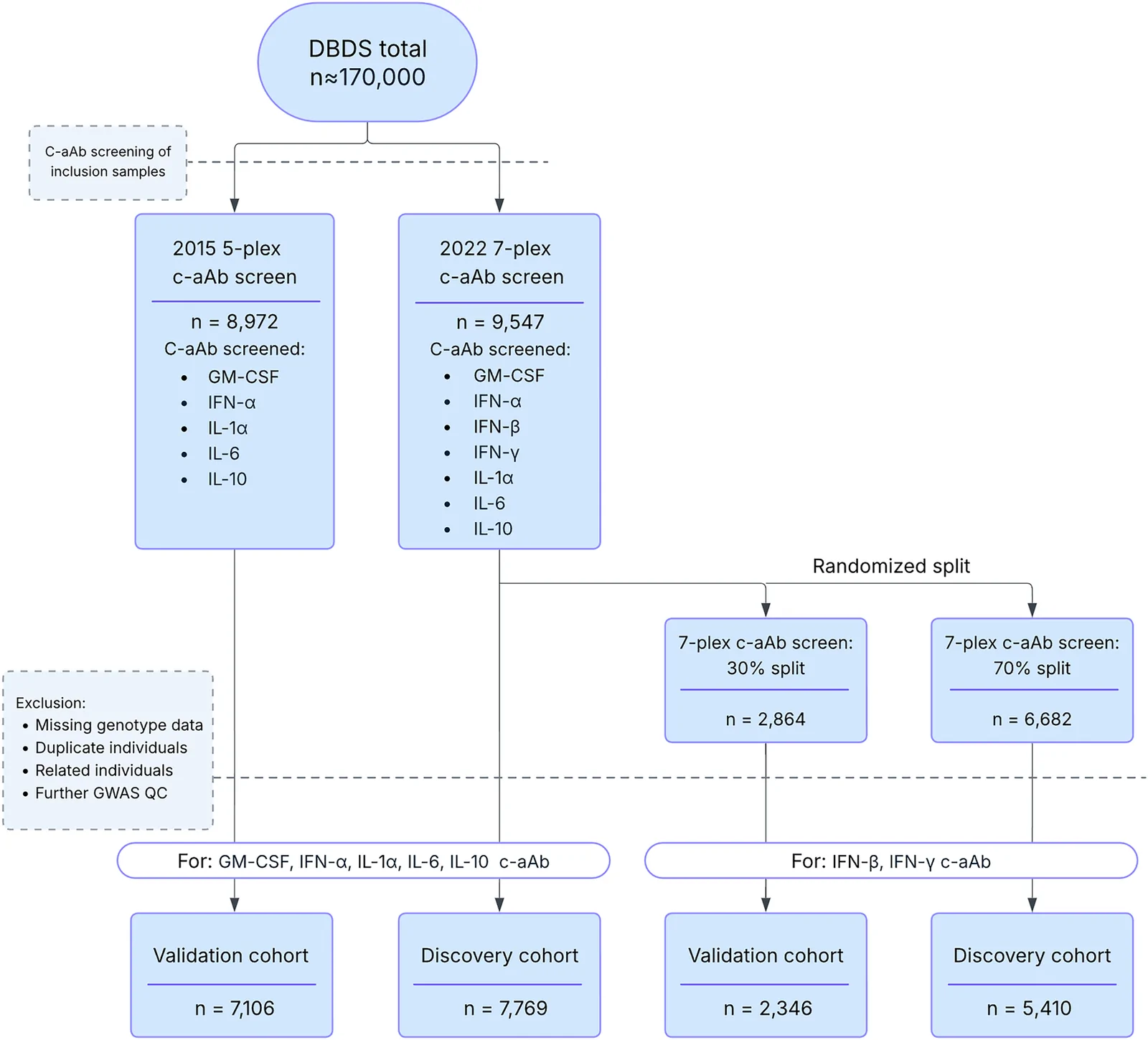
Flow chart of anti-cytokine autoantibodies (c-aAb) genome-wide association study (GWAS) discovery and validation cohort generation. Two c-aAb screenings were performed on subsets of the main Danish Blood Donor Study (DBDS) cohort: a 5-plex screen for c-aAb targeting IL-1α, IL-6, IL-10, GM-CSF, and IFNα in 2015, and a 7-plex screen in 2022, which also included IFNβ and IFNγ c-aAb. Following exclusion steps (as detailed in “Methods”), the 7-plex and 5-plex screened cohorts were used as discovery and validation cohorts for IL-1α, IL-6, IL-10, GM-CSF, and IFNα c-aAb. Discovery and validation cohorts for IFNβ and IFNγ c-aAb were generated by a 70–30% split of the 7-plex screened cohort.

To investigate the genetic variation underlying naturally occurring c-aAb, we drafted discovery and validation cohorts from c-aAb-screened and genotyped participants of DBDS, for the purpose of conducting GWAS analyses. The discovery cohort included individuals enrolled between 2010 and 2020 (*N* = 9547), with measured plasma levels of c-aAb targeting IL-1α, IL-6, IL-10, IFNα, IFNβ, IFNγ, and granulocyte-macrophage colony-stimulating factor (GM-CSF) at the time of enrollment (Fig. 2).

Following quality control (QC), a final sample of *N* = 7769 individuals remained for GWAS analyses targeting IL-1α, IL-6, IL-10, GM-CSF, and IFNα-specific c-aAb. For these c-aAb, an independent dataset comprising earlier c-aAb screenings from unrelated DBDS participants (*N* = 7106) was used for validation. For IFNβ and IFNγ-specific c-aAb, which did not have a corresponding existing c-aAb dataset, we used a 70%/30% random split of screened individuals to form discovery (*N* = 5410) and validation (*N* = 2346) cohorts. Age, sex, and c-aAb distribution for these sets of discovery/validation cohorts are shown in Supplementary Tables 1 and 2.

To evaluate the temporal stability of measured c-aAb levels, we analyzed longitudinal samples from a subset of 5-plex–screened participants: *N* = 5 × 12 high-titer cases and *N* = 10 controls. Cases were defined by median fluorescence intensity (MFI) above the 90th percentile for a given c-aAb, whereas controls had MFI values below the 90th percentile for all five specificities. Samples spanned 3–10 years of donations. Across all c-aAb specificities, case samples consistently remained above the high-titer threshold, while control samples remained below it throughout the entire sampling period (Supplementary Fig. 1).

### C-aAb GWAS and heritability

Based on the previously defined discovery and validation cohorts, we performed GWAS analysis of IL-1α, IL-6, IL-10, IFNα, IFNβ, IFNγ, and GM-CSF-specific c-aAb using age at c-aAb sample date, age squared, the first 10 genetic principal components (PC), and sex as covariates.

**Fig. 3:**
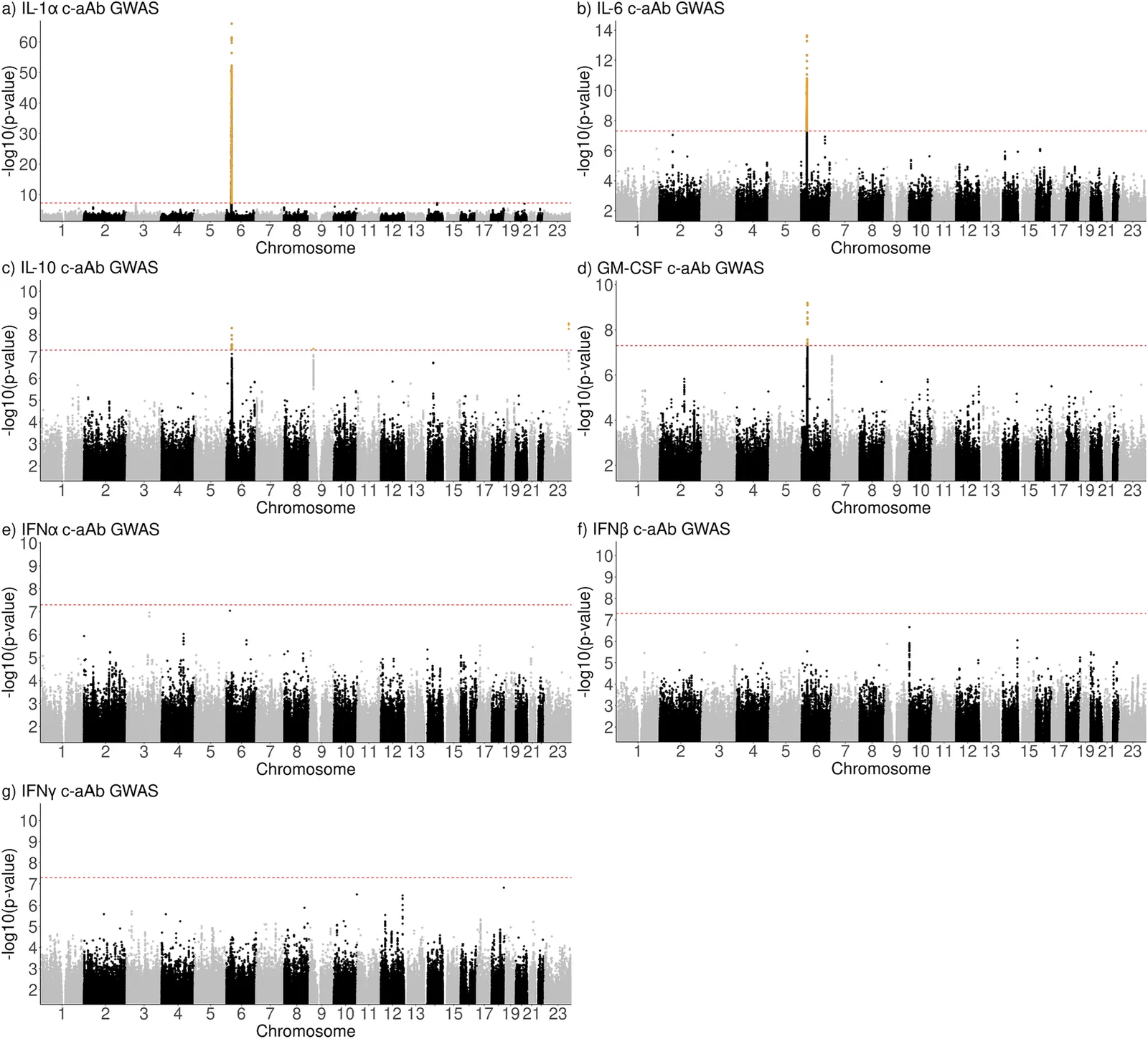
Manhattan plots for anti-cytokine autoantibodies (c-aAb) genome-wide association studies (GWASs). Analyses were conducted on inverse rank-transformed levels of c-aAb targeting **a** IL-1α, **b** IL-6, **c** IL-10, **d** GM-CSF, **e** IFNα, **f** IFNβ, and **g** IFNγ using generalized linear mixed models. The analyses were performed on a cohort of healthy, unrelated Danish Blood Donor Study (DBDS) participants of Northern European ancestry (*N* = 7769 for IL-1α, IL-6, IL-10, GM-CSF, and IFNα c-aAb; *N* = 5410 for IFNβ and IFNγ c-aAb). Age at sample date, age squared, sex, and the first 10 principal components were included as covariates in the analysis. Genome-wide significant variants (*p* < 5 × 10^−8^) are highlighted with orange.

Using the online functional mapping and annotation (FUMA) platform, we identified 52 genome-wide significant (*p* < 5 × 10^−^^8^) lead variants (0.1 <*r*^2^ < 0.6) associated with c-aAb targeting IL-1α, IL-6, IL-10, and GM-CSF. No genome-wide significant associations were detected for IFNα, β, or γ-specific c-aAb (Fig. 3). We used linkage disequilibrium (LD) score regression, excluding the MHC region, to calculate the following heritability (observed scale) estimates, with standard errors (SE): *h*^2^ = 0.1472 (SE = 0.086) for IL-1α c-aAb, *h*^2^ = 0.0695 (SE = 0.099) for IL-6 c-aAb, *h*^2^ = 0.1851 (SE = 0.1400) for IL-10 c-aAb, *h*^2^ = 0.0199 (SE = 0.1068) for GM-CSF c-aAb, *h*^2^ = 0.0302 (SE = 0.0925) for IFNα c-aAb, *h*^2^ = 0.3049 (SE = 0.1743) for IFNβ c-aAb and *h*^2^ = 0.0300 (SE = 0.1566) for IFNγ c-aAb. In all cases, the estimated heritability 95% confidence intervals (CI), defined as *h*^2^ ± 1.96 × SE, overlapped zero.

**Fig. 4:**
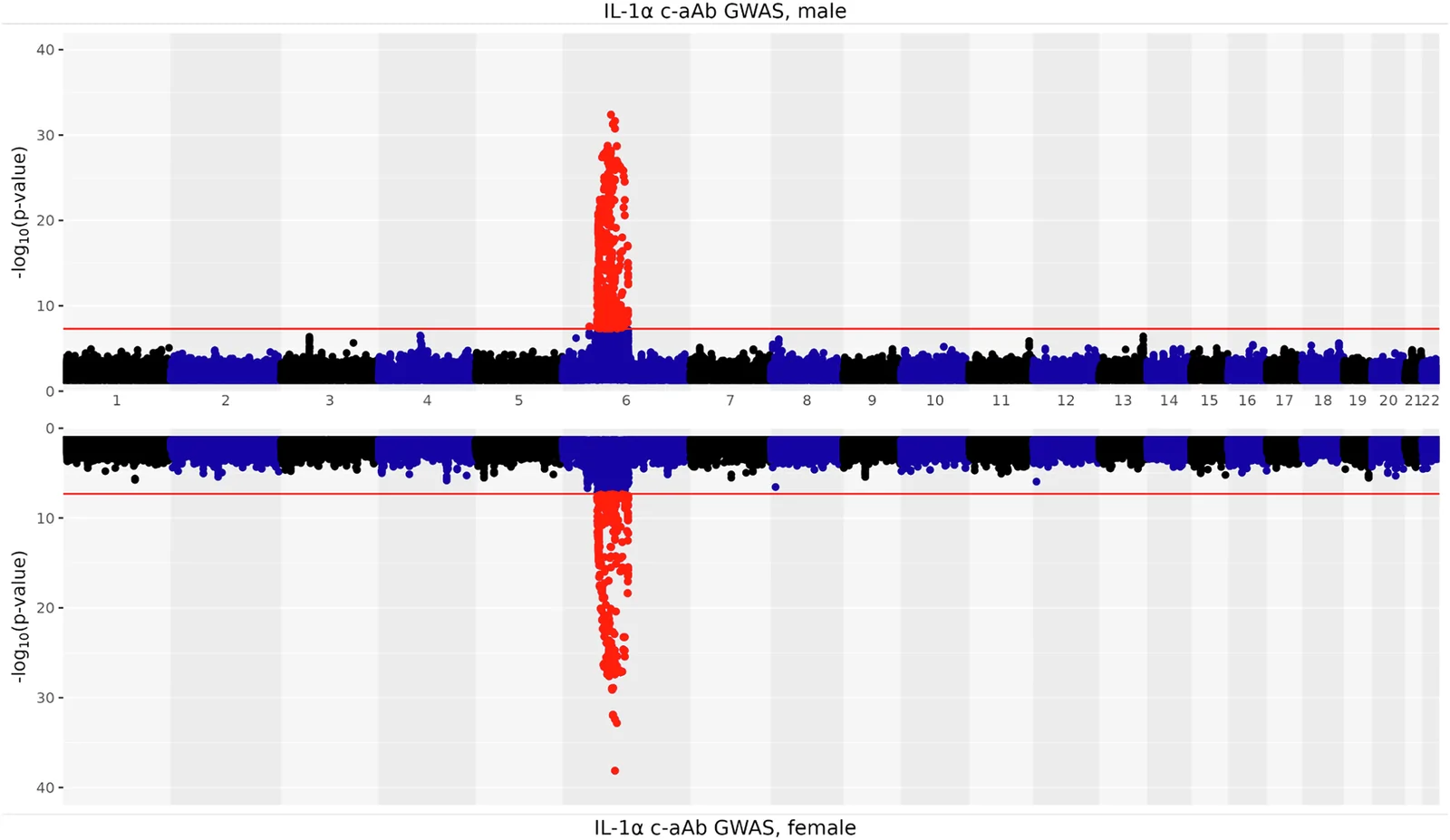
Miami plot for IL-1α anti-cytokine autoantibodies (c-aAb) sex-specific genome-wide association study (GWAS). Analyses were conducted on inverse rank-transformed IL-1α c-aAb using generalized linear mixed models in a cohort of exclusively male (*N* = 3939) or female (*N* = 4066) healthy, unrelated Danish Blood Donor Study (DBDS) participants of Northern European ancestry. Age at sample date, age squared, and the first 10 principal components were included as covariates. Genome-wide significant variants (*p* < 5 × 10^−8^) are highlighted in red.

Prior observations of c-aAb-sex associations^16^ led us to perform sex-stratified GWASs for each of the investigated c-aAb. Genome-wide significant independent variants were detected for both males and females for IL-1α c-aAb (Fig. 4), only in males for IL-6 c-aAb, and only in females for GM-CSF c-aAb (Supplementary Figs. 2 and 3). Unless otherwise specified, references to “c-aAb-associated variants/genes” hereafter refer to findings from the full-cohort GWAS.

### C-aAb-associated genomic loci and independent variants

Our GWAS analyses identified multiple independent variants, most of which mapped to a genomic locus within the MHC region on chromosome 6 (Table 1).

**Table 1:** Summary results of FUMA-based c-aAb GWAS analysis

		Variants (*N*)		Exonic variants (*N*)		CADD >12.37 Variants (*N*)		Mapped genes (*N*)		Loci
C-aAb	Cohort	Independent	Lead	Independent	Lead	Independent	Lead	Positional	eQTL	(*N*)
IL-1α	All	286	40	17	3	26	5	279	650	1
	Male	221	36	15	3	24	4	233	563	1
	Female	191	15	7	1	5	2	139	314	1
IL-6	All	79	12	0	0	6	0	58	368	1
	Male	12	2	0	0	0	0	20	186	1
IL-10	All	3	2	0	0	0	0	71	92	2
GM-CSF	All	2	1	0	0	0	0	14	86	1

Overview of the number of variants and mapped genes for the performed GWAS analyses. Only analyses yielding at least 1 genome-wide significant variant are listed. CADD score: an approximation of variant deleteriousness, as defined by Kircher et al.^60^, computed by the FUMA platform^40^. A CADD score >12.37 is considered pathogenic. Independent variant: genome-wide significant (*p* < 5 × 10^−8^) variants identified as having low LD to other variants (*r*^2^ < 0.6). Lead variant: the most likely variants associated with the c-aAb phenotype among identified independent variants, as determined by FUMA. Positionally mapped genes: genes containing a genome-wide significant variant within 10 kb of their genomic location, including regulatory regions. eQTL mapped genes: genes identified based on expression quantitative trait locus (eQTL) associations, representing known associations between variants and gene expression data from existing reference datasets.

Among all c-aAb analyzed, IL-1α c-aAb yielded the highest number of lead and independent variants as well as the strongest statistical associations (Table 1). Full-cohort GWAS was followed by FUMA processing, identifying 286 genome-wide significant independent variants (*r*^2^ < 0.6), including 40 lead variants.

All 286 independent variants mapped to the chromosome 6 MHC region (hg19 position: 29,811,624–33,081,265; lowest variant *p* = 1.009 × 10^−^^66^, *β* = 0.440, rs13207893). Among the 40 lead variants, three were exonic, and five had Combined Annotation Dependent Depletion (CADD) scores > 12.37, suggesting a higher likelihood of functional deleteriousness. Notably, both the exonic variants and those with high CADD scores were exclusive to the IL-1α c-aAb GWAS.

Replication in the independent validation cohort confirmed 34 of the 40 lead variants (Supplementary Data 1). The most statistically significant lead variants, rs13207893, rs9269964 (*p* = 3.871 × 10^−^^57^, *β* = 0.408), rs6911114 (*p* = 1.823 × 10^−51^, *β* = 0.493), and rs551560868 (*p* = 2.353 × 10^−51^, *β* = 0.500), were located near HLA class II genes *HLA-DQA1* and *HLA-DRB1*. Additional lead variants were proximal to *HLA-DRB5*, *HLA-DRB6*, *HLA-DQB1*, *HLA-DQB2*, and *HLA-E*.

Other nearby genes of interest included *C6ORF25* (chromosome 6 open reading frame 25, immunoglobulin-related), *PSMB9* (proteasome subunit beta type-9, immunoproteasome component), and *TAP2* (antigen peptide transporter 2). Among the 26 independent variants with CADD > 12.37, 17 were closest to *HLA-DQ, HLA-DRB*, or *HLA-E* genes, including three exonic, non-synonymous mutations (Supplementary Data 2).

FUMA analysis of the IL-6 c-aAb GWAS identified 79 independent variants and 12 lead variants, 8 of which were replicated in the validation cohort. All lead variants mapped to the chromosome 6 MHC genomic region (hg19 position: 32,208,882–33,081,265; lowest variant *p* = 2.300 × 10^−^^14^, *β* = 0.123, rs4959105). The nearest genes to these lead variants included *HLA-DRB1*, *HLA-DQA1*, and *C6ORF10* (Supplementary Data 3), which were also the closest genes to independent variants with CADD scores > 12.37 (Supplementary Data 4). 3 lead variants overlapped with those found in the full-cohort IL-1α c-aAb GWAS (rs557177282, rs201958431, and rs536229990), which all had *HLA-DQA1* as the nearest gene. No other overlaps of lead variants were observed between c-aAb GWASs.

For the IL-10 c-aAb GWAS, FUMA revealed 3 independent and 2 lead variants, located in two autosomal loci: one again in the chromosome 6 MHC region (hg19 position: 30,335,350–30,701,532; lowest variant *p* = 4.888 × 10^−^^9^, *β* = − 0.140, rs1264543) and the other on chromosome 9 (hg19 position: 21,191,374–21,417,681; lowest variant *p* = 4.378 × 10^−^^8^, *β* = − 0.230, rs182975434). The genes nearest to these lead variants were *MICC* (MHC class I polypeptide-related sequence C) and *IFNA10* (Interferon alpha-10) (Supplementary Data 5-6).

The GM-CSF c-aAb GWAS yielded 2 independent variants, including 1 lead variant in the chromosome 6 MHC region (hg19 position: 32,592,476–32,678,206; lowest variant *p* = 5.171 × 10^−10^, *β* = − 0.112, rs281862690). The nearest gene to this locus was *HLA-DQB1* (Supplementary Data 7 and 8).

### Multi-trait C-aAb GWAS analysis

The presence of a shared genetic architecture across our investigated c-aAb phenotypes was tested through multi-trait GWAS, performed pairwise for all combinations of c-aAb GWAS summary statistics. Three hundred fifty-seven significant phenotype–region associations were identified with joint test *p* values below our significance threshold (*p* < 2.9 × 10^−^^5^; Bonferroni correction for 1703 tested genomic regions). To interpret these associations, we categorized them as “shared,” “opposite-direction effects,” or “trait dominated” for the specific c-aAb pairing, based on univariate association strength and concordance of regional *z*-score directionality.

Across all comparisons, seven associations in five genomic regions (655, 656, 657, 658, and 1450) suggested shared genetic effects, involving three c-aAb pairings (IL-1α × GM-CSF, IL-1α × IL-6, and IL-1α × IL-10 c-aAb). These regions showed joint significance together with univariate association evidence (*p* < 1 × 10^−^^5^) for both phenotypes and consistent *z*-score directionality. For instance, region 655 (chromosome 6, position 29,737,971–30,798,168) demonstrated a joint association with IL-1α and GM-CSF c-aAb (*p* = 4.404 × 10^−^^8^), supported by univariate evidence for both c-aAb and concordant, positive *z*-scores (*p* = 7.175 × 10^−8^*, z* = 4.504, for IL-1α c-aAb; *p* = 1.818 × 10^−7^, *z* = 5.216 for GM-CSF c-aAb).

We also identified four associations suggesting opposite-direction pleiotropy, in which both phenotypes showed non-null univariate association but with opposing regional *z*-score signs. These results corresponded to three genomic regions (655, 656, and 657) and three c-aAb pairings (IL-1α c-aAb × GM-CSF and IL-10 c-aAb, and IL-6 c-aAb × GM-CSF c-aAb).

A total of 180 associations were deemed trait-dominated, and 166 were uncategorized. Full results are provided in Supplementary Data 9.

### C-aAb causal candidate gene prioritization

To further identify potential causal genes associated with IL-1α, IL-6, IL-10, and GM-CSF c-aAb phenotypes, we employed FUMA’s expression quantitative trait locus (eQTL)-based gene mapping and performed gene-level association testing using the Multi-marker Analysis of GenoMic Annotation (MAGMA) tool.

For the IL-1α c-aAb GWAS summary statistics, FUMA identified 279 genes through positional mapping and 650 genes via eQTL mapping, resulting in a combined total of 704 candidate genes (Table 1 and Supplementary Data 1,0). The MAGMA gene-based test further identified 81 unique, statistically significant protein-coding genes based on aggregated IL-1α c-aAb-associated variants (Supplementary Data 1,1).

Following the approach of Burns et al.^25^, we constructed a prioritized list of putative causal genes for downstream GENE2FUNC gene-set enrichment analysis. Genes were selected if they met at least two of the following criteria: (i) proximity to lead GWAS variants, (ii) identification through eQTL mapping, and (iii) statistical significance in the MAGMA gene-based test. This process yielded a list of 91 unique candidate genes associated with IL-1α c-aAb (Supplementary Data 1,2). Notably, genes fulfilling all three criteria included *HLA-E*, HLA class II genes *HLA-B*, -*DOB*, -*DQA1*, -*DQB1-2*, -*DRB1,* and *HLA*-*DRB5-6*, as well as *C6ORF25*, *PSMB9*, and *TAP2*.

For IL-6 c-aAb, FUMA identified 368 genes through eQTL mapping (Supplementary Data 1,3) and 5 additional genes through the MAGMA gene-based test (Supplementary Data 1,4), resulting in a total of 6 prioritized candidate genes. These included *HLA-DQA1*, *-DQB1, -DRA* and *HLA-DRB1* (Supplementary Data 1,5).

In the case of IL-10 c-aAb, 92 genes were mapped via eQTL associations (Supplementary Data 1,6), and 10 genes were identified through MAGMA (Supplementary Data 17). From these, six candidate genes were prioritized, including *HLA-E* and *IFNA10* (Supplementary Data 18).

For GM-CSF c-aAb, 86 genes were mapped through eQTL associations (Supplementary Data 19); however, only one gene—*HLA-DQB1*—fulfilled the candidate criteria (Supplementary Data 20).

### Tissue/pathway gene-set enrichment analysis

We conducted gene set enrichment (GSE) analysis using a list of prioritized candidate causal genes for c-aAb, employing the default, comprehensive set of gene sets in the FUMA GENE2FUNC tool. This analysis revealed 386 statistically significant gene sets (*p* < 0.05) associated with IL-1α c-aAb. Focusing on Gene Ontology (GO) terms and curated gene sets relevant to c-aAb causality, the most significantly enriched gene sets were linked to adaptive immunity, antigen processing, and antigen presentation (Supplementary Data 21).

Significant enriched gene sets also represented a wide range of diseases, with the most statistically significant being the GWAS catalog gene sets of “autism spectrum disorder or schizophrenia” (*p* = 1.213 × 10^−95^), “ulcerative colitis” (*p* = 2.123 × 10^−77^), and “asthma” (*p* = 2.777 × 10^−65^). Differentially expressed genes (DEGs) were observed across tissues, such as lung, heart, brain, liver, muscle, and spleen, as well as upregulated DEGs in blood. To further explore tissue enrichment, we performed a conditional analysis on IL-1α c-aAb candidate genes, excluding enriched genes shared by the tissue “Brain_Anterior_cingulate_cortex_BA24,” the most statistically significant tissue in the initial IL-1α c-aAb enrichment analysis (*p* = 3.579 × 10^−8^). No tissues were identified as significantly enriched in the conditional analysis, suggesting a large degree of shared enriched genes across tissues.

A MAGMA gene set enrichment analysis was also performed for the IL-1α c-aAb GWAS. This analysis, which integrates MAGMA results with select gene sets while accounting for variant *p* values, identified two enriched gene sets: “GOBP_PROPRIOCEPTION” (4 genes, *p* = 0.004, *β* = 2.969, 95% CI [1.820–4.118]) and “GOMF_PEPTIDE_ANTIGEN_BINDING” (28 genes, *p* = 0.045, *β* = 0.779, 95% CI [0.443–1.115]). Additionally, MAGMA tissue expression analysis using the 54 tissues of the Genotype-Tissue Expression project (GTEx) v8^26^ and a one-sided linear regression model, identified a positive association between gene expression in whole blood and gene-level GWAS associations (*p* = 3.573 × 10^−5^, *β* = 0.023, 95% CI [0.012–0.034]).

Significant gene-set enrichment was also observed for IL-10 c-aAb candidate genes, including three gene sets: “KEGG_ANTIGEN_PROCESSING_AND_PRESENTATION,” “KEGG_AUTOIMMUNE_THYROID_DISEASE,” and “KEGG_NATURAL_KILLER_CELL_MEDIATED_CYTOTOXICITY” (Supplementary Data 22). Gene-set enrichment was not observed for IL-6 or GM-CSF c-aAb candidate genes.

We also performed tissue enrichment for non-prioritized genes from our c-aAb GWAS summary statistics through stratified LD score regression (S-LDSC), based on the tissue groupings designated by S-LDSC package developers Finucane et al.^27^. The “immune/blood” category was found to be nominally significantly enriched (*p* < 0.05) for IL-1α, IL-10, GM-CSF and IFNβ c-aAb in unadjusted analyses, but no significance remained following adjustment for multiple testing.

### Sex-specific C-aAb GWAS differences

When comparing female and male-specific GWAS results for IL-1α c-aAb, the female cohort exhibited fewer independent and lead variants than the male (191/15 vs. 221/36, see Table 1). The overlap of variants (rsID) between the analyses increased as the stringency of variant filtering was relaxed. For example, 40% of the lead variants from the female-specific GWAS were shared with the male-specific, as well as 51% of independent variants, and 85% of genome-wide significant variants (561 significant variants for females, 1658 for males).

We applied the same gene prioritization and enrichment analyses to both the male and female-specific GWAS data as before, identifying 46 candidate genes for the female-specific GWAS and 72 for the male-specific GWAS. Forty-three candidate genes were shared between the sexes (Supplementary Data 23), corresponding to 95% and 60% of candidate genes from the female and male-specific GWASs, respectively. Genes exclusive to the female-specific GWAS included *HLA-DMB*, *HLA-DQB1-AS1,* and *XXbac-BPG254F23.6*, with *HLA-DRB6*, *HLA-DRB9* and *BTNL2* exclusive to the male-specific GWAS, among others (Supplementary Data 24).

To identify variants with sex-dependent effects, we ran a full-cohort GWAS with REGENIE’s --interaction option, testing the “variant × sex” term as a predictor of c-aAb.

For IL-10 c-aAb, two variants on chromosome 1 showed significant variant × sex interactions, with both demonstrating stronger negative effects in males than females (rs74742063, position 205579201, *p* = 4.866 × 10^−8^, *β* = − 0.234, 95% CI [−0.3192 to −0.1507] and rs75541573, position 205579491, *p* = 2.891 × 10^−8^, *β* = − 0.238, 95% CI [−0.3226 to −0.1540]). Both variants were within intronic regions, with *MFSD4* (major facilitator superfamily domain containing 4A) as the nearest gene.

For IFNβ c-aAb, one intergenic variant on chromosome 12 showed a positive effect in males compared to females (chromosome 12, position 97239764, *p* = 6.75 × 10^−10^, *β* = 0.2308, 95% CI [0.1576–0.3039]). Nearest genes were *NEDD1* (neural precursor cell expressed, developmentally down-regulated 1) and *RMST* (Rhabdomyosarcoma 2-associated transcript). For IL-1α, IL-6, GM-CSF, IFNα, and IFNγ c-aAb, no variant × sex interactions reached genome-wide significance.

### C-aAb GWAS polygenic risk score development

To explore the potential of c-aAb genetics as disease risk markers, we utilized the PRSice-2 tool to estimate PRSs based on our c-aAb GWAS summary statistics. After adjustments for overfitting and multiple testing, we obtained statistically significant PRSs for IL-1α, IL-6, and IL-10 c-aAb, as well as for both sex-specific IL-1α c-aAb GWAS data (Table 2). The highest explained variance (*r*^2^) was observed for the full-cohort IL-1α c-aAb PRS (PRS model *p* = 2.51 × 10^−34^, *β* = 8.55, SE = 0.69), which accounted for 2.49% of the variance. Comparable levels of variance explained were found for the male (*r*^2^ = 2.45%, *p* = 1.75 × 10^−18^, *β* = 4.85, SE = 0.55) and female-specific PRSs (*r*^2^ = 2.25%, *p* = 6.12 × 10^−16^, *β* = 4.54, SE = 0.56). The IL-6 (*p* = 2.60 × 10^−8^, *β* = 3.42, SE = 0.61) and IL-10 c-aAb PRSs (*p* = 1.14 × 10^−5^, *β* = 2.55, SE = 0.58) explained 0.5% and 0.3% of the variance, respectively. All PRSs demonstrated a positive association with their respective c-aAb phenotypes across PRS quantiles (Supplementary Figs. 8–10).

**Table 2:** C-aAb PRS overview

C-aAb	Cohort	*p*	Threshold	Variants (*N*)	*β*	SE	*r*^*2*^, PRS	*r*^*2*^, base model	*r*^*2*^, full model
IL-1α	All	2.51 × 10 ^−34^	5 × 10^−8^	38	8.55	0.69	0.0249	0.0004	0.0290
	Male	1.75 × 10^−18^	5 × 10^−8^	17	4.85	0.55	0.0245	0.0005	0.0251
	Female	6.12 × 10^−16^	5 × 10^−8^	12	4.54	0.56	0.0225	0.0021	0.0245
IL-6	All	2.599 × 10^−8^	5 × 10^−8^	7	3.42	0.61	0.0051	0.0026	0.0078
IL-10	All	1.14 × 10^−5^	5 × 10^−8^	2	2.55	0.58	0.0030	0.0199	0.020

Overview of PRS development for c-aAb with genome-wide significant variants. PRSs were developed with PRSice-2 using linear regressions with two-sided Wald tests for determining PRS-c-aAb associations. Empirical *p *values were computed to account for multiple comparisons across PRS thresholds. All reported PRSs had empirical *p* < 0.05.

Threshold: maximum GWAS variant *p* value for inclusion in best model fit PRS (largest *r*^2^).

*p*: *p* value of PRS model.

Variants: number of variants with *p* below threshold included in PRS from corresponding GWAS.

*β*: coefficient for PRS association to inverse log-rank transformed c-aAb.

SE: standard error.

*r*^*2*^, PRS: variance explained by the PRS itself.

*r*^*2*^, base model: variance explained by baseline covariates (age at sample date and sex, if applicable).

*r*^*2*^, full model: variance explained by the full model (PRS, age at sample date and sex, if applicable).

To facilitate interpretation, we defined binary “high/low” c-aAb titer variables as phenotypes for further full-cohort GWAS analyses, followed by PRS development for the high/low c-aAb titer phenotypes. “High titers” were defined as c-aAb MFI >90th percentile, and “low titers” as MFI <75th percentile. A statistically significant PRS (*p* = 8.98 × 10^−42^, *β* = 36.25, SE = 2.68) for high/low c-aAb was achieved exclusively for IL-1α c-aAb, with *r*^2^ for the PRS = 7% and *r*^2^ for the full model (PRS + covariates) = 11.4%. Variants and associated genes included in statistically significant PRS’s are listed in Supplementary Data 25–30.

### C-aAb PRSs as predictors of diseases

We tested the developed c-aAb PRSs for their ability to predict time of diagnosis for 14 common diseases in five sub-cohorts of Copenhagen Hospital Biobank patients (CHB, Supplementary Tables 3–7). PRS was used as a predictor of time to disease diagnosis, with Cox survival analyses adjusting for age as the underlying time scale and sex as a covariate. Following multiple testing correction, the IL-1α and IL-6 c-aAb PRSs predicted significantly altered hazard ratios (HR) for several diseases.

**Fig. 5:**
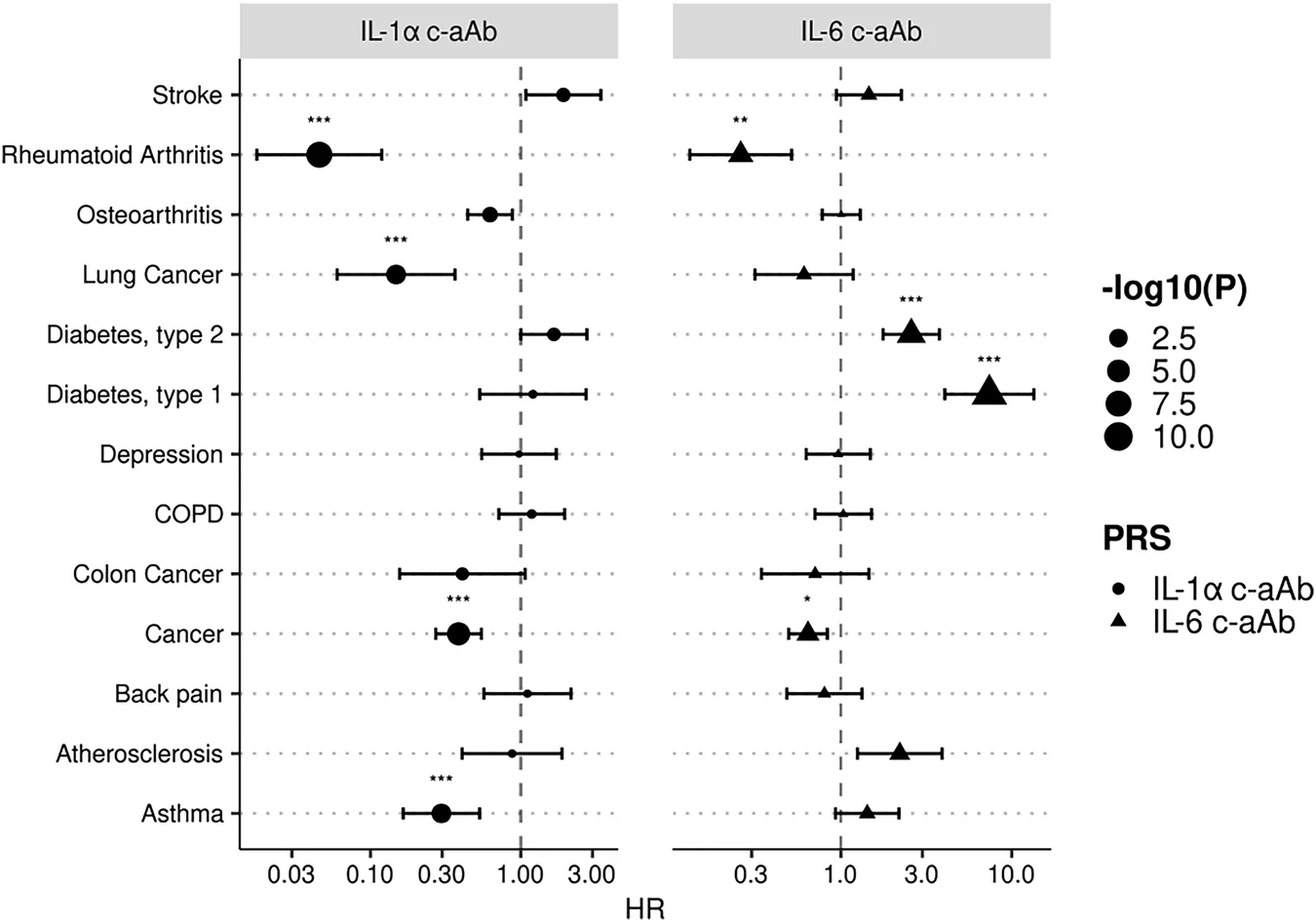
Anti-cytokine autoantibodies (c-aAb), polygenic risk scores (PRSs) vs. risk of common diseases. Data are presented as hazard ratios (HR) for time to disease diagnosis with 95% confidence intervals (CI), derived from two-sided Cox proportional hazards regression analyses. Tests were performed in Copenhagen Hospital Biobank (CHB) sub-cohorts according to diagnosis: the CHB-study on degenerative and episodic brain disorders (atherosclerosis and stroke, *N* = 237,444); the CHB-study on cancer using the Danish Cancer biobank (cancer, colon cancer and lung cancer *N* = 148,783); the CHB-study on chronic inflammatory diseases (asthma, COPD, and rheumatoid arthritis, *N* = 215,512); the CHB-study on pain and degenerative musculoskeletal diseases (back pain, depression, and osteoarthritis, *N* = 203,320); and the CHB-study on oral-cardiometabolic health (diabetes, *N* = 242,599). Survival time was defined as the time from entry in the patient registry to diagnosis, with age as the underlying time scale. Standard deviation (SD)-adjusted PRSs developed from genome-wide association studies (GWASs) of IL-1α and IL-6 c-aAb were used as predictors, with sex as a covariate. Circles denote IL-1α c-aAb- derived PRS, triangles IL-6 c-aAb derived PRS. *P* values were adjusted for multiple comparisons. * = *p* < 0.05, ** = *p* < 0.01, and *** = *p* < 0.001. Source data are provided as a Source Data file.

The IL-1α c-aAb PRS, based on the full-cohort GWAS, predicted a reduced risk of both chronic inflammatory diseases and cancer. An increase of 1 standard deviation (SD) in the PRS was associated with a 96% reduced risk of rheumatoid arthritis (*p* = 1.8  × 10^−8^, HR = 0.05, 95% CI [0.02–0.12]), as well as a reduced risk of asthma (*p* = 0.003, HR = 0.30, 95% CI [0.17–0.53]). The IL-1α c-aAb PRS was also associated with a reduced risk of osteoarthritis, though this result became borderline significant after multiple testing correction (*p* = 0.06, HR = 0.54, 95% CI [0.44–0.88]). The IL-1α c-aAb PRS was associated with reduced risk of lung cancer (*p* = 0.002, HR = 0.15, 95% CI [0.06–0.37]) and reduced risk of receiving any cancer diagnosis (*p* = 6.4 × 10^−6^, HR = 0.39, 95% CI [0.27–0.55], Fig. 5).

The IL-6 c-aAb PRS also predicted reduced risk of rheumatoid arthritis (*p* = 0.008, HR = 0.26 for 1 SD increase in PRS, 95% CI [0.13–0.51]), but an increased risk of both type 1 (*p* = 4.5 × 10^−9^, HR = 7.40, 95% CI [4.06–13.46]) and type 2 diabetes (*p* = 7.2 × 10^−5^, HR = 2.58, 95% CI [1.76–3.78]).

Sex-specific PRS-outcome analyses for IL-1α c-aAb revealed statistically significant reduced risks for diagnosis of asthma (*p* = 0.016, HR = 0.40, 95% CI [0.25–0.65]), rheumatoid arthritis (*p* = 9.74 × 10^−4^, HR = 0.15, 95% CI [0.06–0.34]), general cancer (*p* = 6.24 × 10^−7^, HR = 0.48, 95% CI [0.37–0.62]), and lung cancer (*p* = 8.40 × 10^−4^, HR = 0.22, 95% CI [0.11–0.43]) exclusively for the male-specific PRS, which was also associated with reduced risk of prostate cancer (*p* = 9.35 × 10^−4^, HR = 0.29, 95% CI [0.17–0.51]; Supplementary Fig. 11).

Further analysis of disease risks by PRS quantiles (0–20%, 20–40%, 40–60%, 60–80%, and 80–100%) revealed that reduced risks associated with IL-1α c-aAb PRS for asthma, osteoarthritis, rheumatoid arthritis and the various cancers were confined to the highest (5th) PRS quantile (Supplementary Fig. 12). In contrast, the increased diabetes risk associations for IL-6 c-aAb PRS were most pronounced in the 4th and 5th PRS quantiles (Supplementary Fig. 13).

We also stratified the CHB cohorts by birthdate quartiles to account for age-dependent confounding. For the IL-1α c-aAb PRS, the reduced risk of cancer diagnosis was observed in the earliest-born (1st/2nd quartiles) individuals, reduced asthma risk in the 2nd/3rd quartiles, and reduced rheumatoid arthritis risk across all but the most recently born (4th quartile) individuals (Supplementary Fig. 14). For the IL-6 c-aAb PRS, the increased diabetes risk was observed in all but the earliest-born individuals (Supplementary Fig. 15).

## Discussion

This study, conducted using two large-scale genotyped cohorts of healthy Danish Blood Donors and patients, represents the largest investigation to date of the genetic architecture of c-aAb. We identified genetic variants associated with c-aAb targeting IL-1α, IL-6, IL-10, and GM-CSF. We were also able to calculate PRSs for IL-1α and IL-6-specific c-aAb. Most lead variants localized to the MHC region, with several candidate genes encoding HLA class II molecules, including *HLA-DQA1*, *HLA-DQB1–2*, *HLA-DRA*, and *HLA-DRB1/5/6*. Gene-set enrichment analysis for IL-1α and IL-10 c-aAb candidate genes revealed strong enrichment of antigen presentation pathways. These findings align with previous reports linking HLA class II genetic variants to autoantibody generation^28,29^, including IFNγ c-aAb for *HLA-DQ* and *HLA-DR* variants in patients with mycobacterial infections^21^.

IL-1α c-aAb were associated with the largest number of lead variants, mapped genes, candidate genes, and enriched gene sets. We identified no significant genetic predictors of any of our investigated interferon-specific c-aAb. This may suggest a variable tolerance for c-aAb depending on cytokine specificity, as indicated by the differences in distribution of c-aAb targeting IL-1α compared to IFNα in our cohort of healthy individuals^16^.

In sex-stratified analyses, only IL-1α c-aAb displayed significant genetic predictors in both sexes. Despite the male predominance of IL-1α c-aAb, genetic associations largely overlapped between the male and female-specific analyses, and no genome-wide significant variant-sex interactions were identified, suggesting a limited impact of sex on the genetics of IL-1α c-aAb. However, we identified three significant variant-sex interactions for IL-10 and IFNβ c-aAb, despite the absence of significant variants in sex-stratified GWAS. This discrepancy could indicate true sex-specific genetic effects masked by reduced statistical power when analyzing sexes separately.

Estimates of SNP-based heritability outside the MHC region were imprecise, with 95% confidence intervals overlapping zero for all c-aAb investigated. This pattern suggests that, within the limits of the current sample size, detectable heritability is largely concentrated within the MHC. Multi-trait GWAS analyses identified several loci exhibiting shared effects and opposite-direction pleiotropy between individual comparisons of IL-1α, IL-6, IL-10, and GM-CSF c-aAb. However, most significant loci-c-aAb associations appeared to be carried by strong, specific c-aAb summary statistics results. Ultimately, our findings suggest that while some shared loci exist across specific c-aAb pairings, individual c-aAb may have distinct rather than shared genetic architectures. However, while the MHC contains the strongest detectable effects at our current sample size, additional non-MHC genetic contributors for certain c-aAb may be identified with increased statistical power.

Using various patient cohorts, c-aAb have been found associated to multiple infectious or autoimmune diseases. In prior works, we have established healthy Danish Blood Donors to carry functionally neutralizing c-aAb, both in vitro and in vivo, where c-aAb introduced through blood transfusion or IgG pools derived from Danish donors can inhibit recipient target cytokines^14,30–35^. In both this and prior works, we have also shown that high-titer c-aAb can remain stable for more than a decade in healthy blood donors, and that they are relatively prevalent within this population^13,14,16^.

These observations, and the broad immunological roles of cytokines, led us to conduct a broad investigation of c-aAb health implications in the Danish population. We calculated statistically significant PRSs for IL-1α, IL-6, and IL-10 c-aAb, which were tested for associations with time to diagnosis for 14 common diseases in the CHB cohort. The IL-1α c-aAb PRS predicted reduced risks for rheumatoid arthritis and asthma, consistent with the prior findings^9,10,12,36^. This finding is notable since it supports the notion that the PRS for IL-1α c-aAb is a proxy of the clinical findings for IL-1α c-aAb. Notably, IL-1α c-aAb PRS was also associated with reduced risks for lung and prostate cancer, as well as a general reduced cancer risk. The carcinogenesis of a persistently pro-inflammatory environment is well known, and the pro-inflammatory cytokines IL-1α and IL-1β have been implicated in cancer development^37^. All diseases with IL-1α c-aAb PRS-associated risk were represented in the IL-1α c-aAb GWAS candidate gene GSE analysis, with enriched gene sets for asthma, lung cancer, prostate cancer and joint damage in rheumatoid arthritis. Our findings indicate a potential role of IL-1α c-aAb in modulating disease risk, including cancer development.

IL-6 c-aAb PRS was associated with an increased risk of both type 1 and type 2 diabetes, suggesting a potential pathogenic role for IL-6 c-aAb in diabetes development, as previously observed in studies of the c-aAb^38^, again supporting the notion that the PRS and c-aAb were associated with similar disease risks.

The usage of DBDS as the basis for c-aAb PRS computations and GWAS analyses may limit the generalizability of the findings in patient cohorts due to the health-based selection criteria of DBDS, with blood donors potentially representing individuals uniquely tolerant of high-titer c-aAb. Conversely, DBDS may be lacking disease-susceptible individuals with fully cytokine-neutralizing c-aAb, and thereby specific c-aAb genotypes tied to adverse outcomes. The demographic composition of our DBDS cohort may also have influenced c-aAb distribution compared to other cohorts; for instance, IFNα c-aAb prevalence increases after age 70^39^, while DBDS eligibility is currently capped at age 70. Additionally, ancestry composition may influence c-aAb distribution—IFNγ c-aAb are enriched in individuals of South East Asian ancestry^21,22^, while our cohort was of North European descent. Such factors could explain the lack of genetic predictor findings for IFNα, β, and γ c-aAb.

The MHC region is often excluded from genetic analyses, including FUMA, due to its complex and polymorphic structure^40^. However, based on our autoantibody phenotype, as well as pre-existing c-aAb-linked HLA findings, we deemed it relevant to include. Indeed, the genetic variants associated with c-aAb were predominantly located in MHC-coding genes and enriched for gene sets involved in antigen processing, supporting existing literature.

Finally, our findings are associative, identifying putative—not definitive—causal genes. These limitations could be addressed through large-scale c-aAb screening in genotyped patient cohorts, especially those enriched for older individuals or specific ancestries. Targeted studies in cancer (for IL-1α c-aAb) and diabetes (for IL-6 c-aAb) patients may clarify physiological roles and therapeutic potentials.

Although environmental factors likely contribute significantly to the induction of c-aAb, our GWAS findings highlight genetic predictors for c-aAb targeting both pro- and anti-inflammatory cytokines and suggest a heterogeneous genetic architecture across c-aAb specificities. Our GWAS results enabled the calculation of c-aAb PRSs, which predicted altered risks of established c-aAb-linked diseases, such as rheumatoid arthritis and diabetes and highlights possible disease targets for future c-aAb research, such as cancer and asthma.

## Methods

### Ethical statement

For the DBDS cohort, genetic studies were approved by the National and Zealand Regional Committees on Health Research Ethics as well as the Capital Region Data Protection Office (NVK-1700407, SJ-470, and P-2019-99). All DBDS participants have given informed oral and written consent for the use of their biological and health data in research, including research on genetics and age-related diseases. For the CHB cohort, based on Danish law, samples have been genotyped under a specific project and ethical approvals. This includes the five CHB sub-cohorts used in this study: the CHB-study on degenerative and episodic brain disorders (CHB-DEBD), approved by the Capital Region Committee on Health Research Ethics (H-21058057) and the Capital Region Data Protection Office (P-2022-392); the study on Cancer using the Danish Cancer biobank (CHB-CDC), approved by the Capital Region Data Protection Agency (P-2022-393) and the National Scientific Committee on Health Research Ethics (NVK-2200966); the study on chronic inflammatory diseases (CHB-CID), approved by the Capital Region Committee on Health Research Ethics (H-22021178) and the Capital Region Data Protection Office (P-2022-876); the study on pain and degenerative musculoskeletal diseases (CHB-PDS), approved by the Capital Region Data Protection Office (P-2019-51) and the National Scientific Committee on Health Research Ethics (NVK-1803812); and the CHB-study on oral-cardiometabolic health (CHB-OCMS), approved by the Zealand Region Committee on Health Research Ethics and the Capital Region Data Protection Office (SJ-989 and P-2022-913). All CHB-participants were aged over 18 years at the time of inclusion and were informed about their participation in the genetic studies by electronic secure mail (E-boks). Participants who opted out of the cohort were excluded from all analyses.

No DBDS or CHB participants received compensation. The design and conduct of the study complied with all relevant regulations regarding the use of human study participants and adhered to the principles defined by the Declaration of Helsinki.

### Cohorts

#### DBDS

DBDS is a nationwide cohort initiated in 2010, which includes > 170,000 blood donors, of which approximately 130,000 have presently been genotyped. Upon inclusion, participants complete a health questionnaire, and a plasma sample is stored in the biobank, as well as plasma samples from each future donation. The DBDS is open to all active members of the Danish blood donor corps between the ages of 18 and 70 (67 until 2019)^41^. Individuals who withdrew their consent at some point after DBDS inclusion were excluded from all analyses.

In this study, the initial discovery cohort consisted of 9547 healthy DBDS participants enrolled between 2010 and 2020, randomly selected from the main DBDS cohort to ensure an age-and-sex-matched cohort^42^. Multiplex data for c-aAb against GM-CSF, IFNα, IFNβ, IFNγ, IL-1α, IL-6, and IL-10 were available for these individuals.

The validation cohort comprised DBDS participants enrolled exclusively in 2010 (*N* = 9774), who had been screened for c-aAb in an earlier study^16^. This cohort included only data for c-aAb against GM-CSF, IFNα, IL-1α, IL-6, and IL-10, as these constituted the c-aAb multiplex panel at the time of screening. To validate the results for IFNβ and IFNγ c-aAb, the main discovery cohort was randomly divided into two sub-cohorts (70% discovery and 30% validation).

C-aAb titers were measured across consecutive blood donation samples in a subset of 72 participants from the validation cohort screening. This included five groups of 12 individuals with high titers of GM-CSF, IFNα, IL-1α, IL-6, and IL-10 c-aAb, respectively. High titer cases were obtained from individuals with MFI above the validation cohort 90th percentile at the point of DBDS inclusion, with at least 3 longitudinal samples available, for a period of at least 3 years. Potential cases with multiple c-aAb titers above this threshold were disqualified. A control group of *N* = 10 was formed from individuals with c-aAb titers below the high-titer threshold for all five investigated c-aAb in the validation cohort. The c-aAb levels were measured in plasma, which had been collected at the time of inclusion into DBDS and stored at −20 °C using a custom-developed xMAP® assay^43,44^. In brief, a mix of seven fluorescent MagPlex® spheres individually conjugated with recombinant human GM-CSF, IFNα, IFNβ, IFNγ, IL-1α, IL-6, or IL-10 were incubated with plasma to capture c-aAb, followed by incubation with PE-conjugated F (ab’)2 anti-human IgG secondary antibody (Thermo Fisher Scientific). With a panel of internally validated c-aAb high-titer and negative controls, sample c-aAb were measured as MFI using a Luminex200® system, with data collected through xPONENT® software.

#### CHB

CHB includes ~500,000 samples from outpatients and hospitalized patients within the Danish Capital Region, with data collection beginning in 2009. Of these, 300,000 have presently been genotyped. The biobank is based on leftover EDTA blood from blood type testing or red cell antibody screenings, which is used to include patients in the cohort^24^. CHB consists of a range of sub-cohorts based on ethical protocol approvals (see above), each with specific inclusion criteria (diseases).

To be included as a case in a CHB sub-cohort, patients must suffer from cohort-specific diseases at any timepoint before death. These diseases are identified through registration in the Danish National Patient Registry (NPR), which has been operational since 1977^45^. Patients with diagnoses unrelated to the sub-cohorts’ criteria are included in the sub-cohorts as controls. Additionally, participants from DBDS were included in the sub-cohorts as cases or controls, depending on the ethical approvals and registered diagnoses in the NPR. In cases where diagnoses were represented in multiple sub-cohorts, we conducted analyses in the cohort with the largest number of cases. This study involved participants in the CHB-DEBD (*N* = 237,444), CHB-CDC (*N* = 148,783), CHB-CID (*N* = 215,512), CHB-PDS (*N* = 203,320) and CHB-OCMS (*N* = 242,599) sub-cohorts.

### Genotyping of DBDS and CHB cohorts

Genotyping of the DBDS and CHB cohorts was conducted on DNA purified from whole-blood inclusion samples following storage at −20 °C. The genotyping process was performed by deCODE Genetics (Iceland) using the Illumina Global Screening Array^24,46^. Prior to imputation, the genotyped dataset underwent the following sample QC: Duplicate samples, samples with genotype rate less than 0.98, and heterozygosity deviations >3 SD from the population were removed, as well as variants with missingness > 10%. In addition, samples with reported sex not matching genotype-determined sex were removed, as well as ancestry outliers (<93% European ancestry), as assigned by a 1000 Genomes-based supervised ADMIXTURE run^47^ and EIGENSOFT (v 6.0.1)^48^. Variant QC included removing variants with a genotype rate lower than 0.95, minor allele frequency (MAF) less than 1% and variants with Hardy–Weinberg equilibrium (HWE) *p* higher than 1 × 10^−6^. A joint variant calling with Graphtyper^49^ formed the basis for the imputation, using an in-house workflow developed at deCODE genetics^50^. The in-house reference panel used for imputation included a total of approximately 25,000 whole-genome sequenced North-Western European individuals, including approximately 8500 Danish individuals sequenced as part of other studies, in addition to variants from the UK 1000 Genomes project phase 3 and HapMap 3^46,51,52^. Only variants with imputation INFO scores above 0.8 were included for the GWAS analyses.

### Genome wide association studies

Before performing GWASs, c-aAb MFI phenotypes were inverse log-rank sum transformed to achieve a normal distribution of the data. Covariates included age at c-aAb sample date (corresponding to DBDS inclusion), age squared, sex, and the first 10 genetic principal components. Sex was defined as a biological, at-birth attribute. Two-sided variant-phenotype associations were tested with an additive mixed model approach, using REGENIE (version 3.3)^53^. Publicly available programs and packages used for this study are listed in Supplementary Table 8.

Due to prior observations of sex differences in c-aAb distributions and clinical associations^16,44,54^, sex-stratified GWASs were performed using the same method and covariates (excluding sex). To identify variants with sex specific effects, we used the --interaction option in REGENIE, which introduced the interaction term between variant and sex to full-cohort GWASs to test whether the genetic effect differed significantly between sexes. Variants with genome-wide significant *p* values for the interaction term were considered to show evidence of sex-specific genetic effects.

An additional GWAS was conducted for a binary “high/low titer” IL-1α c-aAb phenotype, defined as follows: 1 = c-aAb MFI > 90th percentile, and 0 = MFI < 75th percentile. These thresholds were selected a priori based on c-aAb distribution to define the most likely cases of functionally relevant or irrelevant c-aAb and omit “intermediary” c-aAb levels of unclear immunological relevance. A significance threshold of *p* < 5 × 10^−8^ was used to identify genome-wide significant variants.

Related individuals were excluded from the test and validation cohorts prior to GWAS. Relatedness was identified using PLINK 2.00 based on the king-cutoff command (using the King robust approach) with the kinship coefficient cutoff of > *p*-hat value 12.5%, removing 1st, 2nd, and 3rd degree relatives. Any individuals in the validation cohort exceeding this threshold for a discovery cohort individual were also excluded. Further QC selected for HWE *p* < 1 × 10^−10^. All variants were mapped using NCBI Build 38 for rsID^55^.

#### Variant validation

Genome-wide significant variants from the discovery cohort were validated using the corresponding c-aAb GWAS output from the validation cohort. Summary statistics from the validation cohort were filtered to include only variants identified as significant in the discovery cohort by rsID, with validation cohort *p* values adjusted for multiple testing using the Bonferroni–Holm correction. Variants with *p* < 0.05 in the validation cohort were considered validated.

#### Heritability estimation

Observed heritability was estimated for our c-aAb GWAS summary statistics using the LD score regression pipeline (version 1.0.1) developed by Bulik et al.^56^. Liftover to hg19 genomic build and pre-processing using the included “MungeSumstats” pipeline were performed prior to running LDSC using default settings with 1000 Genomes European LD reference^57^, and the MHC region was excluded.

#### Multi-trait GWAS

The presence of shared vs unique genetic architecture across our c-aAb GWAS results was assessed by multi-trait GWAS analysis, performed pairwise for all c-aAb summary statistics combinations using the Joint Analysis of Summary Statistics (JASS, version 2.2) Python pipeline^58^. Following harmonization of allele coding and genomic positions for the paired summary statistics (lifted to hg19) through standard JASS pre-processing, Multi-trait GWASs were computed using the suggested 1703 European LD-independent regions defined by Berisa and Pickrell^59^, and 1000 Genomes European LD reference^57^. Default JASS settings were applied.

Regions were interpreted as plausibly “shared” if the following criteria were met: (1) a joint JASS *p* < 2.9 × 10^−5^ (corresponding to Bonferroni correction of *p* = 0.05 for 1,703 regions); (2) both phenotypes showed univariate association *p* < 1 × 10^−5^; and (3) matching *z*-score directionality for the two phenotypes. The univariate *p* threshold of 1 × 10^−^^5^ was chosen to allow for identification of statistically significant regions in joint tests based on modest, but non-null effects in the univariate tests.

Regions were interpreted as “carried by a specific phenotype” when joint association *p* was <2.9 × 10^−^^5^, and only one univariate test expressed a *p* < 1 × 10^−^^5^. Regions were interpreted as indicative of “opposite-direction pleiotropy” when the joint test *p* was <2.9 × 10^−5^, both univariate *p* values were <1 × 10^−5^, and the *z*-scores for the two phenotypes had opposite directionality.

### Functional mapping and annotation of GWAS

#### Gene mapping

GWAS results were further explored for potential causal genes related to c-aAb using version 1.6.6 of the online FUMA platform, following lift-over to the hg19 genetic build^40^.

Independent variants were identified using an LD threshold of *r*^2^ < 0.6, with lead variants identified from independent variants with *r*^2^ > 0.1. Variants were mapped to genes within a 10 kb window using positional mapping. Additionally, genes were mapped to variants based on eQTL data, utilizing the latest version of the GTEx v8 resource^26^ and other available gene expression datasets. Significant gene pairs were identified with a false discovery rate (FDR) < 0.05. FUMA also assigns CADD scores to variants to estimate their deleteriousness, with CADD scores > 12.37 considered most likely to have harmful effects on gene expression^60^. The chromosome 6 MHC region is excluded from FUMA by default, but was chosen to be included in this study due to the subject matter of autoantibodies.

#### MAGMA gene-based test

FUMA functional annotation also included a MAGMA gene-based test of the GWAS summary statistics^40^. This multiple linear regression-based test aggregates gene-level associations based on individual variant-level findings and was performed within a 10 kb positional window. After Bonferroni correction for multiple testing, genes with *p* < 2.6 × 10^−6^ were considered significant.

#### Candidate gene isolation

For each c-aAb GWAS with significant variants, we curated lists of the most likely functionally relevant genes for subsequent gene-set enrichment analysis. A gene was considered a candidate if it met at least two of the following criteria: (1) it was the nearest gene to a lead variant, (2) it was identified through eQTL mapping, or (3) it was significant in the MAGMA gene-based test. Our gene prioritization approach was derived from the methods used by Burns et al., Jurgens et al., Kiryluk et al., and Watanabe et al.^25,40,61,62^.

#### Tissue and gene-set enrichment analyses

The FUMA-associated GENE2FUNC tool was used to perform tissue and pathway GSE analyses using a hypergeometric test to assess the overrepresentation of c-aAb candidate genes in specific tissues or gene-sets. Analyses were conducted using default settings for GTEx v8 and all available gene-sets^26^. Tissues or gene-sets with *p* < 0.05, after Benjamini–Hochberg (FDR) correction, were considered statistically significant. In subsequent conditional analyses, GSE analyses were repeated on candidate gene lists following the removal of overlapping, enriched genes for the most significant gene set identified by the initial GSE analysis.

We also tested for tissue enrichment using a S-LDSC approach developed for the LDSC pipeline^27,56^. Our c-aAb summary statistics were pre-processed for the LDSC-based heritability estimation and tested for enrichment in the 10 major tissue categories defined by Finucane et al. in their original methodological S-LDSC paper^27^. The pipeline was run at default settings with a 1000 Genomes European LD reference^57^, with the MHC region excluded.

### PRS development

PRSs were calculated using version 2.3.3 of the PRSice-2 software^63^, using variants and summary statistics from the c-aAb discovery cohort GWAS as a base. Associations between PRS and c-aAb were evaluated using linear regression and two-sided Wald tests. Using the validation cohort, the best-fit PRS model was defined by the GWAS *p* value threshold, which maximized Nagelkerke’s *r*^2^. This threshold was then used for PRS calculation in the full DBDS cohort (excluding the c-aAb GWAS test cohort). Permutation-derived empirical *p* values were computed by PRSice-2 based on phenotype permutation, accounting for multiple testing across PRS *p* value thresholds.

Inverse log-rank transformed c-aAb MFI values were used as the phenotype with age at sample date and sex as covariates to determine the best full model fit. Clumping was performed within a 250 kb window, with removal of variants in LD using an *r*^2^ threshold of 0.1. Subsequent PRS calculations utilized binary high/low titer c-aAb (as defined above) as the phenotype and followed the same approach using logistic regression.

In cases where statistically significant c-aAb PRSs were identified, these scores were recalculated in the CHB cohorts without clumping, based on c-aAb GWAS summary statistics for variants with *p* values below the established threshold for the best PRS model. Prior to calculating the PRS, the DBDS and CHB cohorts were filtered for relatedness through exclusion of individuals with a kinship coefficient greater than 12.5%. Individuals with ±5 SD principal component-derived ancestry relative to individuals with two Danish parents were also excluded, based on the approach of Olofsson et al. and Brøns et al.^64,65^.

### Statistical analyses

#### Disease definitions

To investigate the association between c-aAb genetic profiles and clinical phenotypes, we conducted Cox regression analyses using c-aAb PRSs as predictors of diseases observed in the CHB cohort. From the diseases available to study in our group, 14 common diseases were chosen as outcomes, as well as any form of cancer. Outcomes were defined using NPR ICD-10 diagnoses from the NPR (Supplementary Data 31). In cases of multiple diagnoses for a given disease, the earliest available diagnosis was selected. ICD-10-equivalent ICD-8 codes were also identified and incorporated, based on the methodology outlined by Pedersen et al.^66^.

#### PRSs as predictors of disease

Cox proportional hazards models were used to assess whether statistically significant c-aAb PRSs predicted the time to disease, as defined above. Survival time was calculated from the point of inclusion in the NPR until diagnosis, death, or the end of follow-up, with participant age as the underlying time scale. Age at inclusion was defined as the age at the earliest observed NPR registration for the relevant disease, with age at inclusion set to zero for individuals born after this date. Follow-up was defined as the latest NPR registration for the relevant disease. All Cox models that used full-cohort-derived PRSs included sex as a covariate. PRSs were adjusted by their SD prior to analysis.

Further analyses were stratified by quartiles of birth year within the cohort: quartile 1 included individuals with the earliest registered birth dates, and quartile 4 included individuals with the most recent birth dates. Subsequent Cox analyses tested individual PRS quantiles as predictors of disease. Binary variables were assigned as follows: a value of 1 for c-aAb PRS values within quantiles 2, 3, 4, or 5, and a value of 0 for PRS values below quantile 1. *P* values were adjusted for multiple testing using the Bonferroni correction. Before conducting these analyses, the CHB cohort was filtered for relatedness, ancestry, and withdrawn consent, as described above.

### Reporting summary

Further information on research design is available in the Nature Portfolio Reporting Summary linked to this article.

## Supplementary information

Supplementary Information

Description of Additional Supplementary Files

Supplementary Data 1–31

Reporting Summary

Transparent Peer Review File

## Source data

Source Data

## Data Availability

The GWAS summary statistics generated in this study have been deposited in the Zenodo database at https://doi.org/10.5281/zenodo.19483219. Individual-level data are protected and are not available due to data privacy laws, as the data are restricted to approved research projects based in Denmark, given the Danish GDPR legislation. Access can be obtained through collaborations upon contacting the DBDS steering committee (info@dbds.dk), following approval from the Regional Committees on Health Research Ethics and the Danish Data Protection Agency. The expected timeframe for responding to access requests is 2–6 weeks, with datasets remaining available for 1–3 months after mutual agreement is reached. Post-GWAS processed data are provided as Supplementary Data 1–24, and variants informing calculation of PRSs as Supplementary Data 25–30 in the “Supplementary Data 1–31” file. GTEx data (v8) were used for analyses in this study through the online FUMA platform, and is accessible in dbGaP (accession number phs000424.v8.p2). Multi-trait GWAS analyses performed using JASS used the European reference panel derived from the 1000 Genomes Project Phase 3 dataset, available from the archived JASS reference files (https://doi.org/10.5281/zenodo.13940447). Heritability and S-LDSC analyses were performed using the 1000 Genomes Project Phase 3 European baseline-LD v2.2 reference model, together with the corresponding HapMap3 regression weights distributed with LDSC (https://doi.org/10.5281/zenodo.7796478). Source data are provided with this paper.
