# Supplementary material for: Genetic architecture of cytokine autoantibodies and associated risk of common diseases
Source: Nat Commun. 2026 Aug 22;17:10053. doi: 10.1038/s41467-026-76394-7 (PMC13594223; doi:10.1038/s41467-026-76394-7)
Supplement: Supplementary file 1 [file 41467_2026_76394_MOESM1_ESM.pdf]

Genetic architecture of cytokine autoantibodies and associated risk of common diseases

Supplementary information

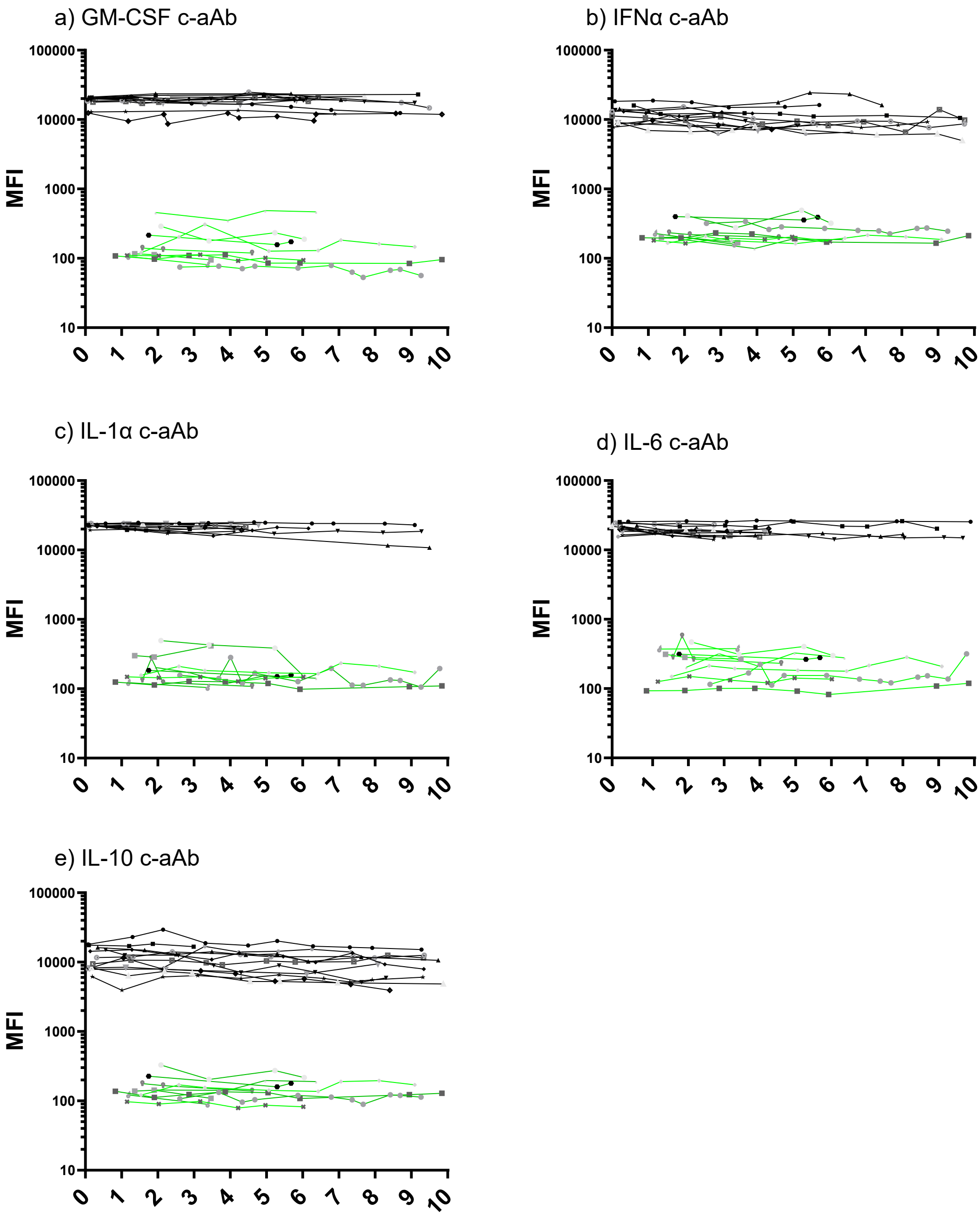

Supplementary Fig. 1: Longitudinal c-aAb monitoring. Median Fluorescence Intensity (MFI) levels of anti-cytokine autoantibodies (c-aAb) targeting a) GM-CSF, b) IFNα, c) IL-1α, d) IL-6, and e) IL-10 in high-titer Danish Blood Donor Study (DBDS) case and control groups over time, denoted by years since first sample. Groups were based on the validation cohort screening of the five c-aAb. C-aAb were monitored in *N* = 5 x 12 cases considered "high-titer" (MFI > 90th validation cohort percentile) for each of the five c-aAb individually (black lines), as well as *N* =10 controls with MFI values below the high-titer threshold for all five c-aAb investigated in this cohort (green lines).

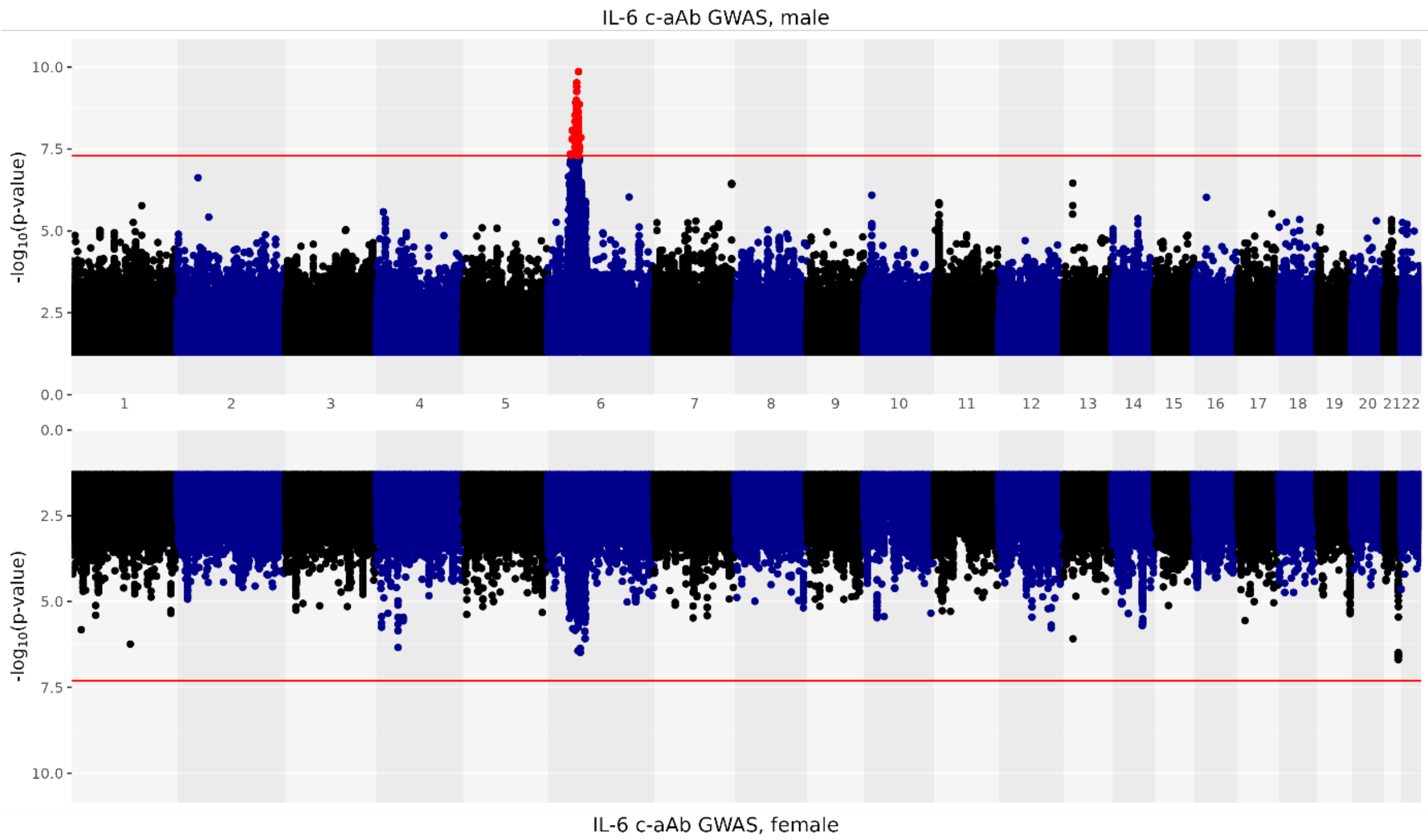

Supplementary Fig. 2: Miami plot for IL-6 anti-cytokine autoantibodies (c-aAb) sex-specific genome-wide association study (GWAS) analyses**.** Analyses were conducted on inverse rank-transformed IL-6 c-aAb using generalized linear mixed models in a cohort of exclusively male (*N* = 3,939) or female (*N* = 4,066) healthy, unrelated Danish Blood Donor Study (DBDS) participants of Northern European ancestry. Age at sample date, age squared and the first 10 Principal components were included as covariates. Genome-wide significant variants ($p < 5 \times 10^{-8}$) are highlighted in red.

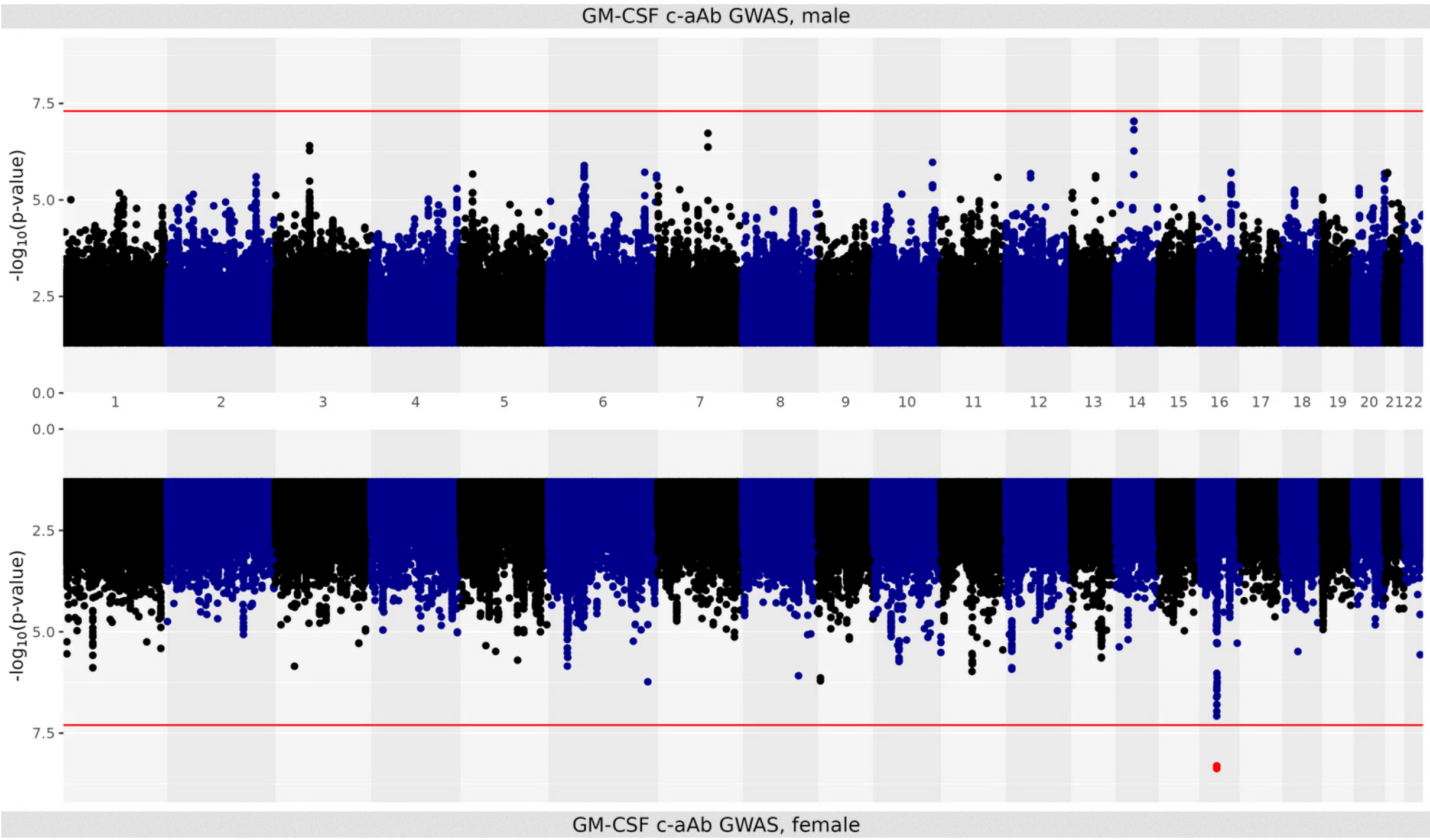

Supplementary Fig. 3: Miami plot for GM-CSF anti-cytokine autoantibodies (c-aAb) sex-specific genome-wide association study (GWAS) analyses. Analyses were conducted on inverse rank-transformed GM-CSF c-aAb using generalized linear mixed models in a cohort of exclusively male (*N* = 3,939) or female (*N* = 4,066) healthy, unrelated Danish Blood Donor Study (DBDS) participants of Northern European ancestry. Age at sample date, age squared and the first 10 principal components were included as covariates. Genome-wide significant variants ($p < 5 \times 10^{-8}$) are highlighted in red.

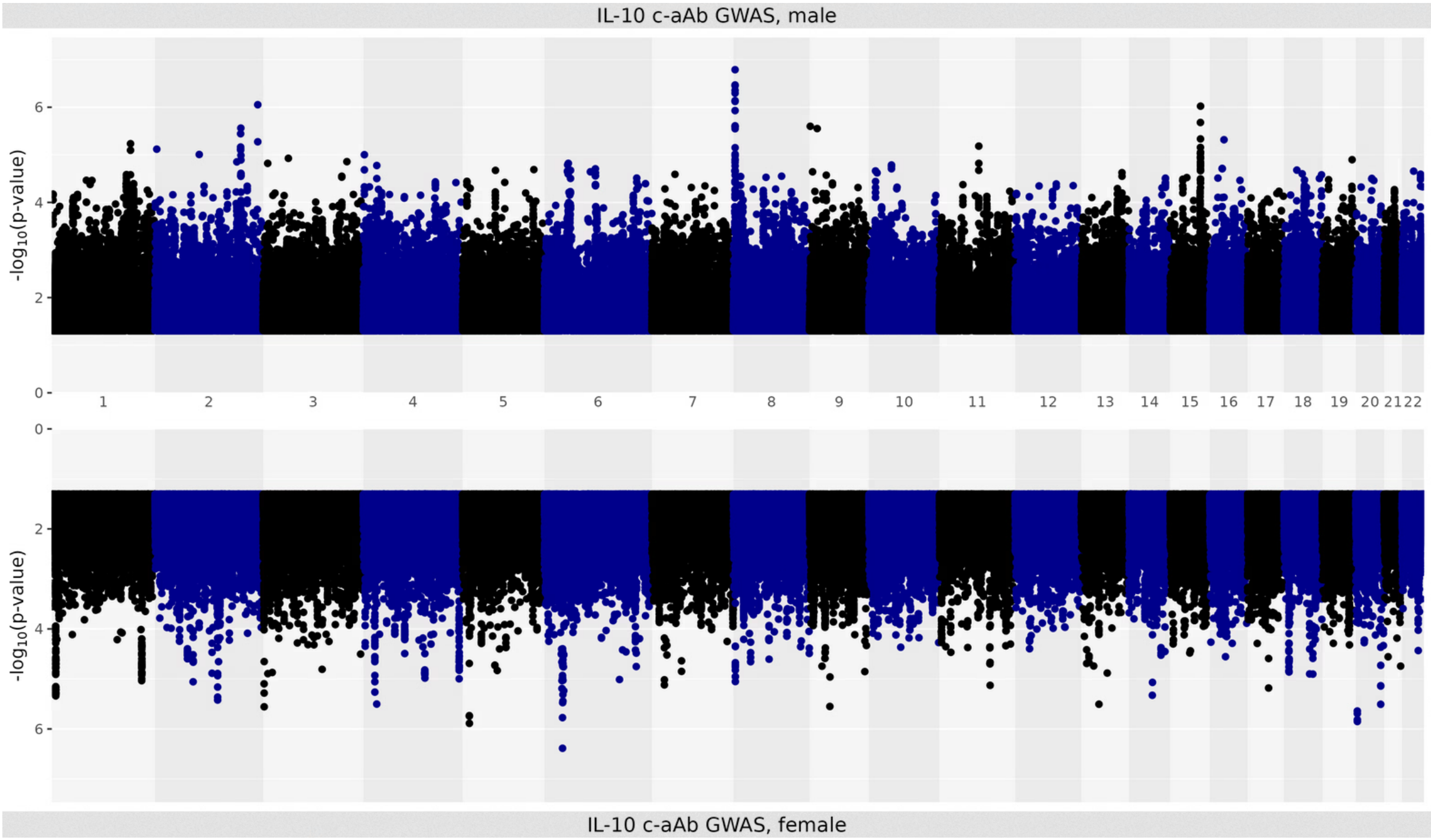

Supplementary Fig. 4: Miami plot for IL-10 anti-cytokine autoantibodies (c-aAb) sex-specific genome-wide association study (GWAS) analyses. Analyses were conducted on inverse rank-transformed IL-10 c-aAb using generalized linear mixed models in a cohort of exclusively male (*N* = 3,939) or female (*N* = 4,066) healthy, unrelated Danish Blood Donor Study (DBDS) participants of Northern European ancestry. Age at sample date, age squared and the first 10 principal components were included as covariates.

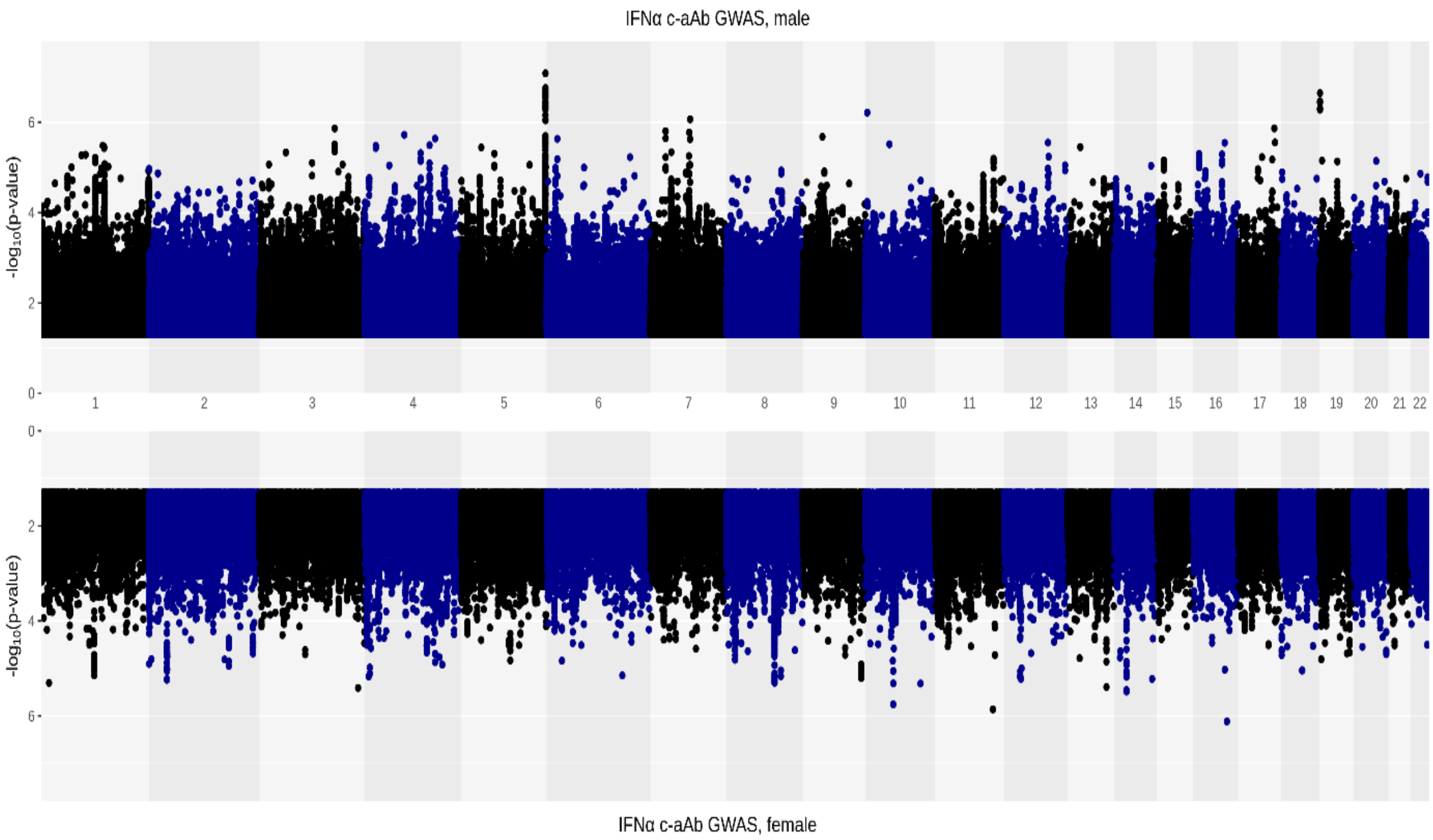

Supplementary Fig. 5: Miami plot for IFNα anti-cytokine autoantibodies (c-aAb) sex-specific genome-wide association study (GWAS) analyses. Analyses were conducted on inverse rank-transformed IFNα c-aAb using generalized linear mixed models in a cohort of exclusively male (*N* = 3,939) or female (*N* = 4,066) healthy, unrelated Danish Blood Donor Study (DBDS) participants of Northern European ancestry. Age at sample date, age squared and the first 10 principal components were included as covariates.

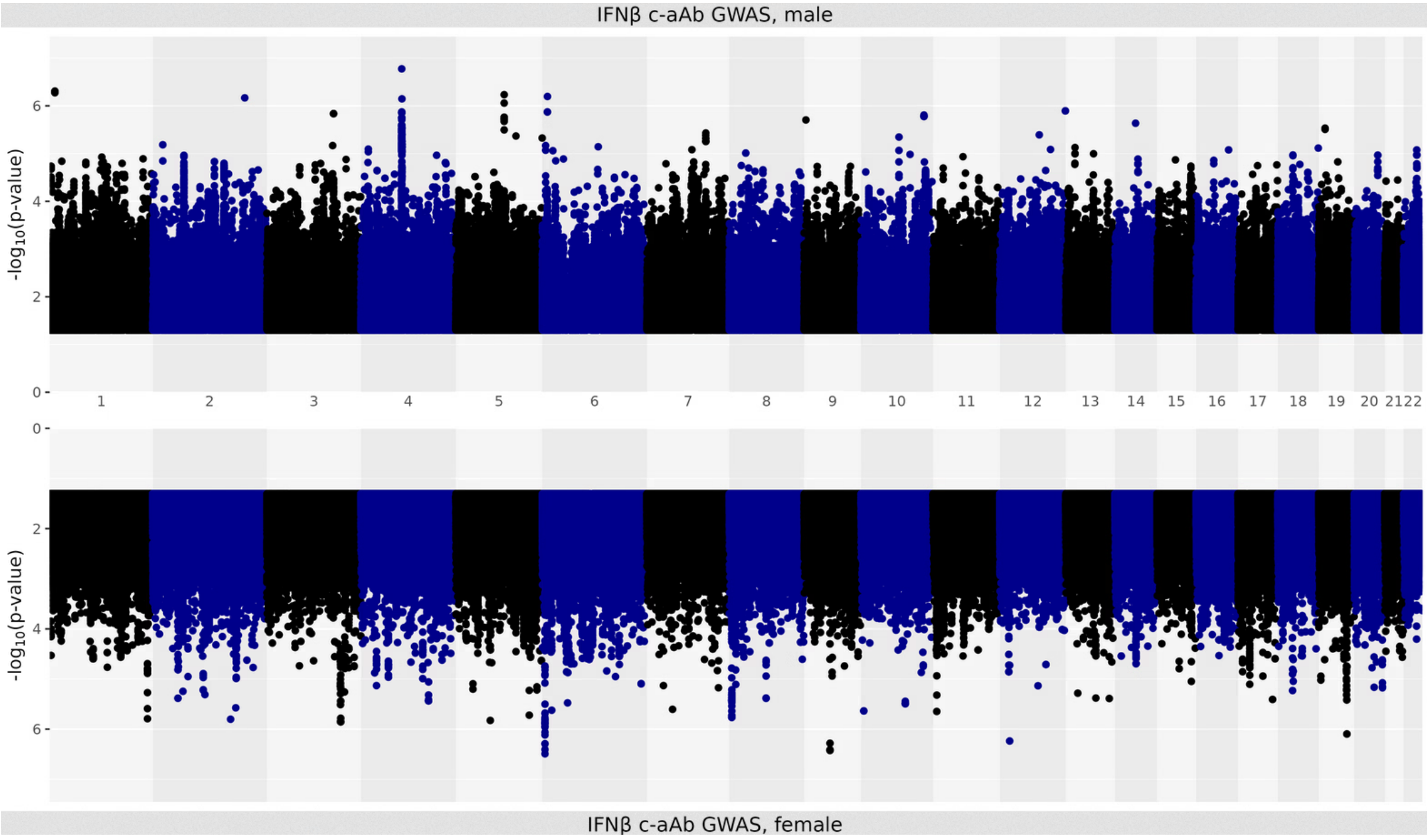

Supplementary Fig. 6: Miami plot for IFNβ anti-cytokine autoantibodies (c-aAb) sex-specific genome-wide association study (GWAS) analyses. Analyses were conducted on inverse rank-transformed IFNβ c-aAb using generalized linear mixed models in a cohort of exclusively male (N = 2,612) or female (N = 2,798) healthy, unrelated Danish Blood Donor Study (DBDS) participants of Northern European ancestry. Age at sample date, age squared and the first 10 principal components were included as covariates.

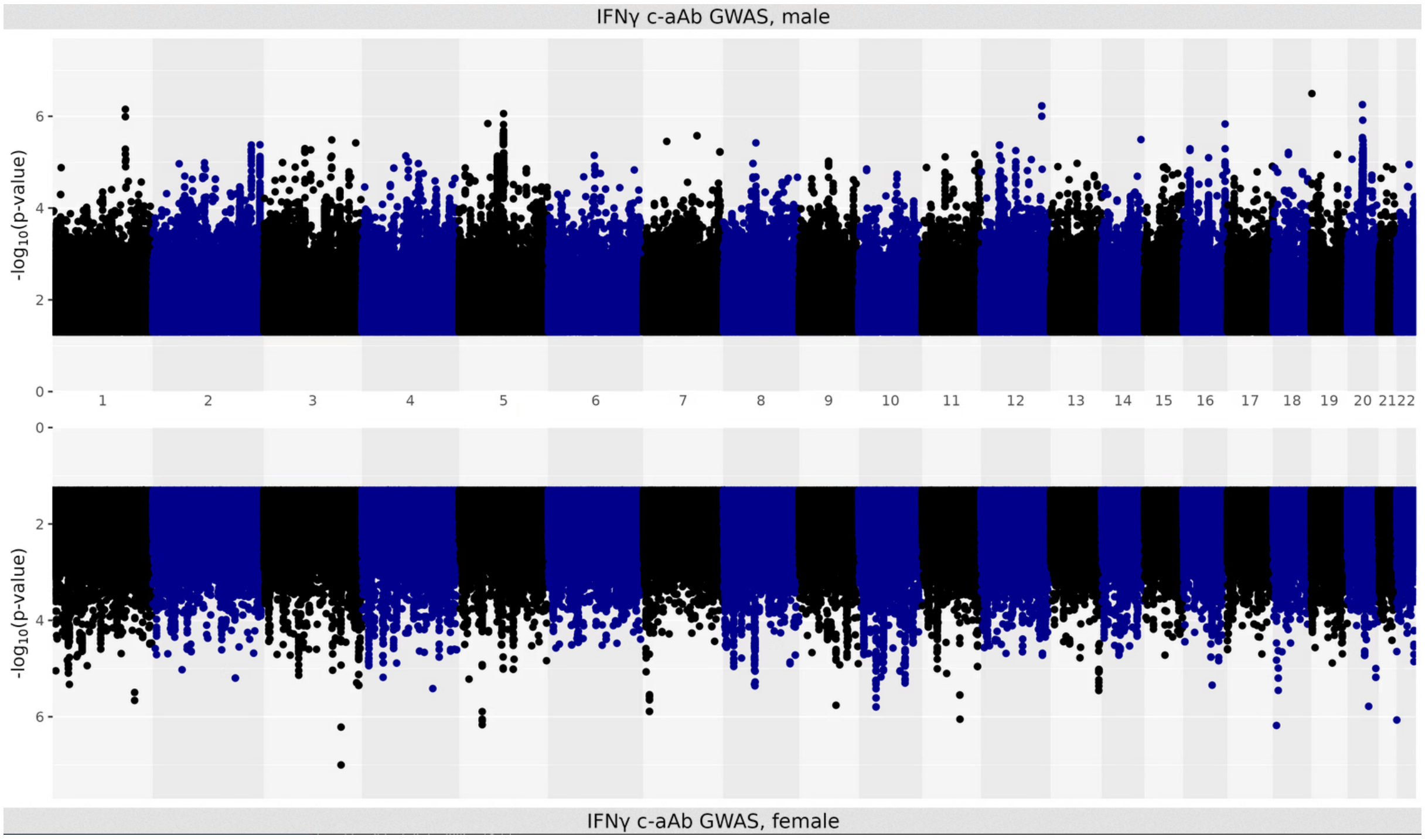

Supplementary Fig. 7: Miami plot for IFNγ anti-cytokine autoantibodies (c-aAb) sex-specific genome-wide association study (GWAS) analyses. Analyses were conducted on inverse rank-transformed IFNγ c-aAb using generalized linear mixed models in a cohort of exclusively male (*N* = 2,612) or female (*N* = 2,798) healthy, unrelated Danish Blood Donor Study (DBDS) participants of Northern European ancestry. Age at sample date, age squared and the first 10 principal components were included as covariates.

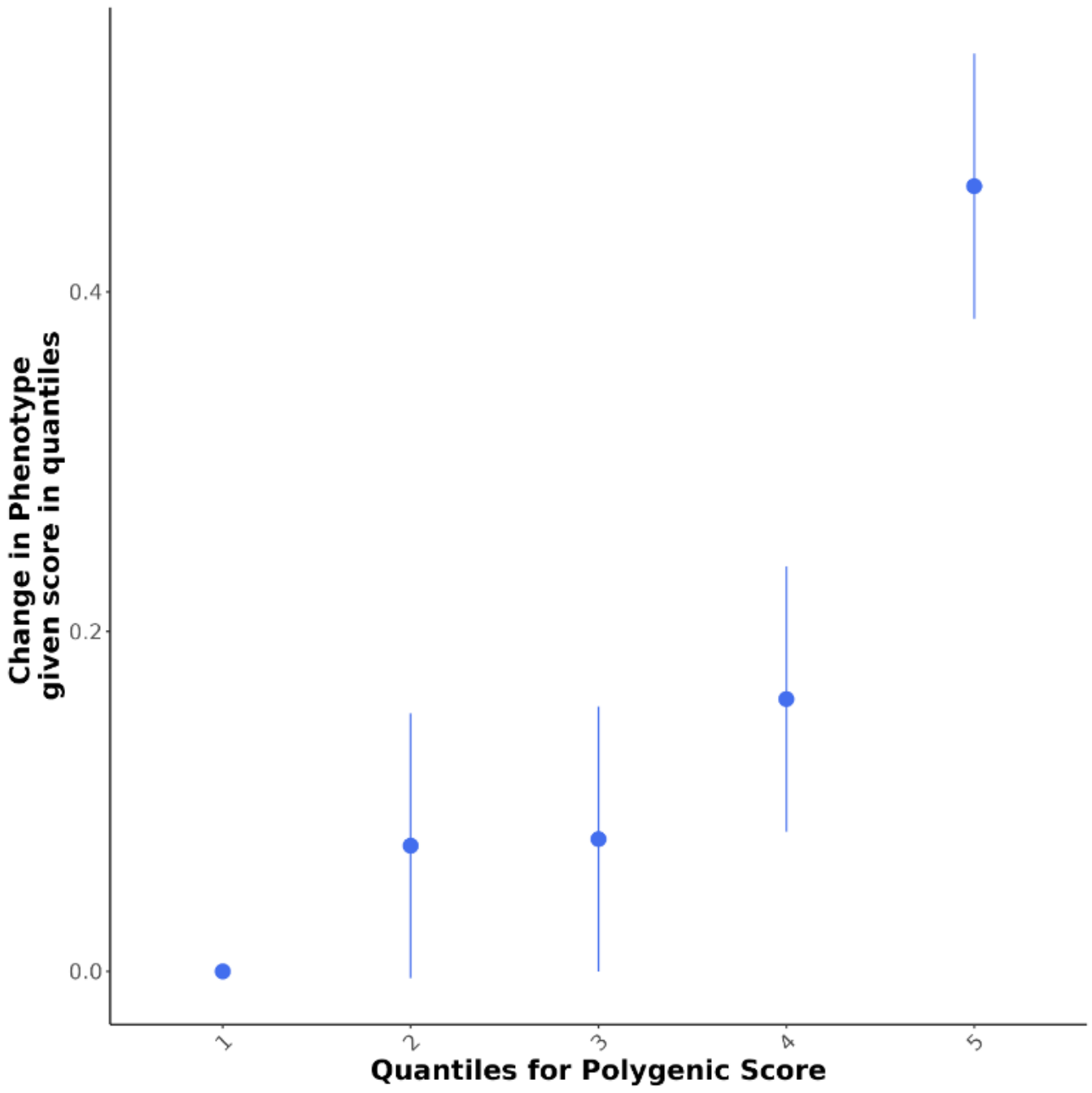

Supplementary Fig. 8: IL-1α anti-cytokine autoantibodies (c-aAb) Polygenic Risk Score (PRS) vs. IL-1α c-aAb phenotype, by quantile. Change in IL-1α c-aAb Median Fluorescence Intensity (MFI) values (inverse log-transformed) with 95% Confidence Interval (CI) are plotted according to quantile of IL-1α c-aAb based PRS. Data are presented relative to the baseline, defined as PRS below the first quantile. Results are based on the full-cohort c-aAb genome-wide association study (GWAS). Source data are provided as a Source Data file.

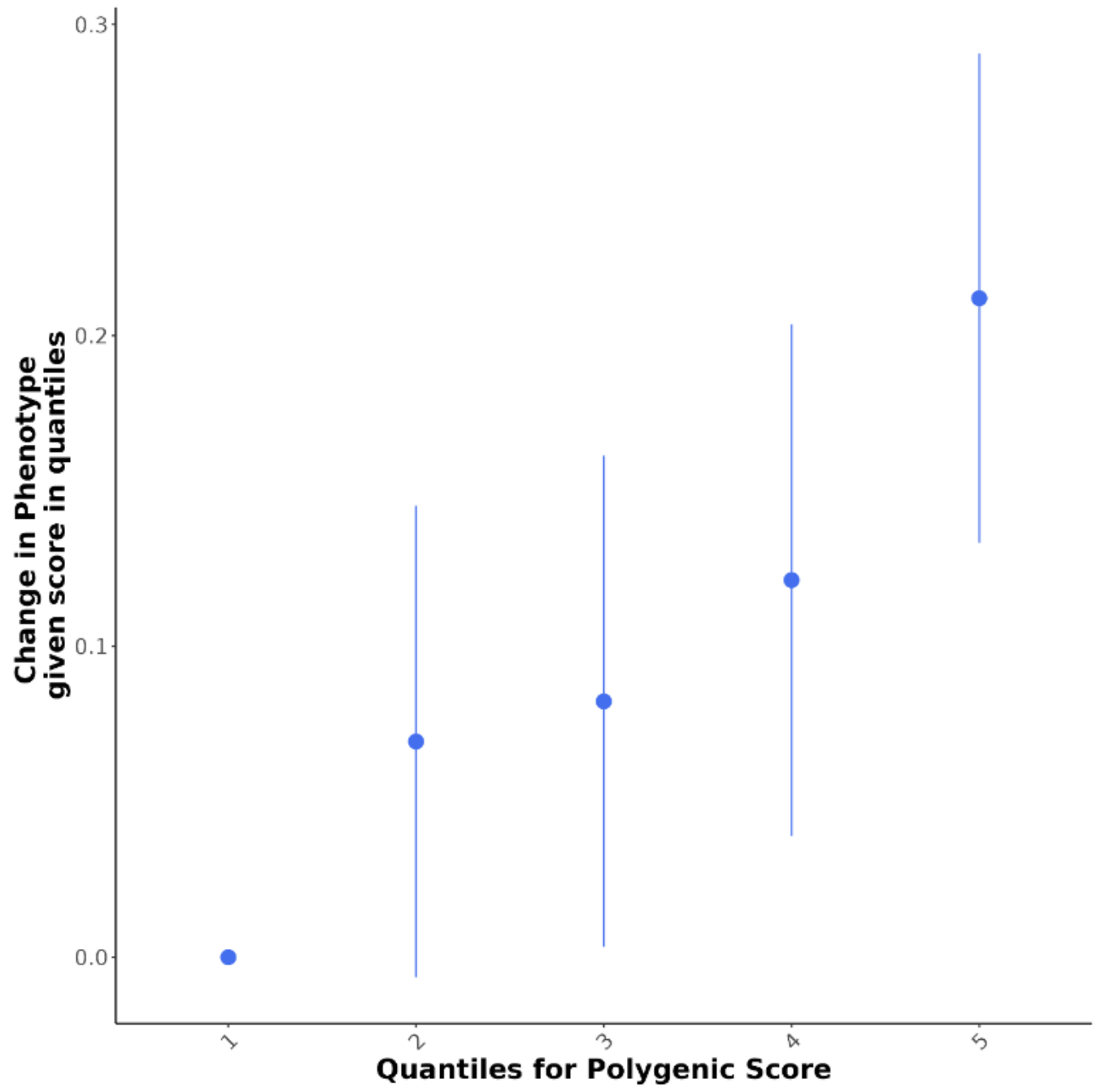

Supplementary Fig. 9: IL-6 anti-cytokine autoantibodies (c-aAb) Polygenic Risk Score (PRS) vs. IL-6 c-aAb phenotype, by quantile. Change in IL-6 c-aAb Median Fluorescence Intensity (MFI) values (inverse log-transformed) with 95% Confidence Interval (CI) are plotted according to quantile of IL-6 c-aAb based PRS. Data are presented relative to the baseline, defined as PRS below the first quantile. Results are based on the full-cohort c-aAb genome-wide association study (GWAS). Source data are provided as a Source Data file.

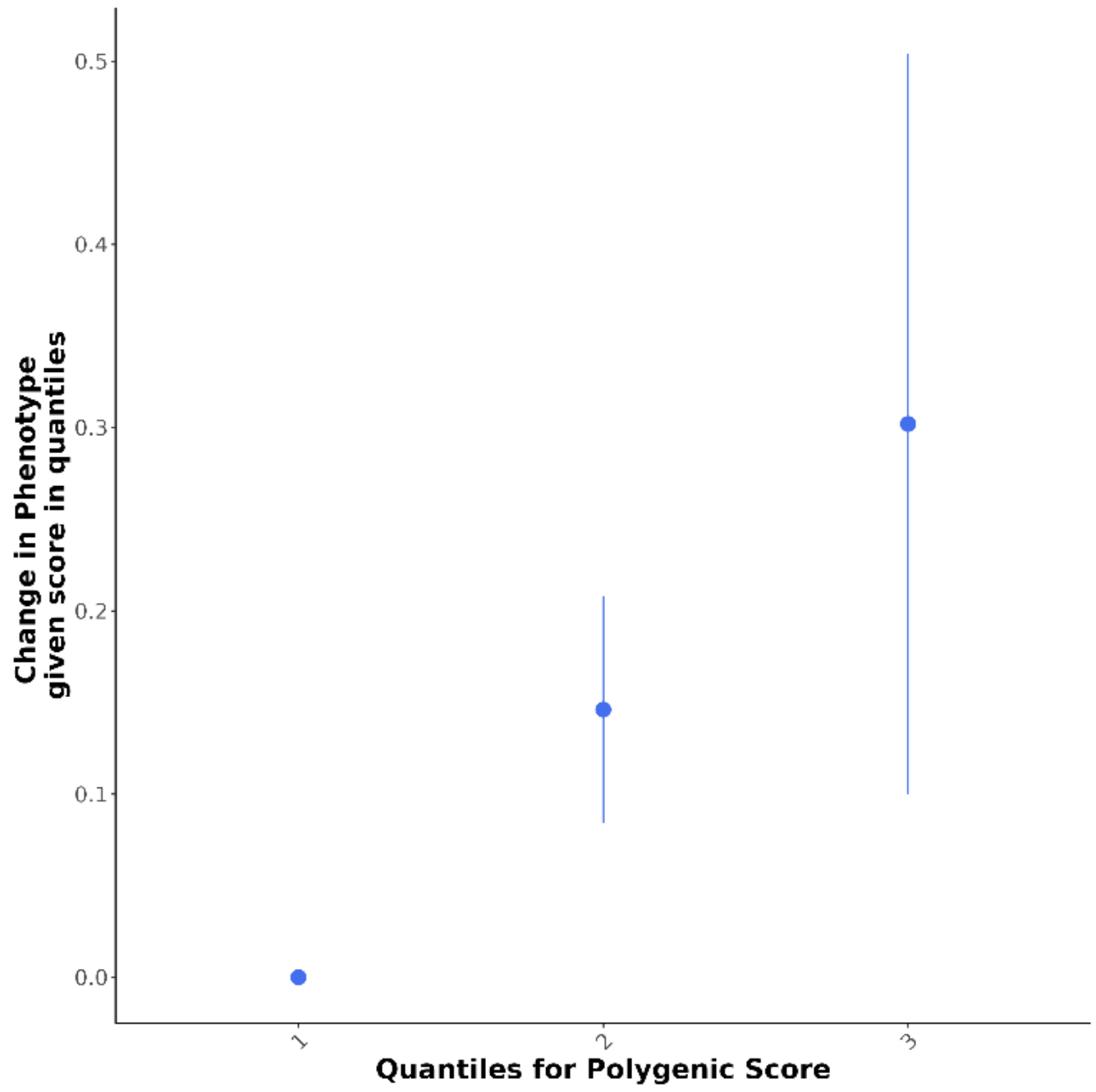

Supplementary Fig. 10: IL-10 anti-cytokine autoantibodies (c-aAb) Polygenic Risk Score (PRS) vs. IL-10 c-aAb phenotype, by quantile. Change in IL-10 c-aAb Median Fluorescence Intensity (MFI) values (inverse log-transformed) with 95% Confidence Interval (CI) are plotted according to quantile of IL-10 c-aAb based PRS. Data are presented relative to the baseline, defined as PRS below the first quantile. Results are based on the full-cohort c-aAb genome-wide association study (GWAS). Source data are provided as a Source Data file.

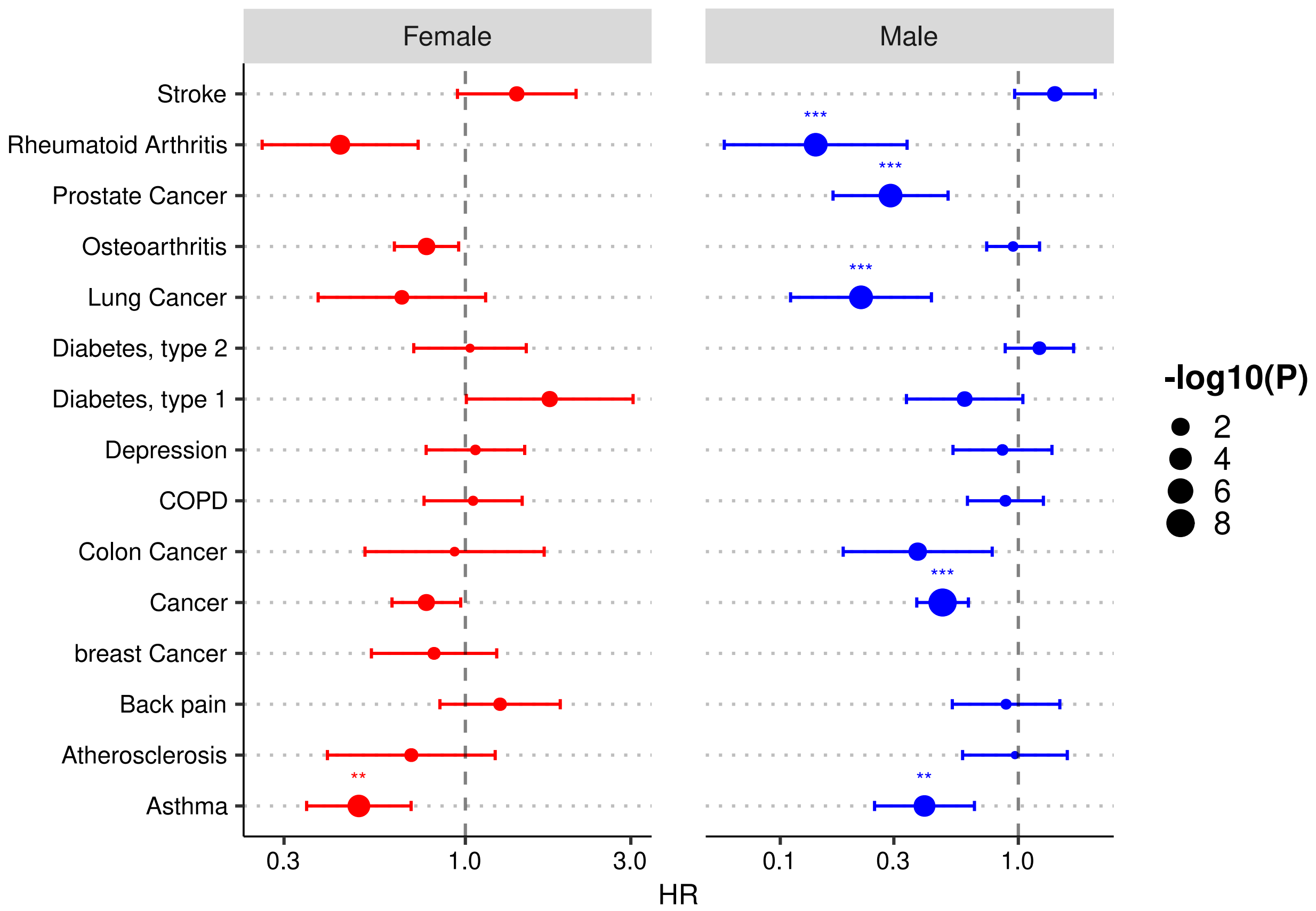

Supplementary Fig. 11: Sex-specific IL-1α anti-cytokine autoantibodies (c-aAb) Polygenic Risk Scores (PRS) vs. risk of common diseases. Data are presented as Standard Deviation (SD)-adjusted Hazard Ratios (HR) for time to disease diagnosis with 95% Confidence Intervals (CI), derived from two-sided Cox proportional hazards regression analyses using relevant sub-cohorts from the Copenhagen Hospital Biobank (see supplementary tables 3-7). PRSs for IL-1α c-aAb, developed from sex-specific genome-wide association study (GWAS), were used as predictors. Males shown in blue. Females shown in red. Survival time was defined as the period between patient registry entry and diagnosis, with age as the underlying time scale. *P* values were adjusted for multiple comparisons. * = $p < 0.05$, ** = $p < 0.01$, and *** = $p < 0.001$. Source data are provided as a Source Data file.

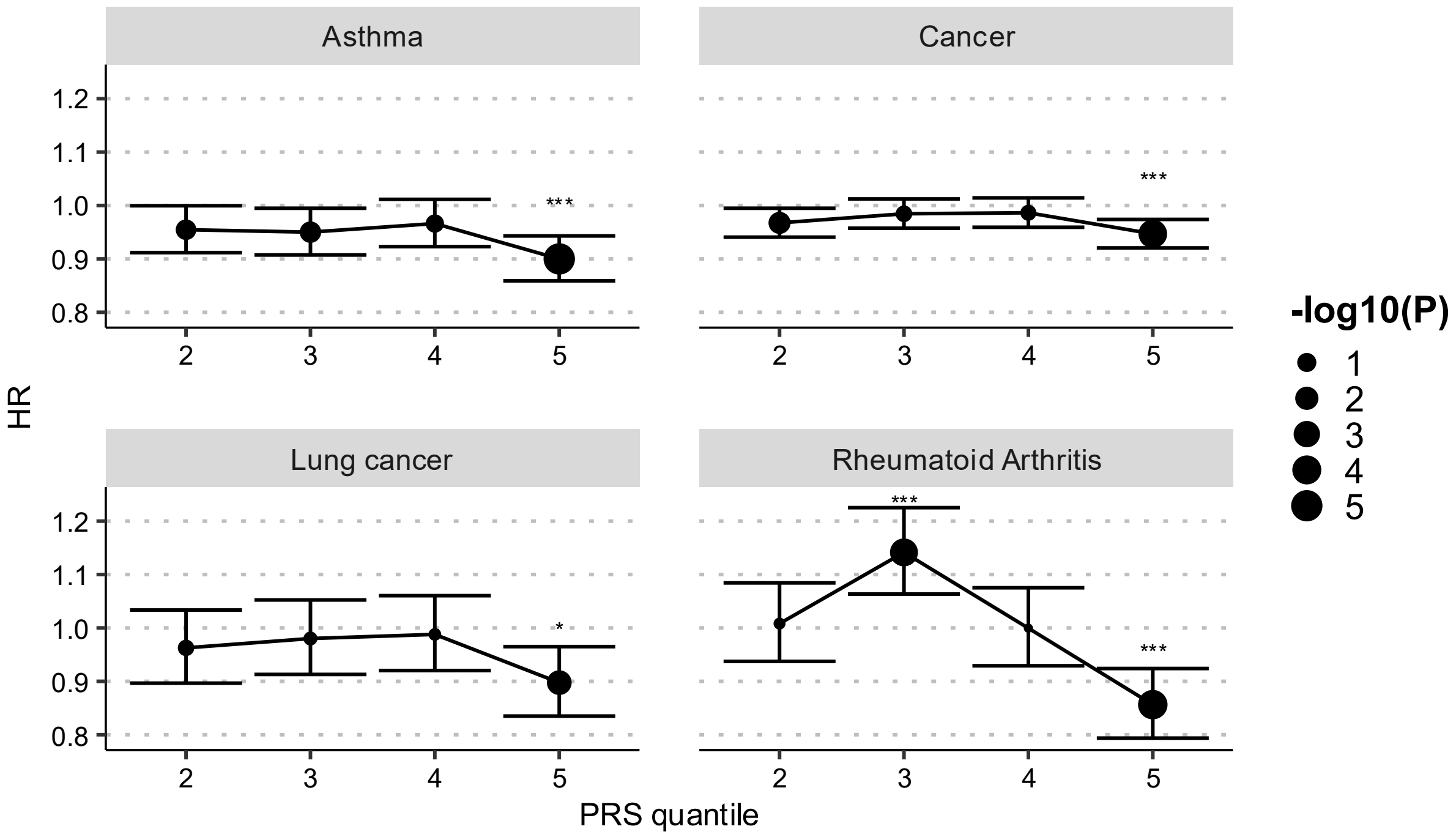

Supplementary Fig. 12: IL-1α anti-cytokine autoantibodies (c-aAb) Polygenic Risk Score (PRS) quantile vs. risk of common diseases. Data are presented as Hazard Ratios (HR) for time to disease diagnosis with 95% Confidence Intervals (CI), derived from two-sided Cox proportional hazards regression analyses using IL-1α c-aAb PRS quantiles as predictors. PRS quantile predictors were defined as binary variables with 1 = PRS within either the 2nd, 3rd, 4th or 5th quantile, and 0 = PRS within a 1st quantile baseline. Results are based on the full-cohort c-aAb PRS. Tests were performed in relevant sub-cohorts from the Copenhagen Hospital Biobank based on diagnosis, including The CHB study on Cancer using the Danish Cancer biobank (Cancer and Lung Cancer, N = 59,509 per binary quantile predictor) and the CHB-study on Chronic Inflammatory Diseases (Asthma and Rheumatoid Arthritis, N = 86,187 per binary quantile predictor). Survival time was calculated as the interval between patient registry entry and diagnosis, with age as the underlying time scale. Sex was included as a covariate. P values were adjusted for multiple comparisons. * = $p < 0.05$, ** = $p < 0.01$, and *** = $p < 0.001$. Source data are provided as a Source Data file.

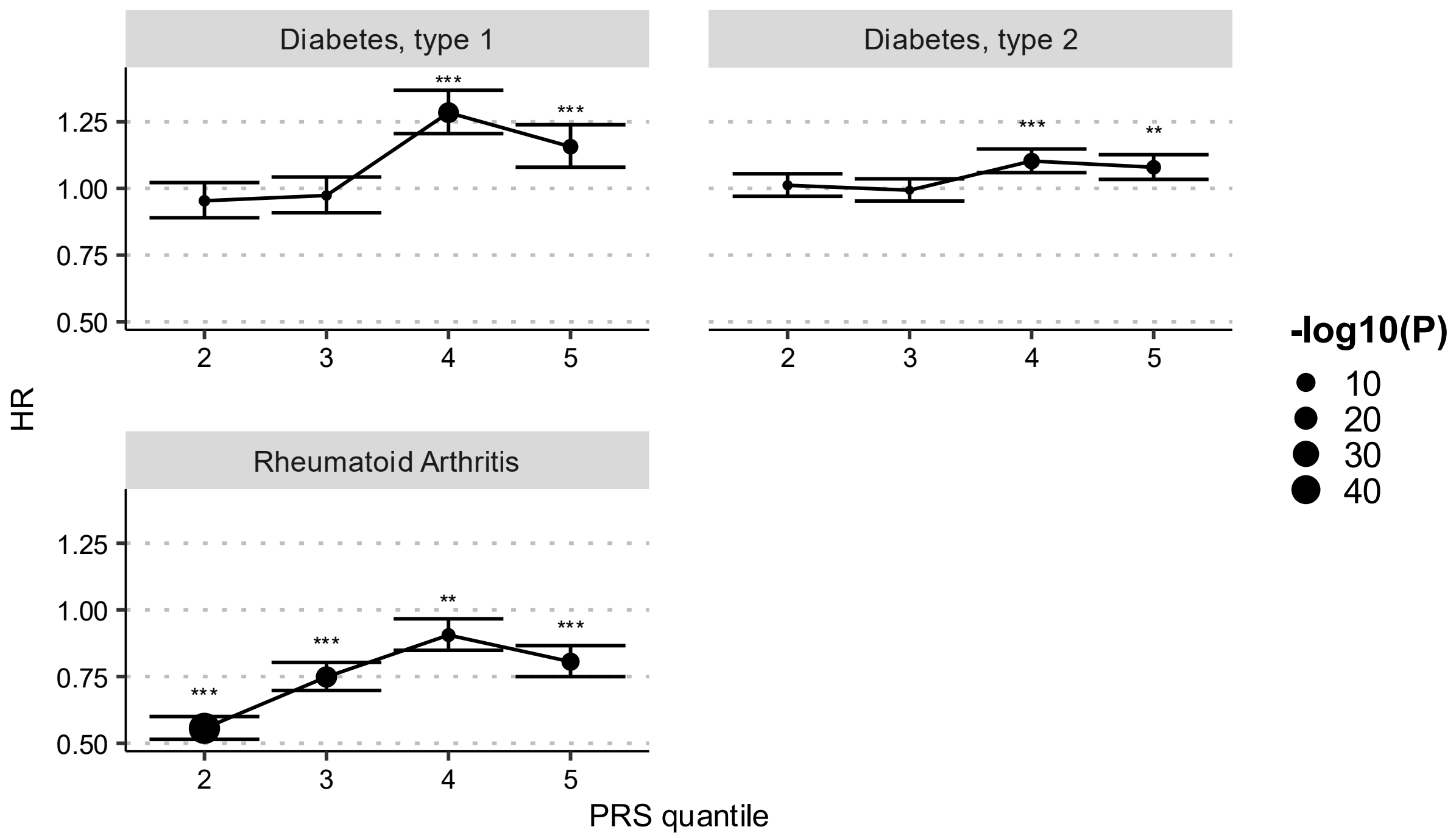

Supplementary Fig. 13: IL-6 anti-cytokine autoantibodies (c-aAb) Polygenic Risk Score (PRS) quantile vs. risk of common diseases. Data are presented as Hazard Ratios (HR) for time to disease diagnosis with 95% Confidence Intervals (CI), derived from two-sided Cox proportional hazards regression analyses using IL-6 c-aAb PRS quantiles as predictors. PRS quantile predictors were defined as binary variables with 1 = PRS within either the 2nd, 3rd, 4th or 5th quantile, and 0 = PRS within a 1st quantile baseline. Results are based on the full-cohort c-aAb PRS. Tests were performed in relevant sub-cohorts from the Copenhagen Hospital Biobank based on diagnosis, including the CHB-study on Chronic Inflammatory Diseases (Rheumatoid Arthritis, *N* = 86,187 per binary quantile predictor), and the CHB-study on Oral-Cardiometabolic Health (Diabetes, *N* = 97,039 per binary quantile predictor). Survival time was calculated as the interval between patient registry entry and diagnosis, with age as the underlying time scale. Sex was included as a covariate. *P* values were adjusted for multiple comparisons. * = $p < 0.05$, ** = $p < 0.01$, and *** = $p < 0.001$. Source data are provided as a Source Data file.

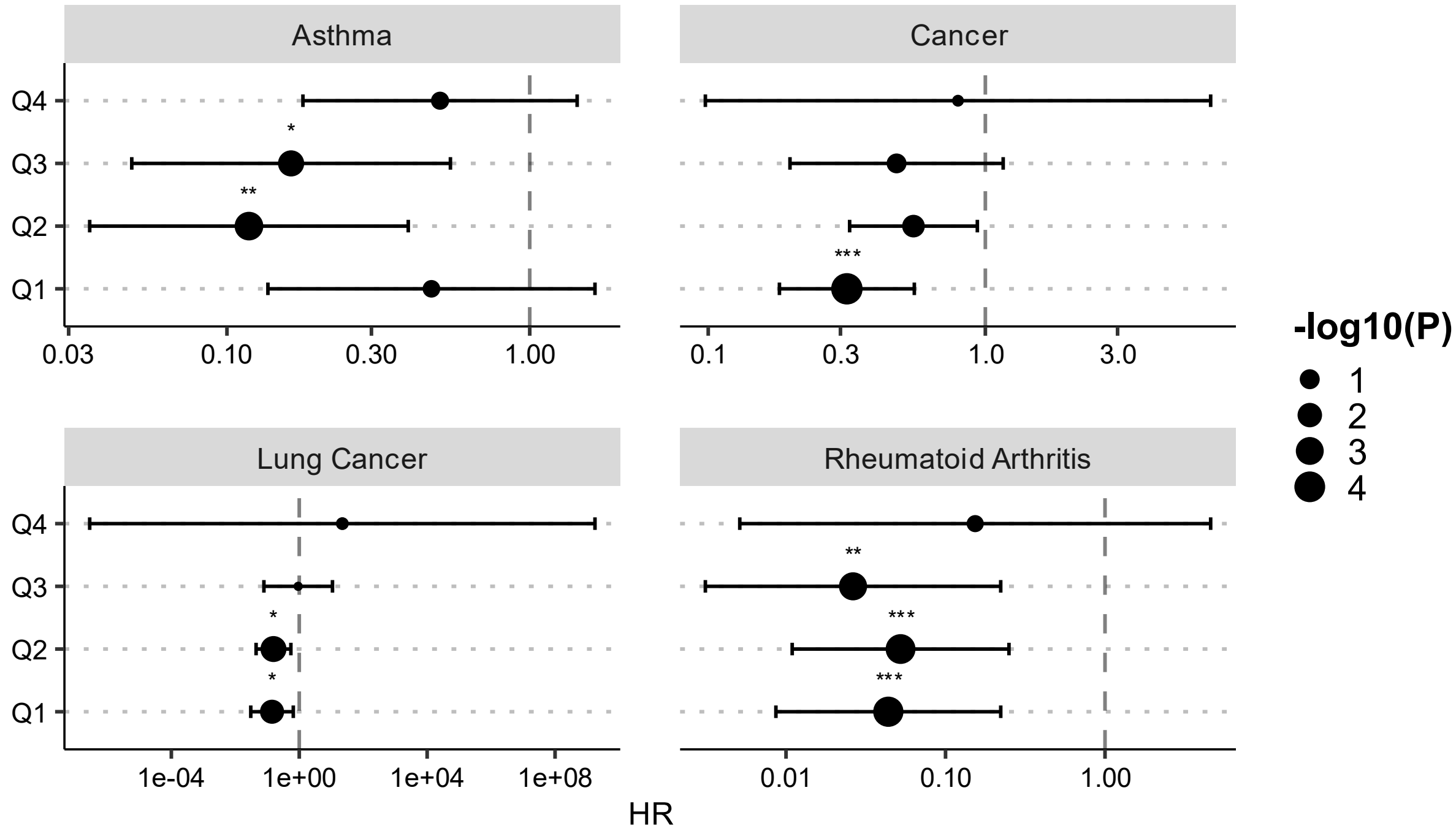

Supplementary Fig. 14: IL-1α anti-cytokine autoantibodies (c-aAb) Polygenic Risk Score (PRS) vs. risk of common diseases, by birthyear. Data are presented as Hazard Ratios (HR) for time to disease diagnosis with 95% Confidence Intervals (CI), derived from two-sided Cox proportional hazards regression analyses, stratified by birth year quartiles, using Standard deviation (SD)-adjusted IL-1α c-aAb PRS as a predictor. Results are based on the full-cohort c-aAb PRS. Tests were performed in relevant sub-cohorts from the Copenhagen Hospital Biobank based on diagnosis including The CHB study on Cancer using the Danish Cancer biobank (Cancer and Lung Cancer, Q1 = 1907-1940, Q2 = 1940-1955, Q3 = 1955-1974, Q4 = 1974-2000, *N* = 37,193 for each age quartile) and the CHB-study on Chronic Inflammatory Diseases (Asthma and Rheumatoid Arthritis, Q1 = 1907-1943, Q2 = 1943-1957, Q3 = 1957-1975, Q4 = 1975-2001, *N* = 53,880 for each age quartile). Survival time was calculated from the period between registry entry and diagnosis, with age as the underlying time scale. Sex was included as a covariate. *P* values were adjusted for multiple comparisons. * = $p < 0.05$, ** = $p < 0.01$, and *** = $p < 0.001$. Source data are provided as a Source Data file.

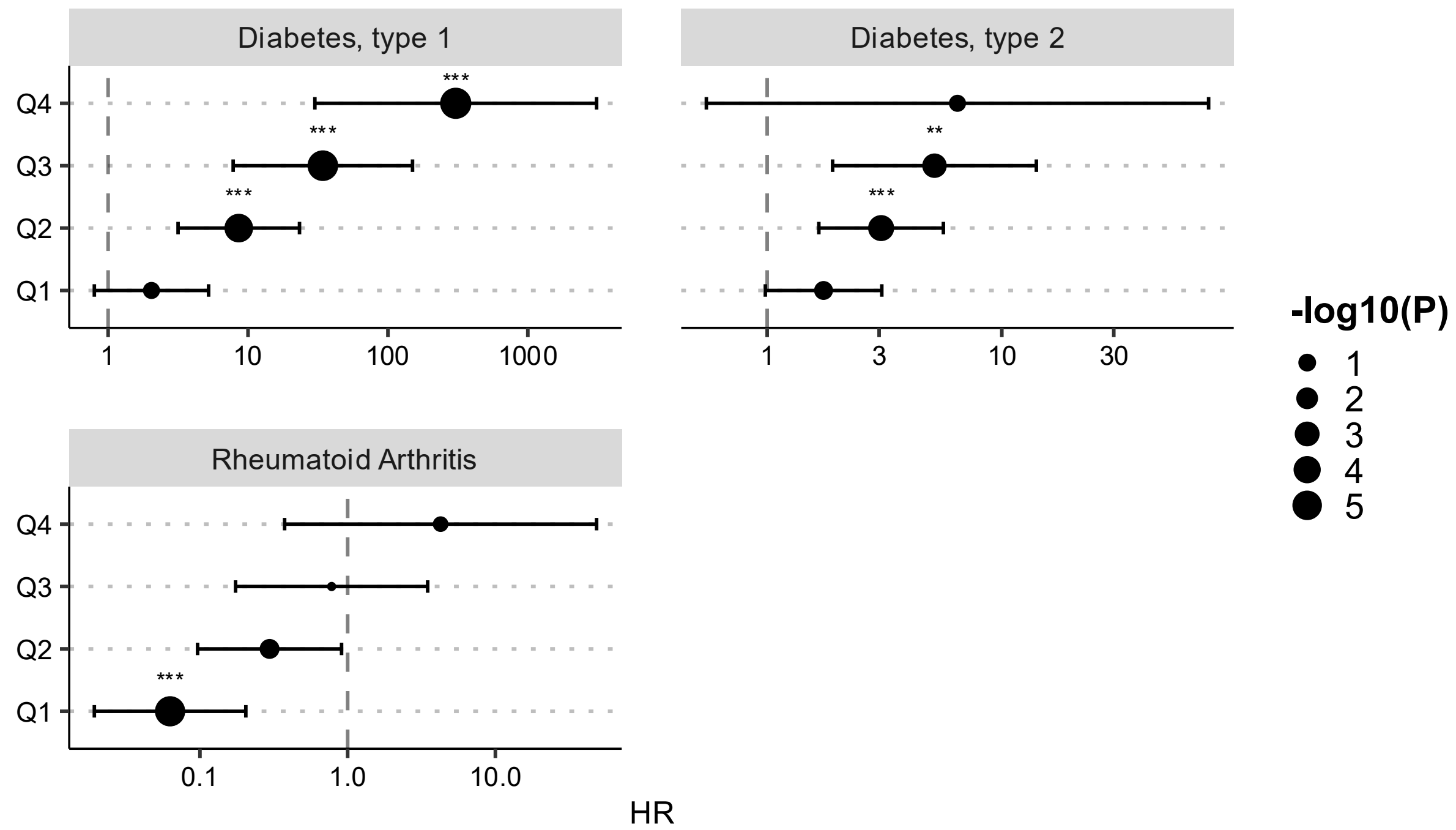

Supplementary Fig. 15: IL-6 anti-cytokine autoantibodies (c-aAb) Polygenic Risk Score (PRS) vs. risk of common diseases, by birthyear. Data are presented as Hazard Ratios (HR) for time to disease diagnosis with 95% Confidence Intervals (CI), derived from two-sided Cox proportional hazards regression analyses, stratified by birth year quartiles, using Standard deviation (SD)-adjusted IL-6 c-aAb PRS as a predictor. Results are based on the full-cohort c-aAb PRS. Tests were performed in relevant sub-cohorts from the Copenhagen Hospital Biobank based on diagnosis including the CHB-study on Chronic Inflammatory Diseases (Rheumatoid Arthritis, Q1 = 1907-1943, Q2 = 1943-1957, Q3 = 1957-1975, Q4 = 1975-2001, *N* = 53,880 for each age quartile) and the CHB-study on Oral-Cardiometabolic Health (Diabetes, Q1 = 1907-1944, Q2 = 1944-1958, Q3 = 1958-1976, Q4 = 1976-2001, *N* = 60,654 for each age quartile). Survival time was calculated from the period between registry entry and diagnosis, with age as the underlying time scale. Sex was included as a covariate. *P* values were adjusted for multiple comparisons. * = $p < 0.05$, ** = $p < 0.01$, and *** = $p < 0.001$. Source data are provided as a Source Data file.

Supplementary table 1: Main DBDS discovery and validation cohort description		
	DBDS discovery cohort	DBDS validation cohort
Mean age at DBDS start, years (SD)	43.3 (13.7)	39.2 (12.2)
Male sex, N (%)	3,774 (48.7)	3,679 (51.8)
Median IL-1α c-aAb, MFI (IQR)	404 (225:774)	520 (370:811)
High-titer IL-1α c-aAb, MFI (N)	2,215 (764)	1,962 (681)
Low-titer IL-1α c-aAb, MFI (N)	774 (5,802)	811 (5,363)
Median IL-6 c-aAb, MFI (IQR)	613 (377: 1,005)	661 (539: 943)
High-titer IL-6 c-aAb, MFI (N)	2,152 (751)	2,006 (697)
Low-titer IL-6 c-aAb, MFI (N)	1,005 (5,913)	943 (5,417)
Median IL-10 c-aAb, MFI (IQR)	221 (137:420)	283 (240:356)
High-titer IL-10 c-aAb, MFI (N)	690.8 (765)	491 (699)
Low-titer IL-10 c-aAb, MFI (N)	420 (5,810)	356 (5,335)
Median GM-CSF c-aAb, MFI (IQR)	197 (119:375)	232 (190:306)
High-titer GM-CSF c-aAb, MFI (N)	637 (758)	495 (703)
Low-titer GM-CSF c-aAb, MFI (N)	375 (5,791)	306 (5,327)
Median IFNα c-aAb, MFI (IQR)	280 (171:479)	336 (299:407)
High-titer IFNα c-aAb, MFI (N)	777 (768)	590 (699)
Low-titer IFNα c-aAb, MFI (N)	479 (5,994)	407 (5,399)

Demographic Overview of the DBDS-Based discovery and Validation Cohorts (*N* = 7,769 for discovery cohort, *N* = 7,106 for validation cohort) for IL-1α, IL-6, IL-10, IFNα, and GM-CSF c-aAb Phenotypes.
C-aAb distribution: Median MFI with interquartile range (IQR).
High-titer" c-aAb: MFI > 90th percentile threshold in the c-aAb screened cohort; considered to be individuals most likely to experience c-aAb mediated immunomodulation.

Supplementary table 2: DBDS IFNβ and IFNγ c-aAb discovery and validation cohort description		
	DBDS IFNβ, IFNγ discovery cohort	DBDS IFNβ, IFNγ validation cohort
Mean age at DBDS start, years (SD)	43.4 (13.7)	43.9 (13.6)
Male sex, N (%)	2,583 (48.4)	1,253 (48.8)
Median IL-1α c-aAb, MFI (IQR)	370 (216:741)	362 (216:741)
High-titer IL-1α c-aAb, MFI (N)	2,485.4 (509)	2,035 (265)
Low-titer IL-1α c-aAb, MFI (N)	741 (3,933)	741 (1,726)
Median IL-6 c-aAb, MFI (IQR)	582 (359:1,001)	579 (368:983)
High-titer IL-6 c-aAb, MFI (N)	2,214.6 (525)	2,260 (246)
Low-titer IL-6 c-aAb, MFI (N)	1,001 (3,992)	983 (1,793)
Median IL-10 c-aAb, MFI (IQR)	204 (132;375)	208 (133:379)
High-titer IL-10 c-aAb, MFI (N)	652.8 (527)	666 (243)
Low-titer IL-10 c-aAb, MFI (N)	375 (3.963)	379 (1,713)
Median GM-CSF c-aAb, MFI (IQR)	180 (115:334)	183 (114:340)
High-titer GM-CSF c-aAb, MFI (N)	619.8 (560)	646 (222)
Low-titer GM-CSF c-aAb, MFI (N)	334 (3.941)	340 (1,718)
Median IFNα c-aAb, MFI (IQR)	259 (165:457)	259 (162:445)
High-titer IFNα c-aAb, MFI (N)	784.8 (529)	790.2 (241)
Low-titer IFNα c-aAb, MFI (N)	457 (3.987)	445 (1,714)
Median IFNβ c-aAb, MFI (IQR)	154 (98:313)	154 (97:321)
High-titer IFNβ c-aAb, MFI (N)	623 (501)	637.3 (207)
Low-titer IFNβ c-aAb, MFI (N)	313 (3,906)	321 (1,737)
Median IFNγ c-aAb, MFI (IQR)	719 (447:1,267)	687 (442:1,206)
High-titer IFNγ c-aAb, MFI (N)	2,528 (514)	2,353 (223)
Low-titer IFNγ c-aAb, MFI (N)	1,267 (3,986)	1,206 (1,718)

Demographic Overview of the DBDS-Based discovery and Validation Cohorts (*N* = 5,410 for discovery cohort, *N* = 2,346 for validation cohort) for IFNβ and IFNγ c-aAb Phenotypes.
C-aAb distribution: Median MFI with interquartile range (IQR).
"High-titer" c-aAb: MFI > 90th percentile threshold; considered to be individuals most likely to experience c-aAb mediated immunomodulation.

Supplementary table 3: CHB-CDC cohort description							
	Full Cohort	IL-1α c-aAb Low PRS	IL-1α c-aAb Intermediate PRS	IL-1α c-aAb High PRS	IL-6 c-aAb Low PRS	IL-6 c-aAb Intermediate PRS	IL-6 c-aAb High PRS
Mean age at registry start, years (SD)	21.9 (17.9)	21.4 (17.8)	21.9 (17.9)	21.7 (18.1)	21.7 (17.8)	21.9 (17.9)	21.8 (18)
Male sex, *N* (%)	75,684 (50.9)	7,618 (51.2)	60,484 (50.8)	7,578 (50.9)	7,692 (51.3)	60,375 (50.8)	7,617 (50.9)
DBDS, *N* (%)	69,885 (48.3)	7,134 (49.3)	55,581 (48.1)	7,162 (49.5)	7,170 (49.1)	55,617 (48.2)	7,098 (48.8)
Any cancer diagnosis, *N* (%)	48,998 (32.9)	4,976 (33.4)	39,401 (33.1)	4,618 (31)	4,914 (32.8)	39,347 (33.1)	4,737 (31.6)
Breast cancer diagnosis, *N* (%)	6,913 (12.3)	700 (12.4)	5,531 (12.3)	681 (11.9)	740 (12.1)	5,513 (12.4)	660 (11.5)
Colon cancer diagnosis, *N* (%)	6,303 (5.9)	614 (5.8)	5,076 (6)	612 (5.6)	630 (5.9)	5,055 (6)	618 (5.7)
Lung cancer diagnosis, *N* (%)	7,535 (7)	755 (7.1)	6,119 (7.1)	661 (6.1)	771 (7.1)	6,046 (7.1)	718 (6.6)
Prostate cancer diagnosis, *N* (%)	5,193 (9.3)	529 (9.6)	4,195 (9.5)	469 (8.2)	517 (9.2)	4,151 (9.4)	525 (9.2)

Demographic Overview of the CHB-CDC Cohort (*N* = 148,783), Stratified by Full-Cohort GWAS c-aAb PRS (Low, Intermediate, High).

Diagnosis: Earliest NPR registered date of relevant ICD10/8 code. Breast cancer and prostate cancer diagnoses N and % determined for CHB-CANCER women and men, respectively.

DBDS: Healthy blood donors from the DBDS cohort.

Low PRS: PRS < 10th percentile.

Intermediate PRS: PRS between the 10th and 90th percentiles.

High PRS: PRS > 90th percentile.

Supplementary table 4: CHB-CID cohort description							
	Full Cohort	IL-1α c-aAb Low PRS	IL-1α c-aAb Intermediate PRS	IL-1α c-aAb High PRS	IL-6 c-aAb Low PRS	IL-6 c-aAb Intermediate PRS	IL-6 c-aAb High PRS
Mean age at registry start, years (SD)	20.3 (16.9)	19.9 (16.8)	20.3 (16.9)	20.1 (17)	20.2 (16.8)	20.3 (16.9)	20.2 (17)
Male sex, *N* (%)	104,319 (48.4)	10,472 (48.6)	83,377 (48.4)	10,450 (48.5)	11,507 (49.1)	82,340 (48.3)	10,472 (48.3)
DBDS, *N* (%)	67,927 (31.5)	7,079 (32.9)	53,835 (31.2)	7,004 (32.5)	7,521 (32.1)	53,530 (31.4)	6,876 (31.7)
Asthma diagnosis, *N* (%)	17,819 (8.3)	1,868 (8.7)	14,291 (8.3)	1,652 (7.7)	1,801 (7.7)	14,343 (8.4)	1,675 (7.7)
COPD diagnosis, *N* (%)	22,730 (10.5)	2,145 (10)	18,331 (10.6)	2,254 (10.5)	2,465 (10.5)	17,965 (10.5)	2,300 (10.6)
Rheumatoid Arthritis diagnosis, *N* (%)	7,242 (3.4)	686 (3.2)	6,011 (3.5)	545 (2.5)	885 (3.8)	5,702 (3.3)	655 (3)

Demographic Overview of the CHB-CID Cohort (*N* = 215,512), Stratified by Full-Cohort GWAS c-aAb PRS (Low, Intermediate, High).
Diagnosis: Earliest NPR registered date of relevant ICD10/8 code.
DBDS: Healthy blood donors from the DBDS cohort.
Low PRS: PRS < 10th percentile.
Intermediate PRS: PRS between the 10th and 90th percentiles.
High PRS: PRS > 90th percentile.

Supplementary table 5: CHB-DEBD cohort description

	Full Cohort	IL-1α c-aAb Low PRS	IL-1α c-aAb Intermediate PRS	IL-1α c-aAb High PRS	IL-6 c-aAb Low PRS	IL-6 c-aAb Intermediate PRS	IL-6 c-aAb High PRS
Mean age at registry start, years (SD)	19.9 (16.7)	19.5 (16.5)	20 (16.7)	19.8 (16.8)	19.9 (16.6)	19.9 (16.7)	19.9 (16.7)
Male sex, *N* (%)	115,734 (48.7)	11,631 (49)	92,526 (48.7)	11,553 (48.7)	12,727 (49.2)	91,361 (48.7)	11,646 (48.7)
DBDS, *N* (%)	66,980 (28.2)	7,009 (29.5)	53,089 (27.9)	6,873 (28.9)	7,412 (28.7)	52,778 (28.1)	6,790 (28.4)
Atherosclerosis diagnosis, *N* (%)	9,950 (4.2)	1,052 (4.4)	7,887 (4.2)	1,011 (4.3)	1,021 (3.9)	7,867 (4.2)	1,062 (4.4)
Stroke diagnosis, *N* (%)	17,104 (7.2)	1,702 (7.2)	13,569 (7.1)	1,832 (7.7)	1,881 (7.3)	13,466 (7.2)	1,757 (7.4)

Demographic Overview of the CHB-DEBD Cohort (*N* = 237,444), Stratified by Full-Cohort GWAS c-aAb PRS (Low, Intermediate, High).

Diagnosis: Earliest NPR registered date of relevant ICD10/8 code.

DBDS: Healthy blood donors from the DBDS cohort.

Low PRS: PRS < 10th percentile.

Intermediate PRS: PRS between the 10th and 90th percentiles.

High PRS: PRS > 90th percentile.

Supplementary table 6: CHB-OCMS cohort description							
	Full Cohort	IL-1α c-aAb Low PRS	IL-1α c-aAb Intermediate PRS	IL-1α c-aAb High PRS	IL-6 c-aAb Low PRS	IL-6 c-aAb Intermediate PRS	IL-6 c-aAb High PRS
Mean age at registry start, years (SD)	16.8 (15.5)	16.4 (15.3)	16.8 (15.5)	16.7 (15.6)	16.8 (15.4)	16.8 (15.5)	16.7 (15.5)
Male sex, *N* (%)	116,925 (48.2)	11,755 (48.5)	93,485 (48.2)	11,658 (48.1)	12,890 (48.8)	92,263 (48.1)	11,772 (48.3)
DBDS, *N* (%)	66,922 (27.6)	7,021 (28.9)	53,033 (27.3)	6,859 (28.3)	7,402 (28)	52,742 (27.5)	6,778 (27.8)
Diabetes type 1 diagnosis, *N* (%)	86,96 (3.8)	957 (4.2)	6,799 (3.8)	939 (4.2)	819 (3.3)	6971 (3.9)	906 (4)
Diabetes type 2 diagnosis, *N* (%)	22,240 (9.3)	2,202 (9.2)	17,692 (9.2)	2,340 (9.8)	2,294 (8.8)	17,610 (9.3)	2,336 (9.7)

Demographic Overview of the CHB-OCMS Cohort (*N* = 242,599), Stratified by Full-Cohort GWAS c-aAb PRS (Low, Intermediate, High).
Diagnosis: Earliest NPR registered date of relevant ICD10/8 code.
DBDS: Healthy blood donors from the DBDS cohort.
Low PRS: PRS < 10th percentile.
Intermediate PRS: PRS between the 10th and 90th percentiles.
High PRS: PRS > 90th percentile.

Supplementary table 7: CHB-PDS cohort description							
	Full Cohort	IL-1α c-aAb Low PRS	IL-1α c-aAb Intermediate PRS	IL-1α c-aAb High PRS	IL-6 c-aAb Low PRS	IL-6 c-aAb Intermediate PRS	IL-6 c-aAb High PRS
Mean age at registry start, years (SD)	18.2 (16.4)	17.9 (16.2)	18.3 (16.4)	18.1 (16.5)	18.2 (16.4)	18.2 (16.4)	18 (16.4)
Male sex, *N* (%)	96,590 (47.5)	9,765 (48)	77,164 (47.5)	9,637 (47.4)	10,624 (47.9)	76,350 (47.5)	9,616 (47.2)
DBDS, *N* (%)	67,790 (33.3)	7,062 (34.7)	53,739 (33)	6,980 (34.3)	7,490 (33.7)	53,423 (33.2)	6,877 (33.8)
Back pain diagnosis, *N* (%)	12,992 (6.4)	1,333 (6.6)	10,323 (6.3)	1,332 (6.6)	1,391 (6.3)	10,303 (6.4)	1,298 (6.4)
Depression diagnosis, *N* (%)	17,781 (8.7)	1,640 (8.1)	14,380 (8.8)	1,754 (8.6)	1,907 (8.6)	14,063 (8.7)	1,811 (8.9)
Osteoarthritis diagnosis, *N* (%)	50,478 (24.8)	4,945 (24.3)	40,598 (25)	4,926 (24.2)	5,487 (24.7)	39,996 (24.9)	4,995 (24.5)

Demographic Overview of the CHB-PDS Cohort (*N*=203,320), Stratified by Full-Cohort GWAS c-aAb PRS (Low, Intermediate, High).
Diagnosis: Earliest NPR registered date of relevant ICD10/8 code.
DBDS: Healthy blood donors from the DBDS cohort.
Low PRS: PRS < 10th percentile.
Intermediate PRS: PRS between the 10th and 90th percentiles.
High PRS: PRS > 90th percentile.

Supplementary table 8: Utilized public software and packages	
Software	Availability
BWA 0.7.10 mem	https://github.com/lh3/bwa
DeepNull	https://doi.org/10.1101/2021.05.26.445783
FlashPCA v2.0	https://doi.org/10.1371/journal.pone.0093766
FUMA v1.6.6	https://fuma.ctglab.nl/
GenomeAnalysisTKLite 2.3.9	https://github.com/broadgsa/gatk/
GraphTyper v2.0-beta	https://github.com/DecodeGenetics/graphtyper
JASS v2.2	https://gitlab.pasteur.fr/statistical-genetics/jass
KING v2.3	https://kingrelatedness.com
LDSC v1.0.1	https://github.com/bulik/ldsc
Picard tools 1.117	https://broadinstitute.github.io/picard/
PLINK v2.0	https://doi.org/10.1186/s13742-015-0047-8
PRSice-2 v2.3.3	https://choishingwan.github.io/PRSice/
REGENIE v3.3	https://doi.org/10.1038/s41588-021-00870-7
SHAPEIT4 v4.2.2	https://odelaneau.github.io/shapeit4/
snakemake v7.18.2	https://snakemake.github.io
Variant Effect Predictor	https://github.com/Ensembl/ensembl-vep

All publicly available software and packages, including versions, utilized in this study.

Full list of DBS genomic consortium members and affiliations

Jakob Bay[16], Andrea Barghetti[1], Mette Skou Bentsen[1], Jens Kjærgaard Boldsen[14], Søren Brunak[17], Nanna Brøns[1], Alfonso Buil Demur[9], Johan Skov Bundgaard[1, 10], Lea Arregui Nordahl[16], Maria Didriksen[1], Khoa Manh Dinh[14], Joseph Dowsett[1], Christian Erikstrup[14, 15], Josephine Gladov[14, 15], Daniel Gudbjartsson[18], Thomas Folkmann Hansen[19], Dorte Helenius Mikkelsen[9], Lotte Hindhede[14], Henrik Hjalgrim[20, 21], Jakob Hjorth von Stemann[1], Bitten Aagaard Jensen[13], Kathrine Kaspersen[14], Bertram Dalskov Kjerulff[14], Lisette Kogelman[19], Mette Kongstad[1], Susan Mikkelsen[14], Christina Mikkelsen[1, 11], Line Hjorth Stjernholm Nielsen[14, 15], Janna Nissen[1] , Mette Nyegaard[22], Sisse Rye Ostrowski[1, 3], Frederikke Byron Pedersen[1], Ole Birger Pedersen[3, 16], Liam James Elgaard Quinn[16], Þórunn Rafnar[18], Palle Duun Rohde[22], Klaus Rostgaard[20, 21], Andrew J. Schork[9], Michael Schwinn[1], Erik Sørensen[1], Kari Stefansson[18, 23], Hreinn Stefansson[18], Jacob Træholt[1], Unnur Þorsteinsdóttir[18], Mie Topholm Bruun[12], Henrik Ullum[24], Thomas Werge[3, 8, 9]

[1]Department of Clinical Immunology, Copenhagen University Hospital- Rigshospitalet, Copenhagen, Denmark

[2] The DANBIO registry, the Danish Rheumatologic Biobank and Copenhagen Center for Arthritis Research (COPECARE), Centre for Rheumatology and Spine Diseases, Centre of Head and Orthopaedics, Copenhagen University Hospital - Rigshospitalet, Glostrup, Denmark

[3] Department of Clinical Medicine, Faculty of Health and Medical Sciences, University of Copenhagen, Copenhagen, Denmark

[4] Department of Oncology, Copenhagen University Hospital - Rigshospitalet, Copenhagen, Denmark

[5] Department of Clinical Biochemistry, Copenhagen University Hospital - Rigshospitalet, Copenhagen, Denmark

[6] Department of Respiratory Medicine, Copenhagen University Hospital - Bispebjerg, Copenhagen, Denmark

[7] Department of Respiratory Medicine, Copenhagen University Hospital - Hvidovre, Hvidovre, Denmark

[8] Section for Geogenetics, GLOBE Institute, Faculty of Health and Medical Sciences, Copenhagen University, Copenhagen, Denmark

[9] Institute of Biological Psychiatry, Mental Health Centre, Sct. Hans, Copenhagen University Hospital, Roskilde, Denmark

[10] Department of Cardiology, Copenhagen University Hospital – Rigshospitalet, Copenhagen, Denmark

[11] Novo Nordisk Foundation Center for Basic Metabolic Research, University of Copenhagen, Copenhagen, Denmark

[12] Clinical Immunology Research Unit, Department of Clinical Immunology, Odense University Hospital, Odense, Denmark

[13]Department of Clinical Immunology, Aalborg University Hospital, Aalborg, Denmark

[14] Department of Clinical Immunology, Aarhus University Hospital, Aarhus, Denmark

[15] Department of Clinical Medicine, Aarhus University, Aarhus, Denmark

[16] Department of Clinical Immunology, Zealand University Hospital, Køge, Denmark

[17] Novo Nordisk Foundation Center for Protein Research, Faculty of Health and Medical Sciences, University of Copenhagen, Copenhagen, Denmark
[18] deCODE genetics/Amgen Inc., Reykjavik, Iceland

[19] Danish Headache Center, Department of Neurology, Copenhagen University Hospital, Rigshospitalet - Glostrup, Copenhagen, Denmark

[20] Danish Cancer Society Research Center, Copenhagen, Denmark

[21] Department of Epidemiology Research, Statens Serum Institut, Copenhagen, Denmark

[22] Department of Health Science and Technology, Faculty of Medicine, Aalborg University, Aalborg, Denmark

[23] Faculty of Medicine, School of Health Sciences, University of Iceland, Reykjavik, Iceland

[24] Statens Serum Institut, Copenhagen, Denmark
