# Supplementary material for: Genetic architecture of cytokine autoantibodies and associated risk of common diseases
Source: Nat Commun. 2026 Aug 22;17:10053. doi: 10.1038/s41467-026-76394-7 (PMC13594223; doi:10.1038/s41467-026-76394-7)
Supplement: Supplementary file 2 [file 41467_2026_76394_MOESM2_ESM.pdf]

Description of Additional Supplementary Files

Genetic architecture of cytokine autoantibodies and associated risk of common diseases

File Name: Supplementary data 1

Description:

Lead Variants from IL-1α c-aAb Whole-Cohort GWAS with $0.1 < r^2 < 0.6$ and $p < 5 \times 10^{-8}$. Variant positions are based on the hg19 genomic build. GWAS performed with REGENIE (version 3.3) using a generalized linear mixed model approach.
Nearest Gene: Gene closest to the variant, determined by base pair distance and expected variant deleteriousness in cases of overlapping genes.
Variant Target: Specific functional region of the gene targeted by the variant.
CADD Score: An approximation of variant deleteriousness calculated using the FUMA platform. A CADD score ≥ 12.37 is considered pathogenic.
Validated Variants: GWAS-significant variants from the test cohort, validated in the validation cohort ($p < 0.05$, Bonferroni-Holm adjusted).

File Name: Supplementary data 2

Description:

Independent Variants from IL-1α c-aAb Whole-Cohort GWAS with $r^2 < 0.6$ and $p < 5 \times 10^{-8}$. Variant positions are based on the hg19 genomic build. GWAS performed with REGENIE (version 3.3) using a generalized linear mixed model approach.
Nearest Gene: Gene closest to the variant, determined by base pair distance and expected variant deleteriousness in cases of overlapping genes.
Variant Target: Specific functional region of the gene targeted by the variant.
CADD Score: An approximation of variant deleteriousness calculated using the FUMA platform. A CADD score ≥ 12.37 is considered pathogenic.
Validated Variants: GWAS-significant variants from the test cohort, validated in the validation cohort ($p < 0.05$, Bonferroni-Holm adjusted).

File Name: Supplementary data 3

Description:

Lead Variants from IL-6 c-aAb Whole-Cohort GWAS with $0.1 < r^2 <0.6$ and $p < 5 \times 10^{-8}$. Variant positions are based on the hg19 genomic build. GWAS performed with REGENIE (version 3.3) using a generalized linear mixed model approach.
Nearest Gene: Gene closest to the variant, determined by base pair distance and expected variant deleteriousness in cases of overlapping genes.
Variant Target: Specific functional region of the gene targeted by the variant.
CADD Score: An approximation of variant deleteriousness calculated using the FUMA platform. A CADD score ≥ 12.37 is considered pathogenic.
Validated Variants: GWAS-significant variants from the test cohort, validated in the validation cohort ($p < 0.05$, Bonferroni-Holm adjusted).

File Name: Supplementary data 4

Description:

Independent Variants from IL-6 c-aAb Whole-Cohort GWAS with $r^2 < 0.6$ and $p < 5 \times 10^{-8}$. Variant positions are based on the hg19 genomic build. GWAS performed with REGENIE (version 3.3) using a generalized linear mixed model approach.
Nearest Gene: Gene closest to the variant, determined by base pair distance and expected variant deleteriousness in cases of overlapping genes.
Variant Target: Specific functional region of the gene targeted by the variant.
CADD Score: An approximation of variant deleteriousness calculated using the FUMA platform. A CADD score ≥ 12.37 is considered pathogenic.
Validated Variants: GWAS-significant variants from the test cohort, validated in the validation cohort ($p < 0.05$, Bonferroni-Holm adjusted).

File Name: Supplementary data 5
Description:

Lead Variants from IL-10 c-aAb Whole-Cohort GWAS with $0.1 < r^2 < 0.6$ and $p < 5 \times 10^{-8}$. Variant positions are based on the hg19 genomic build. GWAS performed with REGENIE (version 3.3) using a generalized linear mixed model approach.
Nearest Gene: Gene closest to the variant, determined by base pair distance and expected variant deleteriousness in cases of overlapping genes.
Variant Target: Specific functional region of the gene targeted by the variant.
CADD Score: An approximation of variant deleteriousness calculated using the FUMA platform. A CADD score ≥ 12.37 is considered pathogenic.
Validated Variants: GWAS-significant variants from the test cohort, validated in the validation cohort ($p < 0.05$, Bonferroni-Holm adjusted).

File Name: Supplementary data 6

Description:

Independent Variants from IL-10 c-aAb Whole-Cohort GWAS with $r^2 < 0.6$ and $p < 5 \times 10^{-8}$. Variant positions are based on the hg19 genomic build. GWAS performed with REGENIE (version 3.3) using a generalized linear mixed model approach.
Nearest Gene: Gene closest to the variant, determined by base pair distance and expected variant deleteriousness in cases of overlapping genes.
Variant Target: Specific functional region of the gene targeted by the variant.
CADD Score: An approximation of variant deleteriousness calculated using the FUMA platform. A CADD score ≥ 12.37 is considered pathogenic.
Validated Variants: GWAS-significant variants from the test cohort, validated in the validation cohort ($p < 0.05$, Bonferroni-Holm adjusted).

File Name: Supplementary data 7
Description:

Lead Variants from GM-CSF c-aAb Whole-Cohort GWAS with $0.1 < r^2 < 0.6$ and $p < 5 \times 10^{-8}$. Variant positions are based on the hg19 genomic build. GWAS performed with REGENIE (version 3.3) using a generalized linear mixed model approach.
Nearest Gene: Gene closest to the variant, determined by base pair distance and expected variant deleteriousness in

cases of overlapping genes.
Variant Target: Specific functional region of the gene targeted by the variant.
CADD Score: An approximation of variant deleteriousness calculated using the FUMA platform. A CADD score ≥ 12.37 is considered pathogenic.
Validated Variants: GWAS-significant variants from the test cohort, validated in the validation cohort ($p < 0.05$, Bonferroni-Holm adjusted).

File Name: Supplementary data 8

Description:

Independent Variants from GM-CSF c-aAb Whole-Cohort GWAS with $r^2 < 0.6$ and $p < 5 \times 10^{-8}$. Variant positions are based on the hg19 genomic build. GWAS performed with REGENIE (version 3.3) using a generalized linear mixed model approach.
Nearest Gene: Gene closest to the variant, determined by base pair distance and expected variant deleteriousness in cases of overlapping genes.
Variant Target: Specific functional region of the gene targeted by the variant.
CADD Score: An approximation of variant deleteriousness calculated using the FUMA platform. A CADD score ≥ 12.37 is considered pathogenic.
Validated Variants: GWAS-significant variants from the test cohort, validated in the validation cohort ($p < 0.05$, Bonferroni-Holm adjusted).

File Name: Supplementary data 9

Description:

Significant genomic regions identified by multi-trait GWAS joint test analysis of c-aAb GWAS summary statistics (joint test $p < 2.91 \times 10^{-5}$, multiple testing adjusted by 1,703 genomic regions). Region start/stop positions correspond to hg19 genomic build. Joint effects were analyzed using an omnibus test based on a multivariate Wald statistic, equivalent to a two-sided test.
Pair (A x B): The specific c-aAb summary statistics paired for analysis.
Joint p value: p from JASS multi-trait GWAS test for corresponding c-aAb phenotype summary statistic pairing. Filtered for results above GWAS significant ($p < 5 \times 10^{-8}$).
z-score (A/B): JASS-computed regional z-scores for phenotypes A and B for the joint test.
Lead SNP (A/B): rsID of SNP with lowest p corresponding to the specific Region for univariate phenotype A/B test (corresponding to original GWAS results).
Min. p (A/B): p for lead SNP for the specific Region for univariate phenotype A/B test (corresponding to original GWAS results).
Interpretation: Verdict on whether results indicate a legitimately shared region (Both univariate p values $< 1 \times 10^{-5}$, shared z-score directionality), appear dominated by the GWAS of one c-aAb (one univariate test $p < 1 \times 10^{-5}$, other univariate $p > 1 \times 10^{-5}$, shared z-score directionality), or of opposite-direction pleiotropy (both univariate test p values $< 1 \times 10^{-5}$, opposite z-score directionality). The remaining results are unclassified.

File Name: Supplementary data 10

Description:

Positional and eQTL-Mapped Genes for IL-1α c-aAb, Identified in Whole-Cohort GWAS (hg19 Build), performed by FUMA
Pos. mapped SNPs (N): Number of variants mapped within a 100kb distance through positional mapping.
Pos. mapped Max CADD Score: Maximum CADD score for positionally mapped variants.
eQTL Mapped Minimum p: Minimum p for eQTL-mapped variants.
eQTL Mapped Minimum FDR: Minimum FDR for eQTL-mapped variants.
eQTL Direction: Directionality of eQTL variant mapping ("+" for increased gene expression, "-" for decreased expression).
Mapped SNP Minimum P-Value: Minimum GWAS p-value of positionally/eQTL-mapped variants.
Independent SNPs: List of significant independent variants with $p < 5 \times 10^{-8}$

File Name: Supplementary data 11

Description:

Protein-Coding Genes Identified for IL-1α c-aAb Based on Full Cohort GWAS Using MAGMA Gene-Based Tests. MAGMA aggregates variants within each gene using two-sided multiple linear regression, factoring in LD and individual p-values. Significant Genes were selected based on a p-value cutoff of 2.6×10^{-6}, Bonferroni-corrected for the number of protein-coding genes.

File Name: Supplementary data 12

Description:

Candidate Genes for IL-1α c-aAb Phenotype, Based on Full-Cohort GWAS, were selected based on fulfilling at least two of the following criteria: being the nearest gene to a lead variant, significant eQTL mapping, or MAGMA gene-based enrichment.

File Name: Supplementary data 13

Description:

Positional and eQTL-Mapped Genes for IL-6 c-aAb, Identified in Whole-Cohort GWAS (hg19 Build), performed by FUMA.
Pos. mapped SNPs (N): Number of variants mapped within a 100kb distance through positional mapping.
Pos. mapped Max CADD Score: Maximum CADD score for positionally mapped variants.
eQTL Mapped Minimum p: Minimum p for eQTL-mapped variants.
eQTL Mapped Minimum FDR: Minimum FDR for eQTL-mapped variants.
eQTL Direction: Directionality of eQTL variant mapping ("+" for increased gene expression, "-" for decreased expression).
Mapped SNP Minimum P-Value: Minimum GWAS p-value of positionally/eQTL-mapped variants.
Independent SNPs: List of significant independent variants with $p < 5 \times 10^{-8}$

File Name: Supplementary data 14

Description:

Protein-Coding Genes Identified for IL-6 c-aAb Based on Full Cohort GWAS Using MAGMA Gene-Based Tests. MAGMA aggregates variants within each gene using two-sided multiple linear regression, factoring in LD and individual p-values.

Significant Genes were selected based on a p-value cutoff of 2.6×10^{-6}, Bonferroni-corrected for the number of protein-coding genes.

File Name: Supplementary data 15

Description:

Candidate Genes for IL-6 c-aAb Phenotype, Based on Full-Cohort GWAS, were selected based on fulfilling at least two of the following criteria: being the nearest gene to a lead variant, significant eQTL mapping, or MAGMA gene-based enrichment.

File Name: Supplementary data 16

Description:

Positional and eQTL-Mapped Genes for IL-10 c-aAb, Identified in Whole-Cohort GWAS (hg19 Build), performed by FUMA.
Pos. mapped SNPs (N): Number of variants mapped within a 100kb distance through positional mapping.
Pos. mapped Max CADD Score: Maximum CADD score for positionally mapped variants.
eQTL Mapped Minimum p: Minimum p for eQTL-mapped variants.
eQTL Mapped Minimum FDR: Minimum FDR for eQTL-mapped variants.
eQTL Direction: Directionality of eQTL variant mapping ("+" for increased gene expression, "-" for decreased expression).
Mapped SNP Minimum P-Value: Minimum GWAS p-value of positionally/eQTL-mapped variants.
Independent SNPs: List of significant independent variants with $p < 5 \times 10^{-8}$

File Name: Supplementary data 17

Description:

Protein-Coding Genes Identified for IL-10 c-aAb Based on Full Cohort GWAS Using MAGMA Gene-Based Tests. MAGMA aggregates variants within each gene using two-sided multiple linear regression, factoring in LD and individual p values. Significant Genes were selected based on a p cutoff of 2.6×10^{-6}, Bonferroni-corrected for the number of protein-coding genes.

File Name: Supplementary data 18

Description:

Candidate Genes for IL-10 c-aAb Phenotype, Based on Full-Cohort GWAS, were selected based on fulfilling at least two of the following criteria: being the nearest gene to a lead variant, significant eQTL mapping, or MAGMA gene-based enrichment.

File Name: Supplementary data 19

Description:

Positional and eQTL-Mapped Genes for GM-CSF c-aAb, Identified in Whole-Cohort GWAS (hg19 Build), performed by FUMA.
Pos. mapped SNPs (N): Number of variants mapped within a 100kb distance through positional mapping.
Pos. mapped Max CADD Score: Maximum CADD score for positionally mapped variants.
eQTL Mapped Minimum p: Minimum p for eQTL-mapped variants.
eQTL Mapped Minimum FDR: Minimum FDR for eQTL-mapped variants.
eQTL Direction: Directionality of eQTL variant mapping ("+" for increased gene expression, "-" for decreased expression).
Mapped SNP Minimum P-Value: Minimum GWAS p-value of positionally/eQTL-mapped variants.
Independent SNPs: List of significant independent variants with $p < 5 \times 10^{-8}$

File Name: Supplementary data 20

Description:

Candidate Genes for GM-CSF c-aAb Phenotype, Based on Full-Cohort GWAS, were selected based on fulfilling at least two of the following criteria: being the nearest gene to a lead variant, significant eQTL mapping, or MAGMA gene-based enrichment.

File Name: Supplementary data 21

Description:

Statistically Significant Gene Sets from GTEx V8 (54 Tissue Types), Based on IL-1α c-aAb whole-cohort GWAS Candidate Genes. Gene sets were identified using FUMA's GENE2FUNC tool, which performs one-sided Fisher's exact tests for overrepresentation within predefined gene sets.
Adjusted p-values: Benjamini-Hochberg FDR multiple test correction applied.

File Name: Supplementary data 22

Description:

Statistically Significant Gene Sets from GTEx V8 (54 Tissue Types), Based on IL-10 c-aAb whole-cohort GWAS Candidate Genes. Gene sets were identified using FUMA's GENE2FUNC tool, which performs one-sided Fisher's exact tests for overrepresentation within predefined gene sets.
Adjusted p-values: Benjamini-Hochberg FDR multiple test correction applied.

File Name: Supplementary data 23

Description:

Shared Candidate Genes for the IL-1α c-aAb Phenotype, Based on Sex-Specific GWAS Tests. Candidate genes were identified based on overlap of at least two of the following: being the nearest gene to a lead variant, significant eQTL mapping, or MAGMA gene-based enrichment.
W: Candidate gene for female-specific GWAS output.
M: Candidate gene for male-specific GWAS output.

W/M: Candidate gene for both female and male-specific GWAS outputs.

File Name: Supplementary data 24

Description:

Sex-Specific Candidate Genes for the IL-1α c-aAb Phenotype, Based on Sex-Specific GWAS Tests. Candidate genes were identified based on overlap of at least two of the following: being the nearest gene to a lead variant, significant eQTL mapping, or MAGMA gene-based enrichment.
W: Candidate gene for female-specific GWAS output.
M: Candidate gene for male-specific GWAS output.
W/M: Candidate gene for both female and male-specific GWAS outputs.

File Name: Supplementary data 25

Description:

SNPs included in the PRS were selected from the IL-1α c-aAb whole-cohort GWAS using an LD clumping threshold of $r^2 < 0.1$ and a GWAS p value threshold of $p < 5 \times 10^{-8}$, as implemented in PRSice-2. PRS associations with c-aAb were evaluated using linear regression models with two-sided Wald tests. The best-fit PRS was defined as the GWAS p-value threshold yielding the highest Nagelkerke's r^2. Permutation-derived empirical p-values were computed to account for multiple testing across PRS p-value thresholds, yielding an IL-1α c-aAb whole-cohort PRS with a best-fit model p of 2.51×10^{-34} and empirical p of 0.000999.
Nearest Gene: Gene closest to the variant, determined by base pair distance.
Variant Target: Specific functional region of the gene targeted by the variant.

File Name: Supplementary data 26

Description:

SNPs included in the PRS were selected from the IL-6 c-aAb whole-cohort GWAS using an LD clumping threshold of $r^2 < 0.1$ and a GWAS p value threshold of $p < 5 \times 10^{-8}$, as implemented in PRSice-2. PRS associations with c-aAb were evaluated using linear regression models with two-sided Wald tests. The best-fit PRS was defined as the GWAS p value threshold yielding the highest Nagelkerke's r^2. Permutation-derived empirical p values were computed to account for multiple testing across PRS p value thresholds, yielding an IL-6 c-aAb whole-cohort PRS with a best-fit model p of 2.599×10^{-8} and empirical p of 0.000999.
Nearest Gene: Gene closest to the variant, determined by base pair distance.
Variant Target: Specific functional region of the gene targeted by the variant.

File Name: Supplementary data 27

Description:

SNPs included in the PRS were selected from the IL-10 c-aAb whole-cohort GWAS using an LD clumping threshold of $r^2 < 0.1$ and a GWAS p value threshold of $p < 5 \times 10^{-8}$, as implemented in PRSice-2. PRS associations with c-aAb were evaluated using linear regression models with two-sided Wald tests. The best-fit PRS was defined as the GWAS p value threshold yielding the highest Nagelkerke's r^2. Permutation-derived empirical p values were computed to account for

multiple testing across PRS p value thresholds, yielding an IL-10 c-aAb whole-cohort PRS with a best-fit model p of 1.14 $\times 10^{-5}$ and empirical p of 0.000999.
Nearest Gene: Gene closest to the variant, determined by base pair distance.
Variant Target: Specific functional region of the gene targeted by the variant.

File Name: Supplementary data 28

Description:

SNPs included in the PRS were selected from the IL-1α c-aAb male specific GWAS using an LD clumping threshold of $r^2 < 0.1$ and a GWAS p value threshold of $p < 5 \times 10^{-8}$, as implemented in PRSice-2. PRS associations with c-aAb were evaluated using linear regression models with two-sided Wald tests. The best-fit PRS was defined as the GWAS p value threshold yielding the highest Nagelkerke's r^2. Permutation-derived empirical p-values were computed to account for multiple testing across PRS p value thresholds, yielding an IL-1α c-aAb male-specific cohort PRS with a best-fit model p of 1.75 $\times 10^{-18}$ and empirical p of 0.000999.
Nearest Gene: Gene closest to the variant, determined by base pair distance.
Variant Target: Specific functional region of the gene targeted by the variant.

File Name: Supplementary data 29

Description:

SNPs included in the PRS were selected from the IL-1α c-aAb female specific GWAS using an LD clumping threshold of $r^2 < 0.1$ and a GWAS p value threshold of $p < 5 \times 10^{-8}$, as implemented in PRSice-2. PRS associations with c-aAb were evaluated using linear regression models with two-sided Wald tests. The best-fit PRS was defined as the GWAS p value threshold yielding the highest Nagelkerke's r^2. Permutation-derived empirical p values were computed to account for multiple testing across PRS p value thresholds, yielding an IL-1α c-aAb female-specific cohort PRS with a best-fit model p of 6.12 $\times 10^{-16}$ and empirical p of 0.000999.
Nearest Gene: Gene closest to the variant, determined by base pair distance.
Variant Target: Specific functional region of the gene targeted by the variant.

File Name: Supplementary data 30

Description:

SNPs included in the PRS were selected from the IL-1α c-aAb "High" vs "Low" titer GWAS using an LD clumping threshold of $r^2 < 0.1$ and a GWAS p value threshold of $p < 5 \times 10^{-8}$, as implemented in PRSice-2. "High titers" defined as MFI>90th percentile, "low titers" as MFI<75th percentile. PRS associations with high or low c-aAb titers were evaluated using logistic regression models with two-sided Wald tests. The best-fit PRS was defined as the GWAS p value threshold yielding the highest Nagelkerke's r^2. Permutation-derived empirical p-values were computed to account for multiple testing across PRS p value thresholds, yielding an IL-1α c-aAb whole cohort "High vs Low titer" PRS with a best-fit model p of 6.12 $\times 10^{-16}$ and empirical p of 0.000999.
Nearest Gene: Gene closest to the variant, determined by base pair distance.
Variant Target: Specific functional region of the gene targeted by the variant.

File Name: Supplementary data 31

Description:

ICD10 and ICD8 codes defining investigated clinical outcomes in CHB cohort. ICD10 and 8 codes taken from the Danish National Patient registry (NPR). Earliest registered diagnosis date was used to define clinical outcome. CHB-cohort: The specific CHB sub-cohort used for the PRS-outcome association test.
