# Supplementary material for: Genetic architecture of cytokine autoantibodies and associated risk of common diseases
Source: Nat Commun. 2026 Aug 22;17:10053. doi: 10.1038/s41467-026-76394-7 (PMC13594223; doi:10.1038/s41467-026-76394-7)
Supplement: Supplementary file 4 [file 41467_2026_76394_MOESM5_ESM.pdf]

nature portfolio

Peer Review File

Genetic architecture of cytokine autoantibodies and associated risk of common diseases

Corresponding Author: Mr Jakob Hjorth von Stemann

This file contains all reviewer reports in order by version, followed by all author rebuttals in order by version.

Version 0:

Reviewer comments:

Reviewer #1

(Remarks to the Author)
The authors make an important contribution to human genetics by generating and validating the first polygenic risk scores (PRS) for cytokine autoantibodies (c-Abs), showing predictive value for both measured cytokine titers and relevant biobank-derived disease outcomes. This represents a valuable first step toward integrating c-Ab genetics into clinical and epidemiological research. The PRS development and validation are well executed, and the results convincingly demonstrate that these scores capture meaningful biological variation. Nevertheless:

1. PRS methodology – The analysis could be strengthened by comparing multiple PRS inference methods and leveraging genome-wide data rather than relying solely on p-value thresholding. I recommend, for example, the use of SBayesRC (Zheng et al., 2024, Nat Genet), a state-of-the-art approach that incorporates all available SNPs from GWAS.
2. Downstream GWAS interpretation – The authors' gene prioritization and gene-set enrichment analyses are a reasonable starting point for biological interpretation. However, the overwhelming concentration of genome-wide significant signals in the HLA region raises a key limitation: commonly used SNP-to-gene mapping strategies (e.g., eQTL-based mapping, as applied here) have not been systematically validated in the MHC region, where extended LD, extreme polymorphism, and mapping biases may yield misleading results. Specific gene-level claims for the HLA region should therefore be treated cautiously and ideally validated with dedicated analyses:
a. Non-HLA focus – To assess critical tissues genome-wide, Stratified-LD Score Regression (S-LDSC; Finucane et al., 2015, Nat Genet) would be appropriate, as it does not require prior definition of critical genes. While S-LDSC excludes the HLA region by default, it could still be applied to the remaining genome to evaluate tissue-specific enrichment outside the MHC. Although this removes the strongest contributors in the current GWAS, it could still detect enrichment signals in non-HLA loci.
b. Non-HLA focus – Such an analysis would also provide heritability estimates outside the HLA region, which could inform whether, with increased power, one might expect to detect significant non-HLA associations with c-Abs.
c. HLA focus – Defining "critical" genes via SNP thresholding in the HLA is problematic. Fine-mapping is also challenging in this region, but it remains a more rigorous alternative. I recommend multiple (or single) causal variant fine-mapping using methods such as SuSiE (Wang et al., 2020, JRSS Series B), restricting interpretation to high-confidence variants (e.g., PIP > 0.9).
d. HLA focus – The tissue enrichment analysis (Fig. 5) is performed marginally for each tissue, meaning tissues driven by the same genes appear similarly enriched. A more informative approach would be conditional analysis: (i) identify the most significant tissue; (ii) remove or down-weight overlapping genes contributing to its enrichment; (iii) recompute enrichment p-values for the remaining tissues; (iv) iterate until no tissues remain significant.
e. HLA focus – Ideally, a multivariate logistic regression could be used for 2d), where the response variable indicates whether a gene is "critical" and predictors represent tissue DEG status (n = 54), potentially with regularization to account for collinearity. However, sample size may limit feasibility.
f. HLA focus – A natural extension, given that each cytokine is analyzed separately, would be HLA-focused, cytokine-specific analyses that incorporate peptide-binding predictions. For each cytokine, one could identify candidate HLA alleles and assess predicted binding affinities to known cytokine-derived peptides using in-silico tools such as NetMHCIIpan (Karosiene et al., 2014, Immunogenetics).
3. Shared vs heterogeneous architecture – Because each cytokine was analyzed independently, the authors could explore shared versus distinct genetic architecture using multi-trait GWAS approaches (e.g., Hanna et al., 2020, NAR Genom Bioinform). Such analyses may (i) boost discovery for loci with shared effects, and (ii) highlight loci with heterogeneous or

even opposite effect directions across cytokines.
4. PRS–disease associations – While the survival analyses in Fig. 6 demonstrate predictive value for certain outcomes, it would be equally, if not more, informative to examine the genetic correlation between c-Ab GWAS and a wide panel of complex traits/diseases. This would help determine whether PRS–disease links reflect broad polygenic overlap or are driven by a small subset of loci.
5. Data availability – Could the authors clarify what type of data will be released? While I understand that raw individual-level data may not be publicly available, will full GWAS summary statistics be provided? Will PRS weights be made accessible for replication and downstream use?

Reviewer #2

(Remarks to the Author)

von Stemann et al have produced a very important and exciting paper identifying genomic foundations for some of the most interesting anti-cytokine autoantibodies. Their approach is valuable and robust and their findings provocative.

1. Were any of the antibodies tested for neutralizing activity? Were they tested on more than one occasion and if so were the results averaged? Could any of these been activating antibodies rather than inhibitory ones?

2. It is very reasonable to have run this analysis on the most homogeneous sample possible. However, were other ethnicities available and explored? This is particularly of interest with the IFNg-HLA story from Southeast Asia.

3. Did any of these antibody levels correlate with COVID exposure?

Reviewer #3

(Remarks to the Author)
Major Comments
This study represents a rare and valuable opportunity to analyse anti-cytokine autoantibodies in a very large, population-based cohort. The unbiased GWAS approach and agnostic binding assays are important strengths. Although the HLA associations are presented as novel, similar links between HLA alleles and autoantibody development have been reported previously and consistently. I suggest the authors temper their claims of novelty and instead highlight how their work complements or extends prior studies.

1. It would be helpful if the authors could provide, in the tables, the absolute numbers of individuals who were positive and negative for each anti-cytokine autoantibody in both the discovery and validation cohorts. This would allow readers to better gauge the prevalence and interpret the robustness of the genetic associations.

2. Because the assays used here detect only binding, the functionality of the detected autoantibodies is unknown. The clinical relevance of anti-cytokine autoantibodies usually hinges on whether they are neutralizing (e.g., anti–IFN-γ, anti–GM-CSF) or enhancing cytokine stability (e.g., anti–IL-6). Without functional data, the implications of detecting a binding antibody at a single time point are less clear. Non-neutralizing or transient antibodies may dilute the signal from high-titer, pathogenic neutralizing antibodies, potentially obscuring associations.

3. It is striking that strong HLA signals were found for disease-protective antibodies such as anti–IL-1a, whereas for pathogenic neutralizing autoantibodies (anti–IFN-γ, anti–GM-CSF), associations were weaker than in prior studies. This may reflect limited numbers of individuals with truly pathogenic autoantibodies anti-IFN-γ and anti–GM-CSF in the presumably mainly Caucasian cohort, or the possibility that binding-based assays captured non-pathogenic low-titer entities. Providing details on the distribution of specificities, titers, age-associations and overlap with known disease-associated phenotypes e.g. pulmonary alveolar proteinosis would clarify interpretation.

Minor Comments
1. Balance between statistics and biology
The manuscript is heavily weighted toward statistical reporting. It would be helpful to include more context on the biological and clinical implications of the findings. For instance:
• Which genes were included in the GWAS-based polygenic risk scores?
• What percentages of blood donors fell into each age category or percentile?
• Were there demographic patterns (e.g., sex, age) that might explain variation in autoantibody prevalence, e.g. if the blood donors were mainly 20-40 year-old and women then the expected prevalence of neutralizing anti-IFN-alpha autoantibodies in the cohort would be low since it was the reported prevalence of pathogenic anti-IFN-alpha autoantibodies was <1% in European cohorts aged <70 but increased to 2-6% in >70, and were higher in men.

2. The first sentence in the abstract should be plural "Anti-cytokine autoantibodies (c-aAb) are immunomodulatory phenomena linked to the risk and severity of various diseases".

3. Could the authors speculate on the clinical significance of MICC (MHC Class I Polypeptide-Related Sequence C) and IFNA10 (Interferon alpha-10) associations with anti-IL-10, since this is the only non-inflammatory cytokine studied in their panel?

Version 1:

Reviewer comments:

Reviewer #1

(Remarks to the Author)
I believe the authors have satisfactorily addressed most of my questions. I have only one remaining comment regarding their response to my suggestion about computing genetic correlations. My original motivation for this request was to better understand whether the genetics of autoantibody production could be causally linked to the phenotypes with which the authors report associations. While pleiotropy can arise simply by chance (given the limited number of genes in the genome)it may also reflect shared underlying biological mechanisms. However, I recognize that such analyses are beyond the scope of the present study, and I am not requesting additional work along these lines.

I would also encourage the authors to make an effort to provide easy access to the GWAS summary statistics generated in this study, as this would greatly facilitate transparency, reproducibility, and downstream use by the community.

Reviewer #2

(Remarks to the Author)
The authors have made successful efforts to respond to reviewer comments.

Please note that the ethnic association for anti-IFNg autoantibodies is South East Asian, not South Asian (page 22 bottom of marked MSS).

Reviewer #3

(Remarks to the Author)
It has been a pleasure to review the revised version of this manuscript. I commend the authors on the substantial improvements made to the work. The addition of noteworthy clinical information—specifically the longitudinal data demonstrating the stability of high-titer cytokine autoantibodies over a decade, the nuances of threshold setting to find shared and distinct associations in females and males and the breakdown of the PRS —provides significant depth to the study and better supports the authors' interpretations. The revisions have effectively addressed nearly all of my previous concerns, particularly:
1. The inclusion of heritability estimates outside the MHC region, which clarifies the concentration of genetic contributors within the HLA.
2. The multi-trait GWAS analyses, which offer valuable insights into the shared versus distinct genetic architectures of the various cytokines.
3. The increased transparency regarding the specific genes utilized in the polygenic risk score.
4. The thoughtful expansion of the Discussion to account for demographic and ancestry-related influences on autoantibody prevalence.

I have only one remaining minor request, for the purpose of complete transparency. While you have clearly defined the thresholds for the binary high/low titer c-aAb phenotypes (MFI >90 percentile for high-titer cases and MFI <75 percentile for low-titer controls), the absolute numbers of individuals classified into these categories for each cytokine are not explicitly stated. Could the absolute number of individuals classed as binary high/low titer for each cytokine be added to Supplementary Table 1? Providing these counts will allow readers to better interpret the robustness of the genetic associations and the prevalence of these phenotypes within your discovery and validation cohorts.

I believe the manuscript will be an excellent contribution to the field.

Open Access This Peer Review File is licensed under a Creative Commons Attribution 4.0 International License, which permits use, sharing, adaptation, distribution and reproduction in any medium or format, as long as you give appropriate credit to the original author(s) and the source, provide a link to the Creative Commons license, and indicate if changes were made.
In cases where reviewers are anonymous, credit should be given to 'Anonymous Referee' and the source.
The images or other third party material in this Peer Review File are included in the article's Creative Commons license, unless indicated otherwise in a credit line to the material. If material is not included in the article's Creative Commons license and your intended use is not permitted by statutory regulation or exceeds the permitted use, you will need to obtain permission directly from the copyright holder.
To view a copy of this license, visit https://creativecommons.org/licenses/by/4.0/

REVIEWER COMMENTS

Reviewer #1 (Remarks to the Author):

The authors make an important contribution to human genetics by generating and validating the first polygenic risk scores (PRS) for cytokine autoantibodies (c-Abs), showing predictive value for both measured cytokine titers and relevant biobank-derived disease outcomes. This represents a valuable first step toward integrating c-Ab genetics into clinical and epidemiological research. The PRS development and validation are well executed, and the results convincingly demonstrate that these scores capture meaningful biological variation. Nevertheless:

1. PRS methodology – The analysis could be strengthened by comparing multiple PRS inference methods and leveraging genome-wide data rather than relying solely on p-value thresholding. I recommend, for example, the use of SBayesRC (Zheng et al., 2024, Nat Genet), a state-of-the-art approach that incorporates all available SNPs from GWAS.

We thank the reviewer for this comment. This is an interesting suggestion, and for most traits we would agree that an alternate method could result in a gain in prediction. However, in our case, we do not believe the extra computational complexity is necessary.

Our primary aim regarding PRSs in this paper was to produce a genetic predictor of cytokine autoantibodies that can detect phenotype associations with disease outcomes, rather than optimal, clinical grade PRS for the cytokine autoantibodies, themselves. For this goal, our sample size is large enough that we judge that a potential modest gain in the sensitivity of the PRS (~1.15 times more variance as judged in Zheng et al 2024 (PMID: 38689000)) would not qualitatively improve our ability to detect these disease associations.

Further, SBayesRC and other shrinkage-based methods may not be optimal for the genetic architecture of our cytokine autoantibody phenotypes. All shrinkage-based (i.e., Bayesian) methods will tend to downweigh large effects if they are outliers relative to expected prior distribution. This is suitable, and even beneficial, when the architecture is highly polygenic, consisting of many SNPs of more modest statistical significance, but it does involve some "sacrificing" or "down-weighting" of outlying effects. However, when a GWAS is sufficiently powered to detect the phenotypes genetic architecture, this gain in efficiency is no longer necessary and a simple PRS can achieve optimal performance.

To this end, when we use PRSice, the best fitting PRS for all statistically significant c-aAb PRSs had genome-wide significant thresholds of p-value < 5 × 10^{-8} (see the table 2 PRS overview). This is reflective of an architecture that is dominated by few, highly prominent variants for the phenotype, as opposed to the more complex/polygenic phenotypes that may motivate shrinkage based PRSs. For a scenario that would motivate the use of shrinkage-based PRS, we would have expected qualitative improvement in prediction by continuously lowering the inclusion P-value threshold, which we did not observe. This is further corroborated by additional heritability and multi-trait GWAS tests performed for this revision, following the reviewers' suggestions. LD score regression-based heritability estimation of our c-aAb phenotypes, which excludes the HLA region by default, did not detect significant heritability of the traits (although, some point estimates were modest, see the comment 2.b response below), suggesting weak evidence of non-HLA driven polygenic signal. Likewise, candidates for shared, polygenic regions for c-aAb identified by multi-GWAS analyses were predominantly tied to the HLA region (see the comment 3 response below).

So, both in practice (i.e. our goals are associations) and in principle (which method suits our data best), we do not believe the extra complexity of shrinkage-based estimators is justified. However, we remain open to future analyses using shrinkage-methods in sensitivity analyses.

2. Downstream GWAS interpretation – The authors' gene prioritization and gene-set enrichment analyses are a reasonable starting point for biological interpretation. However, the overwhelming concentration of genome-wide significant signals in the HLA region raises a key limitation: commonly used SNP-to-gene mapping strategies (e.g., eQTL-based mapping, as applied here) have not been systematically validated in the MHC region, where extended LD, extreme polymorphism, and mapping biases may yield misleading results. Specific gene-level claims for the HLA region should therefore be treated cautiously and ideally validated with dedicated analyses:

a. Non-HLA focus – To assess critical tissues genome-wide, Stratified-LD Score Regression (S-LDSC; Finucane et al., 2015, Nat Genet) would be appropriate, as it does not require prior definition of critical genes. While S-LDSC excludes the HLA region by default, it could still be applied to the remaining genome to evaluate tissue-specific enrichment outside the MHC. Although this removes the strongest contributors in the current GWAS, it could still detect enrichment signals in non-HLA loci.

Using the default settings of the Bulik- developed LDSC pipeline, and the tissue groupings defined for "Multi-tissue gene expression" analyses in Finucane et al 2018 (PMID: 29632380), we were able to test for tissue specific enrichment in our c-aAb summary statistics results using the suggested method. We observed that for our GWAS summary statistics, the "immune/blood" category was enriched at nominal statistical significance ($0.01 < p\text{-value} < 0.05$) for IL-1α, IL-10, GM-CSF and IFNβ c-aAb). These findings, from HLA-excluded analyses, reflect the results in our preexisting enrichment data, which also highlighted enrichment in immune-relevant genes. However, none of the observed enrichment results remained significant when adjusting for multiple testing. We thank the reviewer for suggesting this analysis and have added these observations and our use of the method to the manuscript text.

b. Non-HLA focus – Such an analysis would also provide heritability estimates outside the HLA region, which could inform whether, with increased power, one might expect to detect significant non-HLA associations with c-Abs.

Including heritability estimates for our phenotypes is another excellent suggestion, which we have now added to our manuscript. Following our S-LDSC analyses outlined in the prior response, we used the Bulik et al LDSC pipeline to estimate heritability for our c-aAb summary statistics.

We obtained the following results for observed h^2 (standard error): IL-1α c-aAb $h^2 = 0.1472$ (0.086), IL-6 c-aAb $h^2 = 0.0695$ (0.099), IL-10 c-aAb $h^2 = 0.1851$ (0.14), GM-CSF c-aAb $h^2 = 0.0199$ (0.1068), IFNα c-aAb $h^2 = 0.0302$ (0.0925), IFNβ c-aAb $h^2 = 0.3049$ (0.1743) and IFNγ c-aAb $h^2 = 0.03$ (0.1566).

The size of the Standard Errors for these estimates indicates that beyond the HLA region (which is excluded by default in the Bulik/LDSC pipeline), there is limited evidence for heritability for c-aAb, at present. However, we acknowledge the reviewers' point, that higher sample sizes can uncover further genetic c-aAb predictors. The results and discussion sections of the manuscript have been updated to reflect this.

c. HLA focus – Defining "critical" genes via SNP thresholding in the HLA is problematic. Fine-mapping is also challenging in this region, but it remains a more rigorous alternative. I recommend multiple (or single) causal variant fine-mapping using methods such as SuSiE (Wang et al., 2020, JRSS Series B), restricting interpretation to high-confidence variants (e.g., PIP > 0.9).

We thank the reviewer for the suggested fine-mapping alternative for gene prioritization, which we agree would be the gold standard for causal gene isolation. Our aim in this study was to identify shortlists of potentially causal genes for our novel biomarker phenotypes rather than claiming definitive mechanistic genes. This, plus practical reasons (computational resources, reference panel availability) led to our more pragmatic criteria for isolating "candidate genes" (through proximity to lead GWAS variant, eQTL mapping and statistical significance in the MAGMA gene-based test), based on precedence for these prioritization criteria in current, high-profile GWAS publications including Jurgens et al 2024 (PMID: 39572784), Burns et al 2024 (PMID: 39543113), Watanabe et al 2017 (PMID: 29184056) and Kiryluk et al 2023 (PMID: 37337107). We have added more references to support this approach to the manuscript.

That said, we acknowledge the limitations of our approach (particularly in regard to the HLA region) and have elaborated in the discussion that highlighted genes in the HLA region should be regarded as "plausible candidates" rather than definitively causal for c-aAb. We have also made a clear note of the SuSiE method for causal-variant fine mapping for future studies.

d. HLA focus – The tissue enrichment analysis (Fig. 5) is performed marginally for each tissue, meaning tissues driven by the same genes appear similarly enriched. A more informative approach would be conditional analysis: (i) identify the most significant tissue; (ii) remove or down-weight overlapping genes contributing to its enrichment; (iii) recompute enrichment p-values for the remaining tissues; (iv) iterate until no tissues remain significant.

This is another excellent suggestion, which we implemented for our IL-1α c-aAb tissue enrichment analyses, the only case of statistically significant tissue enrichment results. When removing overlapping genes for the most significantly enriched tissue ("Brain Anterior cingulate cortex BA24", P-value =3.58×10^{-8}) across all three tests (one sided up/downregulated differential gene expression (DEG), two sided DEG), no significant tissues remained for any of the three tests. This shows that the tissue enrichment results are not independent, but rather a result of shared, enriched genes. A note has been added regarding this to the manuscript, as well as description of the conditional analysis approach. Considering this finding, as well as a call from the editor to change the figure, we have chosen to remove fig.5 from the manuscript.

e. HLA focus – Ideally, a multivariate logistic regression could be used for 2d), where the response variable indicates whether a gene is "critical" and predictors represent tissue DEG status (n = 54), potentially with regularization to account for collinearity. However, sample size may limit feasibility.

This would certainly be the most rigorous approach but is indeed being limited by the <100 candidate gene sample sizes in our case. Furthermore, we feel the results of our conditional analysis outlined in the 2.e response above sufficiently demonstrate the shared enrichment across the tissues.

f. HLA focus – A natural extension, given that each cytokine is analyzed separately, would be HLA-focused, cytokine-specific analyses that incorporate peptide-binding predictions. For each cytokine, one could identify candidate HLA alleles and assess predicted binding affinities to known cytokine-derived peptides using in-silico tools such as NetMHCIIpan (Karosiene et al., 2014, Immunogenetics).

While this is an intriguing suggestion, we consider it beyond the scope of this article, where our aim is to provide a broad overview of genetic predictors of several c-aAb, as well as potential links to major societal diseases. However, we have made note of the method for future reference, as it seems ideal for narrower studies of individual c-aAb etiology, through the structural/mechanistic interactions of target cytokines and HLA.

3. Shared vs heterogeneous architecture – Because each cytokine was analyzed independently, the authors could explore shared versus distinct genetic architecture using multi-trait GWAS approaches (e.g., Hanna et al., 2020, NAR Genom Bioinform). Such analyses may (i) boost discovery for loci with shared effects, and (ii) highlight loci with heterogeneous or even opposite effect directions across cytokines.

Another highly relevant analysis, which we implemented for all possible pairwise combinations of our c-aAb summary statistics data.

While the bulk of significant joint-test results appeared to be carried by one of the two tested c-aAb, we identified several potentially shared loci between IL-1α, IL-6, IL-10 and GM-CSF c-aAb - i.e. significant results for loci in Joint and Univariate analyses, and shared effect direction. These were nearly universally located in the HLA region. Depending on the c-aAb pairing opposing effects could also be observed for these loci. All other significant results appeared driven by one specific c-aAb trait.

These findings further suggest that while some c-aAb may have shared genetic regions, c-aAb of different specificities appear to have distinct genetic architectures, -at least for the presently used cohorts, and thresholds used for defining "shared", "opposite" or "trait dominated" regions.
The methods, results and discussion sections of this paper have been updated to include the multi-trait GWAS method, and the results added as a new supplementary table 11.

4. PRS–disease associations – While the survival analyses in Fig. 6 demonstrate predictive value for certain outcomes, it would be equally, if not more, informative to examine the genetic correlation between c-Ab GWAS and a wide panel of complex traits/diseases. This would help determine whether PRS–disease links reflect broad polygenic overlap or are driven by a small subset of loci.

Regarding the incorporation of a wider range of traits and diseases into the study, while there is no shortage of c-aAb related phenotypes, our aim in this paper was only to test for a few associations between c-aAb genetic predictors and major, common diseases that we had power to investigate in our cohorts. These included analyses of diseases with established c-aAb ties in the literature, such as diabetes and rheumatoid arthritis, substantiating our findings. We thus consider wider phenotype correlation studies beyond the scope of this study, where we also sought to specifically leverage our available, comparatively homogenous local populations (DBDS, CHB) for our analyses if possible. This also allowed us strict control of phenotype definitions through diagnosis codes from the Danish National Patient Registry, avoiding potential heterogeneity introduced by using external definitions for disease phenotypes. Furthermore, the present registry-based approach allowed us to incorporate temporal information into our study in the form of survival analyses.

However, we are always interested in testing c-aAb against specific phenotypes the reviewer may suggest and invite other researchers to use the public availability of our GWAS and PRS data for such studies.

5. Data availability – Could the authors clarify what type of data will be released? While I understand that raw individual-level data may not be publicly available, will full GWAS summary statistics be provided? Will PRS weights be made accessible for replication and downstream use?

Variants informing PRS calculation has been added as supplementary tables 27-32. GWAS data will be available upon request to DBDS steering committee member and director of CHB Sisse Rye Ostrowski. A note of this has been added to the manuscript text as well.

Reviewer #2 (Remarks to the Author):

von Stemann et al have produced a very important and exciting paper identifying genomic foundations for some of the most interesting anti-cytokine autoantibodies. Their approach is valuable and robust and their findings provocative.

1. Were any of the antibodies tested for neutralizing activity? Were they tested on more than one occasion and if so were the results averaged? Could any of these been activating antibodies rather than inhibitory ones?

Regarding c-aAb having neutralizing activity:

While we did not test for neutralizing ability within this study cohort (which would have been logistically unfeasible), several independent studies of the Danish blood donor cohort have confirmed that c-aAb from healthy donors targeting IFNα, IL-1α, IL-6, and IL-10 may be neutralizing in vitro, and that the neutralizing activity correlates very precisely with immunometric antibody activity measurements judged by ELISA, RIA and Luminex. For In Vitro neutralization studies for individual c-aAb from Danish blood donors, we refer to the following:

- ***For Type 1 Interferon c-aAb, see Hansen & Ross 1990 (PMID: 1690246), and Ross et al 1990 (PMID: 2119920).***
- ***For IL-1α c-aAb, see Svenson et al 1990 (PMID: 2270440), and Svenson et al 1992 (PMID: 1385986).***
- ***For IL-6 c-aAb, see Hansen et al 1993 (PMID: 8485307), Hansen et al 1995 (PMID: 7875195) and Galle et al 2004 (PMID: 15368270).***
- ***And for IL-10 c-aAb, see de Lemos Rieper 2009 (PMID:19632236) and de Lemos Rieper 2010 (PMID: 20638860).***

Ex-Vivo studies demonstrating neutralizing abilities of these four c-aAb have also been performed by Institute Pasteur, using DBDS samples (von Stemann et al 2025, PMID: 39551979).

We have also leveraged the fact that the immunoglobulin pools used for treating Danish patients are derived from plasma from Danish blood donors, to demonstrate how c-aAb introduced through IgG treatment can neutralize target GM-CSF, IL-1α, IL-6 and type 1 IFN activity in vivo (Ross et al 1995, PMID: 7738163, Ross et al 1997, PMID: 9310488, and Svenson et al 1998, PMID: 9490690).

More directly, we have also demonstrated how blood transfusion of plasma containing IL-6 c-aAb from Danish blood donors can neutralize IL-6 in vivo in the recipient patient (Hansen et al 2007, PMID: 17348870).

Taken together, there is solid evidence that the c-aAb we detect by the applied method in Danish blood donors are neutralizing in vivo.

Regarding c-aAb being activating/inhibitory:

This is an important and complex question, as an antibody's neutralizing capacity depends on the epitope specificity, affinity, binding capacity, and the Fc receptor-mediated clearance of cytokine-autoantibody immune complexes.

Briefly, the phenomena of "activating autoantibodies" may theoretically arise from the fact that cell-receptor binding of quite few cytokine molecules is fully sufficient to induce a substantial cytokine signal, as explored in Hansen 2020 (PMID: 32103575). This implies that in cases where the binding capacity of an in vitro neutralizing c-aAb is low compared to the concentration of its target cytokine, and cytokine-autoantibody complexes are not cleared efficiently, this could yield an in vivo "enhanced" delivering of the cytokine in question to target receptors, as an equilibrium is constantly established between the cytokine's binding to autoantibodies and cellular receptors (Hansen et al 1995, PMID: 7875195).

However, ultimately it is the bioactivity of the target cytokine that determines a true inhibitory/activating c-aAb-cytokine relationship; it is possible for a cytokine to be positively associated with c-aAb, and still be functionally compromised. For instance, our group has identified upregulated IL-6 in healthy blood donors with neutralizing IL-6 c-aAb (Galle et al 2004, PMID: 15368270), as well as simultaneous positive associations between IL-6 c-aAb and IL-6, and negative association between IL-6 c-aAb and IL-6 downstream biomarkers (von Stemann et al 2022, PMID: 35814772).

Furthermore, the current state of the field overwhelmingly present functional c-aAb effects on target cytokines as inhibitory, particularly in the context of clinical associations. Combined with our previously described studies in the Danish blood donor cohort, where we have not personally experienced cases of in vitro "activating" c-aAb, this leads us to consider that while the presence of "activating c-aAb" is theoretically possible, inhibitory effects are expected as the default.

Regarding whether any c-aAb were tested on more than one occasion:

We have in fact tracked levels of several c-aAb in a small subset of DBDS participants over time spanning up to a decade. For individuals with high titers of c-aAb (>90th percentile) at the first analyzed time point we observed the signals to remain above this threshold, and likewise, individuals with lower titers of c-aAb were seen to remain within this grouping. In addition, we have previously reported that high-titer c-aAb are capable of enduring a stem-cell transplantation (von Stemann et al 2021, PMID: 34907183). These findings show the potential stability of c-aAb titers, which underscores their potential clinical relevance (compared to if they had been transient). We have added these longitudinal results from our cohorts as a new supplementary figure 1 to the manuscript, to corroborate our existing findings.

2. It is very reasonable to have run this analysis on the most homogeneous sample possible. However, were other ethnicities available and explored? This is particularly of interest with the IFNg-HLA story from Southeast Asia.

While this is an excellent suggestion, the DBDS/CHB cohorts applied in the present study contain too few individuals of Southeastern Asian ethnicity/heritage to render analyses within such a sub-cohort feasible. However, we would be highly interested in pursuing c-aAb studies in Southeastern Asian cohorts and would welcome any offers for collaboration in this regard.

3. Did any of these antibody levels correlate with COVID exposure?

We have indeed observed an association between the analyzed c-aAb and COVID-19. Leveraging our recent "COVID-19 immunopathology" study, a prospective clinical multicenter study, including hospitalized patients in the beginning of the COVID-19 pandemic (Svanberg et al 2022, PMID: 36101705), we analyzed seven different c-aAb in 183 COVID-19 hospitalized patients at baseline and during their hospital stay. We observed a positive association between increased clinical severity score at admission and high titers of IFNα c-aAb (MFI>90th percentile), with 52.6% of IFNα c-aAb high-titer cases having a maximum severity score of 3, versus 17.1% in the remaining patients (p-value = 0.002, fishers exact test -see figure below). Upon sex-stratification, this association was specific for male patients (50% high titer cases with severity = 3, p-value = 0.012, fishers exact test).

These findings of a male-specific association of IFNα c-aAb to COVID-19 severity are in line with the results published by Bastard et al 2020 (PMID: 32972996).

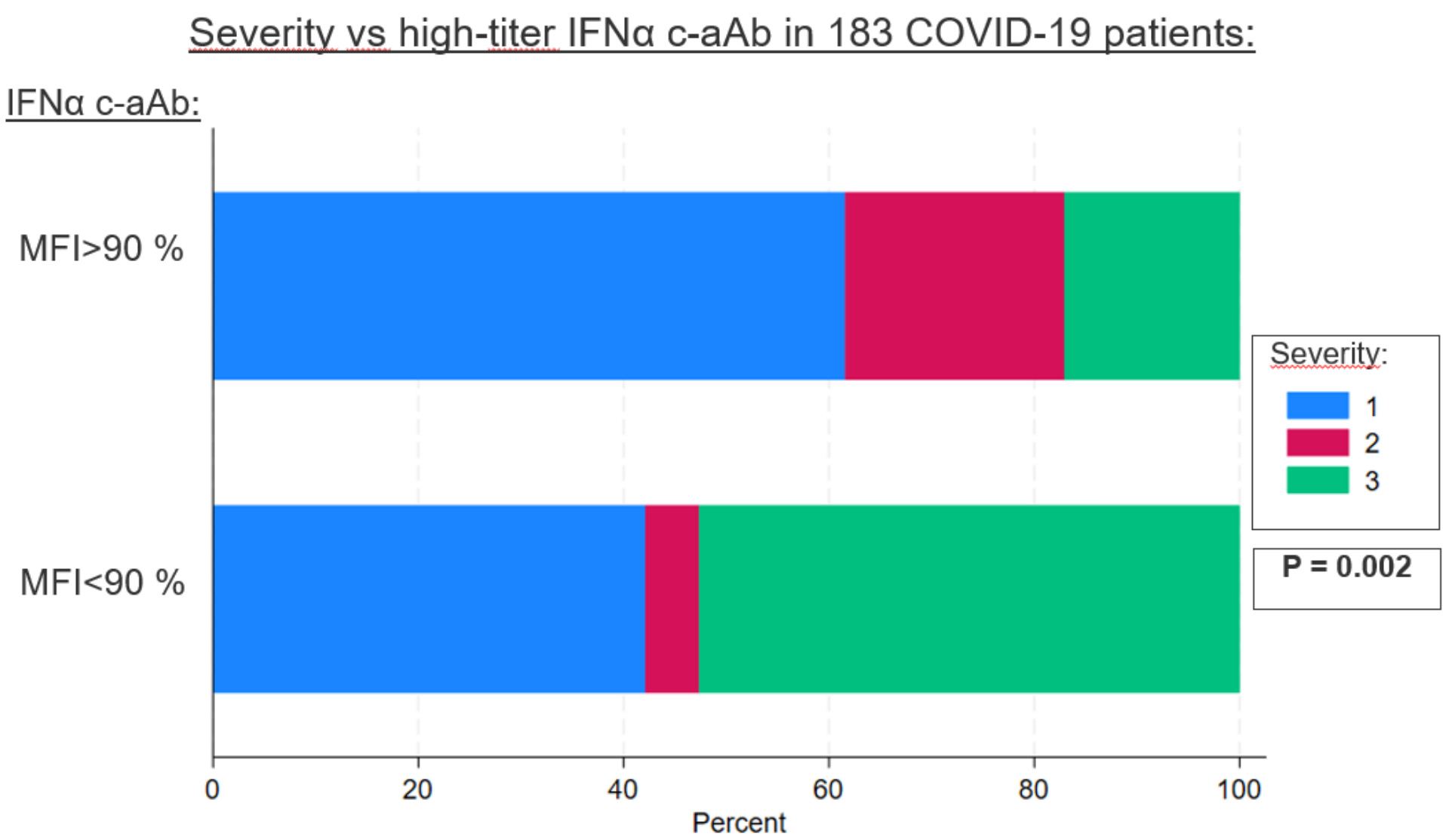

Reviewer #3 (Remarks to the Author):

Major Comments

This study represents a rare and valuable opportunity to analyse anti-cytokine autoantibodies in a very large, population-based cohort. The unbiased GWAS approach and agnostic binding assays are important strengths. Although the HLA associations are presented as novel, similar links between HLA alleles and autoantibody development have been reported previously and consistently. I suggest the authors temper their claims of novelty and instead highlight how their work complements or extends prior studies.

We acknowledge that prior studies have, for instance, found associations of IFNγ c-aAb to HLA, as well as links between IL-6 c-aAb and diabetes, which our present findings complement. However, we respectfully do still ultimately consider our findings novel both

due to our development of polygenic risk scores and testing of these against disease risk, as well as extending HLA associations to c-aAb with other specificities. We also feel that the striking differences revealed in genetic predictors (and subsequent risk scores) across the various c-aAb, are novel points of interest. However, we have toned down the phrasing of the manuscript text in this regard.

1. It would be helpful if the authors could provide, in the tables, the absolute numbers of individuals who were positive and negative for each anti-cytokine autoantibody in both the discovery and validation cohorts. This would allow readers to better gauge the prevalence and interpret the robustness of the genetic associations.

We agree that the distribution of c-aAb in the cohorts is important for the sake of context for the reader and acknowledge that our manuscript draft was lacking in providing this. The first paragraphs of the results section have now been reordered and rewritten to highlight the presence of discovery/validation cohort c-aAb distributions and high-titer cutoff thresholds in supplementary tables 1 and 2.

2. Because the assays used here detect only binding, the functionality of the detected autoantibodies is unknown. The clinical relevance of anti-cytokine autoantibodies usually hinges on whether they are neutralizing (e.g., anti–IFN-γ, anti–GM-CSF) or enhancing cytokine stability (e.g., anti–IL-6). Without functional data, the implications of detecting a binding antibody at a single time point are less clear. Non-neutralizing or transient antibodies may dilute the signal from high-titer, pathogenic neutralizing antibodies, potentially obscuring associations.

We discuss matters of c-aAb neutralization, antagonistic versus agonistic effects and stability in our response to reviewer #2 comment #1, and have added elements of this response, including its references, to the manuscript. Briefly, our previous work has demonstrated the in vivo neutralizing ability of c-aAb derived from healthy blood donors. Furthermore, our longitudinal c-aAb data, which have been added as supplementary results to the manuscript, demonstrates the capacity for years long stability of high titer c-aAb in healthy individuals, and thus increased likelihood of clinical relevance. As the functional effects of c-aAb are ultimately reliant on a complex interplay of epitope specificity, affinity, binding capacity, and antibody-cytokine complex clearance, exact clinical relevance can truly only be determined on a case-by-case basis, which is unfeasible for large-scale cohort studies. While we acknowledge the room for further sub-categorizing c-aAb beyond MFI signal, the present data still reveals genetic c-aAb predictors as markers of disease, indicating the potential capturing of clinical effects at this stage.

3. It is striking that strong HLA signals were found for disease-protective antibodies such as anti–IL-1α, whereas for pathogenic neutralizing autoantibodies (anti–IFN-γ, anti–GM-CSF), associations were weaker than in prior studies. This may reflect limited numbers of individuals with truly pathogenic autoantibodies anti-IFN-γ and anti–GM-CSF in the presumably mainly Caucasian cohort, or the possibility that binding-based assays captured non-pathogenic low-titer entities. Providing details on the distribution of specificities, titers, age-associations and overlap with known disease-associated phenotypes e.g. pulmonary alveolar proteinosis would clarify interpretation.

On the matter of c-aAb distribution, we agree with the reviewer that this may be influenced by age, sex and ethnic composition of the cohort, and that for instance, conducting the study in a cohort of East Asian ancestry could have yielded a different IFNγ c-aAb distribution, and subsequent results. Likewise, it seems reasonable to assume that the prevalence of truly pathogenic c-aAb would be lower in our cohort as it explicitly selects for healthy individuals

(among blood donors), and thus possibly individuals with less disease-inflicting c-aAb. However, it is worth noting that neutralizing capacity of c-aAb does not scale linearly with MFI, and that determining whether c-aAb are pathogenic is highly context dependent; a c-aAb may present as non-pathogenic only until the advent of a specific disease, as seen with type 1 IFN c-aAb and COVID-19 (Bastard et al 2021, PMID: 34413139). The identification of GM-CSF c-aAb as a dominant, high-avidity component of IgG pools derived from healthy Danish blood donors also underscores the impact of this c-aAb in the population, despite a comparatively lower-skewed distribution (Svenson et al 1998, PMID: 9490690). We have elaborated on this in the discussion, and rewritten the results section to highlight the demographic data, including c-aAb distributions, in supplementary tables 1 and 2.

Regarding the testing of our c-aAb data against pre-established associated disease phenotypes, the low prevalence of diseases such as PAP would leave insufficient power based on our present DBDS and CHB cohorts. In addition, the lack of statistically significant PRS's for the investigated GM-CSF c-aAb would render us unable to conduct survival analyses for PAP even if this was not the case, with the same being true for our investigated IFN-targeting c-aAb. However, through our aim of using common diseases to more broadly assess the relevance of c-aAb on health in a population, we did corroborate existing links of IL-1α and IL-6 c-aAb to arthritis and diabetes.

Minor Comments

1. Balance between statistics and biology

The manuscript is heavily weighted toward statistical reporting. It would be helpful to include more context on the biological and clinical implications of the findings. For instance:
• Which genes were included in the GWAS-based polygenic risk scores?

We acknowledge the slant towards statistical reporting but feel that a gene-comprehensive discussion of causality is beyond the scope of the study at this point in time. However, for the sake of transparency, and to invite future research into relevance, we have now extracted genes forming the basis for our PRSs and included them as supplementary tables 27-32.

• What percentages of blood donors fell into each age category or percentile?

As requested, the following table shows the distribution of DBDS participants in our respective CHB-based age categories, derived from birthdate quantile (Age category 1 being the oldest and 4 being the youngest).

Percentage of DBDS participants in CHB-derived age categories					
Birthdate Quantile	***CHB cohort, forming age category base***				
	CHB-CDC	***CHB-CID***	***CHB-DEBD***	***CHB-OCMS***	***CHB-PDS***
Q1	***0.2%***	***0.5%***	***0.6%***	***0.6%***	***1.2%***
Q2	***10.2%***	***12.2%***	***12.5%***	***13.2%***	***17.3%***
Q3	***36.2%***	***35.5%***	***35.8%***	***36.3%***	***35.5%***
Q4	***53.5%***	***51.8%***	***51.1%***	***49.9%***	***45.9%***

• Were there demographic patterns (e.g., sex, age) that might explain variation in autoantibody prevalence, e.g. if the blood donors were mainly 20-40 year-old and women then the expected prevalence of neutralizing anti-IFN-alpha autoantibodies in the cohort would be low since it was the

reported prevalence of pathogenic anti-IFN-alpha autoantibodies was <1% in European cohorts aged <70 but increased to 2-6% in >70, and were higher in men.

We have previously established strong associations between age and sex and several c-aAb (von Stemann et al 2017, PMID: 28665954), and thus agree with the reviewer that it is possible for demographic factors to influence c-aAb titer distributions in a cohort. As noted by the reviewer, pathogenic IFNα c-aAb prevalence has been observed to increase at >70 years of age, likely resulting in a lower-skewed distribution in our cohort as the current age of DBDS eligibility is 18-70 years (median age 38.3 years in our IL-1α, IL-6, IL-10, IFNα and GM-CSF c-aAb DBDS discovery cohort). It is thus possible that future studies in e.g. older cohorts could reveal further genetic predictors of pathogenic c-aAb, though it must also be noted that c-aAb prevalence has been variable across different studies and methods, and that our present cohort still features highly stable high-titer c-aAb individuals, including for IFNα c-aAb as seen in the newly added supplementary figure 1. We have updated the discussion to reflect these important observations.

2. The first sentence in the abstract should be plural "Anti-cytokine autoantibodies (c-aAb) are immunomodulatory phenomena linked to the risk and severity of various diseases". ***Thank you for this comment, the sentence has now been corrected.***

3. Could the authors speculate on the clinical significance of MICC (MHC Class I Polypeptide-Related Sequence C) and IFNA10 (Interferon alpha-10) associations with anti-IL-10, since this is the only non-inflammatory cytokine studied in their panel?

In terms of biological/causal relevance, we can only make assumptions, as little or no literature exists on the association between these genes and IL-10. Studies have implicated the related pseudogenes MICA and MICB in autoimmune disease (López-Arbesu et al 2006, PMID: 17003176) and linked MICA and IL-10 (Serrano et al 2011, PMID: 20714339), but presently MICC specifically has no reported transcription, or physiological effect. Biological relevance for IL-10 c-aAb beyond being a genetic marker could thus be possible but seems unlikely. As a member of the pro-inflammatory Type 1 Interferon family, expression of IFNα10 and anti-inflammatory IL-10 is certainly linked, but no evidence exists of any specific relevance of IFNα10 to IL-10, or autoantibody development. However, type 1 interferons in general serve at least as markers of autoimmune disease, and IFNα therapy has been observed to exacerbate autoimmune manifestations (Biggioggero et al 2010, PMID: 20298125).

We cannot speak to the clinical significance of individual gene findings in our paper and consider individual gene explorations beyond its scope; however, we are very interested if the reviewer has any specific hypotheses regarding these genes and IL-10, and in general hope other researchers will help pick up on the potentially causal threads this paper reports.

REVIEWERS' COMMENTS

Reviewer #1 (Remarks to the Author):

I believe the authors have satisfactorily addressed most of my questions. I have only one remaining comment regarding their response to my suggestion about computing genetic correlations. My original motivation for this request was to better understand whether the genetics of autoantibody production could be causally linked to the phenotypes with which the authors report associations. While pleiotropy can arise simply by chance (given the limited number of genes in the genome)it may also reflect shared underlying biological mechanisms. However, I recognize that such analyses are beyond the scope of the present study, and I am not requesting additional work along these lines.

I would also encourage the authors to make an effort to provide easy access to the GWAS summary statistics generated in this study, as this would greatly facilitate transparency, reproducibility, and downstream use by the community.

***We thank the reviewer for their valuable high-level technical considerations, which we find to have much improved the manuscript. In accordance with reviewer suggestions, we have also decided to make the generated GWAS summary statistics publicly available, using the online Zenodo depository. They will be published and publicly available upon final manuscript acceptance, and can in the interim be seen here: https://ze-
nodo.org/records/19483219?preview=1&token=eyJhbGciOiJIUzUxMiJ9.ey-
JpZCI6IjQzYTQ4OTc4LWIyZDktNGU2Yi05Mzc4LWJlYjE4NDliYjg2ZSIsIm-
RhdGEiOnt9LCJyYW5kb20iOiJkYjQ4NWM0Y2UxNzdhNWN-
lMzkxYjJmMTVmMDQ5ZjcwZSJ9.owPQxE-ZRhOL-
SZJ0UjSnUsPv5F7hq__glGX404RAoVEMItloHOOJkgaEqOO-
nIGGpj7pGf_oZe1Roe0wK8b3rHQ.***

Reviewer #2 (Remarks to the Author):

The authors have made successful efforts to respond to reviewer comments.

Please note that the ethnic association for anti-IFNg autoantibodies is South East Asian, not South Asian (page 22 bottom of marked MSS).

We thank the reviewer for catching this error, it has now been corrected.

Reviewer #3 (Remarks to the Author):

It has been a pleasure to review the revised version of this manuscript. I commend the authors on the substantial improvements made to the work. The addition of noteworthy clinical information—specifically the longitudinal data demonstrating the stability of high-titer cytokine autoantibodies over a decade, the nuances of threshold setting to find shared and distinct associations in females and males and the breakdown of the PRS —provides significant depth to the study and better supports the authors' interpretations. The revisions have effectively addressed nearly all of my previous concerns, particularly:
1. The inclusion of heritability estimates outside the MHC region, which clarifies the concentration of genetic contributors within the HLA.
2. The multi-trait GWAS analyses, which offer valuable insights into the shared versus distinct genetic architectures of the various cytokines.
3. The increased transparency regarding the specific genes utilized in the polygenic risk score.
4. The thoughtful expansion of the Discussion to account for demographic and ancestry-related influences on autoantibody prevalence.

I have only one remaining minor request, for the purpose of complete transparency. While you have clearly defined the thresholds for the binary high/low titer c-aAb phenotypes (MFI >90 percentile for high-titer cases and MFI <75 percentile for low-titer controls), the absolute numbers of individuals classified into these categories for each cytokine are not explicitly stated. Could the absolute number of individuals classed as binary high/low titer for each cytokine be added to Supplementary Table 1? Providing these counts will allow readers to better interpret the robustness of the genetic associations and the prevalence of these phenotypes within your discovery and validation cohorts.

I believe the manuscript will be an excellent contribution to the field.

We thank the reviewer for the praise, as well as the prior comments that have led to the current, improved form of the manuscript. In accordance with the request, we have added the "low-titer" MFI thresholds, as well as the absolute "high-titer" and "low-titer" numbers for each cytokine autoantibody to supplementary tables 1 and 2.
